# Amino Acid Specificity of Ancestral Aminoacyl-tRNA Synthetase Prior to the Last Universal Common Ancestor *Commonote commonote*

**DOI:** 10.1007/s00239-021-10043-z

**Published:** 2022-01-27

**Authors:** Ryutaro Furukawa, Shin-ichi Yokobori, Riku Sato, Taimu Kumagawa, Mizuho Nakagawa, Kazutaka Katoh, Akihiko Yamagishi

**Affiliations:** 1 Department of Applied Life Sciences, School of Life Sciences, Tokyo University of Pharmacy and Life Sciences, 1432-1 Horinouchi, Hachioji, Tokyo, Japan; 2 Faculty of Human Science, Waseda University, 2-579-15 Mikajima, Tokorozawa, Saitama, 359-1192 Japan; 3 Department of Genome Informatics, Genome Information Research Center, Research Institute for Microbial Diseases, Osaka University, 3-1 Yamadaoka, Suita, Osaka, 565-0871 Japan

**Keywords:** Aminoacyl-tRNA synthetase, Phylogenetic analysis, Ancestral sequence reconstruction, Amino acid repertoire

## Abstract

Extant organisms commonly use 20 amino acids in protein synthesis. In the translation system, aminoacyl-tRNA synthetase (ARS) selectively binds an amino acid and transfers it to the cognate tRNA. It is postulated that the amino acid repertoire of ARS expanded during the development of the translation system. In this study we generated composite phylogenetic trees for seven ARSs (SerRS, ProRS, ThrRS, GlyRS-1, HisRS, AspRS, and LysRS) which are thought to have diverged by gene duplication followed by mutation, before the evolution of the last universal common ancestor. The composite phylogenetic tree shows that the AspRS/LysRS branch diverged from the other five ARSs at the deepest node, with the GlyRS/HisRS branch and the other three ARSs (ThrRS, ProRS and SerRS) diverging at the second deepest node. ThrRS diverged next, and finally ProRS and SerRS diverged from each other. Based on the phylogenetic tree, sequences of the ancestral ARSs prior to the evolution of the last universal common ancestor were predicted. The amino acid specificity of each ancestral ARS was then postulated by comparison with amino acid recognition sites of ARSs of extant organisms. Our predictions demonstrate that ancestral ARSs had substantial specificity and that the number of amino acid types amino-acylated by proteinaceous ARSs was limited before the appearance of a fuller range of proteinaceous ARS species. From an assumption that 10 amino acid species are required for folding and function, proteinaceous ARS possibly evolved in a translation system composed of preexisting ribozyme ARSs, before the evolution of the last universal common ancestor.

**Supplementary Information:**

The online version contains supplementary material available at 10.1007/s00239-021-10043-z.

## Introduction

Aminoacyl-tRNA synthetase (ARS) is an essential enzyme for translation in all extant organisms. ARS attaches an amino acid to the cognate tRNA, and the aminoacyl-tRNA is then used for translation upon binding to mRNA according to the codon–anticodon interaction on the ribosome. ARS catalyzes a two-step reaction: (1) formation of aminoacyl-AMP from amino acid and ATP, and; (2) Formation of aminoacyl-tRNA from aminoacyl-AMP and tRNA, resulting in the attachment of an amino acid to the cognate tRNA. ARS specifically selects and binds amino acid and tRNA. This step is crucial for translation whereby the correct amino acid is translated for the particular codon. As a further specificity measure, some ARSs also have an editing domain that hydrolyzes mischarged tRNA to prevent translational error.

At some time during or after the emergence of living systems on Earth, a genetic system encompassing the translation system developed. However, if specific binding between amino acid and tRNA is catalyzed by proteinaceous ARS, an intriguing puzzle about the development of the translation system presents itself: namely, how were the first active ARSs translated in the absence of proteinaceous ARSs? Some researchers have proposed that in the early stages of the history of life, protein or peptide synthesis was established by RNA, i.e., ribozymes (Härtlein and Cusack 1995; Wolf and Koonin 2007; Koonin 2017; Lei and Burton 2020). Indeed, it has been demonstrated that a ribozyme can attach an activated amino acid to a tRNA (Piccirilli et al. 1992; Illangasekare et al. 1995; Saito et al. 2001). These results imply that in the early stages of the evolution of the translation system, protein or peptide synthesis could be performed by a translation system using ribozyme ARSs without need of proteinaceous ARSs. After the emergence of a ribozyme with aminoacylation activity, a gradual transition from a ribozyme-ARS based translation system to a proteinaceous ARS may have occurred in the preexisting ribozyme-based translation system.

Many hypotheses have been proposed on the origin of the genetic code, particularly about how amino acids started interacting with RNA molecules (Crick 1968; Woese 1973; Wong 1975; Eigen and Schuster 1977; Wolf and Koonin 2007). Interactions between amino acids and codon bases or anticodon bases in RNA molecules have been experimentally detected. This implies that the direct interaction between amino acids and primitive tRNA may be responsible for the origin of the genetic code (reviewed in Yarus 2017).

Another question about the evolution of the translation system and genetic code is: How many amino acid types were required for the first ARS protein with activity? This relates to the structural requirements of the primitive proteins. The number of amino acid types required to provide proteinaceous structure and activity has been investigated experimentally (Davidson et al. 1995; Riddle et al. 1997; Murphy et al. 2000; Akanuma et al. 2002; Walter et al. 2005; Longo et al. 2013; Shibue et al. 2018; Kimura and Akanuma 2020; reviewed in Longo and Blaber 2012). A pre-biotic set of amino acids was proposed by Longo and Blaber (2012), based on meteorite information, spark experiments and hydrothermal experiments. They proposed a pre-biotic amino acid set: Ala, Asp, Glu, Gly, Ile, Leu, Pro, Ser, Thr, and Val. Recent experiments on amino acid type-simplified proteins (Longo et al. 2013; Shibue et al. 2018; Kimura and Akanuma 2020) confirmed that about 10 pre-biotic amino acid types could build stable protein structures. Although threonine, serine, and isoleucine are not always necessary to build a structural protein, histidine is necessary for catalytic activity. This suggests that a functional amino acid such as histidine or a functional RNA is required, if a stable protein consisting of pre-biotic amino acid sets is to acquire catalytic activity (Shibue et al. 2018; Kimura and Akanuma 2020).

**Fig. 1:**
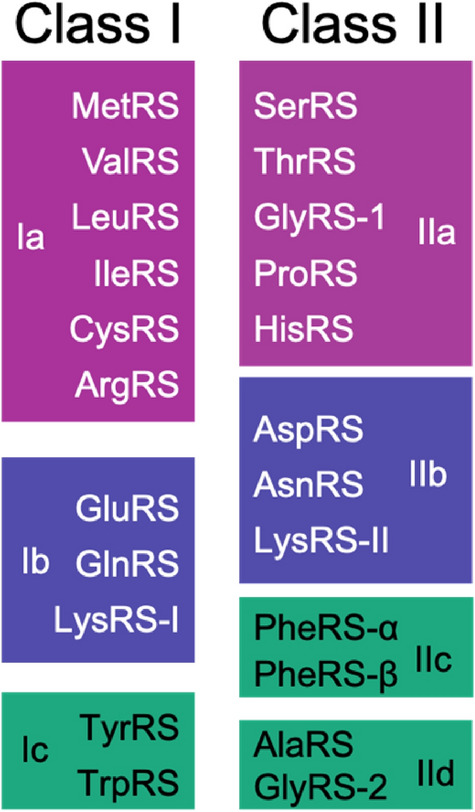
Classification of aminoacyl-tRNA synthetases ( modified from De Pouplana and Schimmel 2000, Valencia-Sánchez et al. 2016)

A little more than 20 ARSs are found in contemporary organisms. They are classified into two groups, class I and class II (Fig. 1), each having three subclasses (a–c) based on similarity in sequences and structures (Eriani et al. 1990). The classification is as follows: class Ia (MetRS, ValRS, LeuRS, IleRS, CysRS, and ArgRS); class Ib (GluRS, GlnRS and LysRS-class I); class Ic (TyrRS and TrpRS); class IIa (SerRS, ThrRS, AlaRS, GlyRS-α_2_, ProRS, and HisRS); class IIb (AspRS, AsnRS, and LysRS-class II); and class IIc (PheRS, GlyRS-α_2_β_2_, SepRS, and PylRS). A recent study of a root mean structural distance (RMSD) cluster dendrogram of ARS resulted in the proposition that PheRS, SepRS, and PylRS should be classified as class IIc and AlaRS and GlyRS-α_2_β_2_ as class IId (Valencia-Sánchez et al. 2016). Furthermore, AlaRS was removed from class IIa and reclassified into class IId together with GlyRS-α_2_β_2_ (Fig. 1). In this study we refer to GlyRS-α_2_ as GlyRS-1 and GlyRS-α_2_β_2_ as GlyRS-2. In general, ARS consists of a catalytic domain, an anticodon-binding domain, and often an editing domain. Each class harbors class-specific characteristic motifs and structural topology in its catalytic domains (Eriani et al. 1990).

Since all known organisms use 20 standard amino acids in translation, the last universal common ancestor is proposed to have been using the same 20 standard amino acids in translation as contemporary living systems (Woese et al. 2000; Akanuma 2019). Some phylogenetic analyses of ARS have suggested that the diversification of ARSs of each class occurred in the era before the last universal common ancestor of all extant organisms (Nagel and Doolittle 1991, 1995; Brown 2001, 2003). A structural dendrogram of each class has also been used to suggest an evolutionary scheme for each ARS (O’Donoghue and Luthey-Schulten 2003). These analyses provided important information that can be used to trace the ancestors of class I and class II ARSs before the last universal common ancestor.

The origin and evolution of ARSs since the last universal common ancestor is complex, resulting from various events including gene losses, gene duplications, lateral gene transfers, and replacements of other genes (Wolf et al. 1999; Woese et al. 2000; Brindefalk et al. 2007; Furukawa et al. 2017). Although the volume of data on ARSs is increasing, detailed composite trees for ARSs of each class have not been reported. Although Andam and Gogarten reported a composite tree for class II ARSs, they used a limited number of species in their comparison (2011). A detailed composite tree with more taxonomical entries is required, to clarify the process of ARS evolution.

Some ancestral sequence reconstructions have provided information about the ancestral history before the divergence of individual ARSs. The common ancestral sequence of IleRS and ValRS was estimated from a composite tree of IleRS, ValRS, and LeuRS (Fournier et al. 2011). The common ancestral sequence contained isoleucine and valine, with a high probability at sites where these residues are conserved in contemporary IleRS and ValRS, suggesting that the protein synthesis system at that time could have used both isoleucine and valine. The common ancestral protein sequence of TyrRS and TrpRS was reconstructed by Fournier and Alm (2015). The sequence did not contain tryptophan residues, which suggests the late addition of tryptophan to the genetic code, after the divergence of TyrRS and TrpRS. Thus, sequence reconstruction of ancestral ARSs provides information about the ancestral translation system in use prior to the evolution of the last universal common ancestor.

As a means of experimental research, Urzymes have been constructed (Pham et al. 2007, 2010; Li et al. 2011). They are the truncated modules of the conserved catalytic site from contemporary class I and class II ARSs, consisting of 120–130 amino acid residues. Urzymes activate cognate amino acids at a lower rate than corresponding ARSs, which suggests the possibility of primitive ARSs consisting of short fragments of the catalytic domain. Protozymes, which consist of the truncated module of ATP binding sites from class I ARS Urzymes, and which comprise 46 amino acid residues, have also been constructed (Martinez-Rodriguez et al. 2015). Protozymes activate cognate amino acids at lower rates than Urzymes. Because these minimal ARSs were constructed using contemporary or artificial sequences, amino acid specificity during ARS evolution cannot be investigated. However, the effort of identifying the minimal structure of ARSs suggests that we should be able to trace the primitive ARSs which consisted of fewer than 50 amino acid residues.

Other aspects of the evolution of the translation system have also been investigated. The order of recruitment of amino acids into the protein synthesis system has been proposed, based on estimated codon usage bias during the phylogenetic speciation of living organisms (Liu et al. 2010). Quantum chemical calculations of amino acids, and biochemical experiments with amino acids on animal membrane surfaces, suggested that tyrosine and tryptophan were added to the genetic code to prevent oxidative stress during the rise in concentration of molecular oxygen in the biosphere (Granold et al. 2018). The order or recruitment of amino acid into the protein synthesis system has also been proposed, based on amino acid properties (Francis 2013), on the amino acid frequency in ancestral sequences (Jordan et al. 2005), and on consideration of 60 other factors (Trifonov 2004). However, more sequence-based evidence about the route of evolution is required. Here, we have tried to trace back to an ancestral ARS present before the last universal common ancestor, and to determine its amino acid specificity.

In this study we have reconstructed the history of class IIa ARSs (SerRS, ThrRS, GlyRS-1, ProRS, and HisRS) and class IIb ARSs (AspRS and LysRS). We have estimated the amino acid sequences of ancestral ARSs prior to the evolution of the last universal common ancestor, based on composite tree analyses and ancestral sequence reconstruction. We concentrated on the amino acid specificity of ancestral ARS, estimated from amino acid recognizing residues of ancestral sequences. Our study provides information on the process of incorporating different amino acids into the translation system, at a time before the last universal common ancestor. Recent reviews of the last universal common ancestor can be found elsewhere (Akanuma 2017, 2019; Cantine and Fournier 2018; Weiss et al. 2018). The last universal common ancestor is referred to differently in different publications; we refer to it as *Commonote commonote* (Akanuma et al. 2015; Akanuma 2017) in this report.

## Materials and Methods

### ARS Sequence Data

Protein sequences of 23 archaeal species and 57 bacterial species were collected from the National Center for Biotechnology Information (NCBI; https://www.ncbi.nlm.nih.gov). We constructed a KF database (Furukawa et al. 2017) consisting of all protein sequences from these 80 organisms on October 2011, and named it KF ver.2011. The KF database was updated to 392 organisms (92 archaeal species, 300 bacterial species) in April 2021; it was named KF ver. 2021. The amino acid sequences of seven ARSs (class IIa: SerRS, ThrRS, GlyRS-1, ProRS, and HisRS; and class IIb: AspRS and LysRS) in the two databases (KF ver. 2011 and KF ver. 2021) were searched with BlastP (Altschul et al. 1997). Accession numbers of all collected data are shown in Supplemental Tables S1 (KF ver. 2011) and S2 (KF ver. 2021).

### Sequence Alignment

The collected amino acid sequences were classified into class IIa ARSs (SerRS, ThrRS, GlyRS-1, ProRS, and HisRS) or class IIb ARSs (AspRS and LysRS). Amino acid sequences of each subclass of ARS were aligned using MAFFT 7.293 (Katoh and Standley 2013) with MAFFT-ash. Since MAFFT-ash requires reference PDB (Protein Data Bank) structure files, we selected six PDB structures of ARSs (1ATI: *Thermus thermophilus* GlyRS-1; 1H4V: *T. thermophilus* HisRS; 1QF6: *Escherichia coli* ThrRS; 1NJ8: *Methanocaldococcus jannaschii* ProRS; 1EQR: *E. coli* AspRS; and 1E1O: *E. coli* LysRS). Some poorly aligned regions were realigned with a Ruby script for regional realignment in MAFFT (https://mafft.cbrc.jp/alignment/software/regionalrealignment.html). After computational alignment, we manually adjusted the alignment based on the corresponding secondary structures of ARSs. Generally, the N-terminal domain of SerRS binds the variable arm of tRNA^ser^, which is the critical interaction between SerRS and tRNA^ser^. HisRS, GlyRS-1, ProRS, and ThrRS have an anticodon binding domain in the C-terminal region, but SerRS does not. From these facts, we hypothesized that the N-terminal tRNA binding domain of SerRS originated from the C-terminal anticodon binding domain before the diversification between Archaea and Bacteria and after the diversification from the common ancestor of SerRS and other ARSs. To test this hypothesis, the N-terminal tRNA binding domain of SerRS was truncated and pasted onto the C-terminus of SerRS. Regional realignment was performed in the region of the C-terminal anticodon binding domain. A composite alignment of seven ARSs was constructed using the above procedure, and a composite alignment of five ARSs was constructed by removing AspRS and LysRS sequences from the composite alignment of seven ARSs.

The well-aligned regions of each alignment were selected from the final alignment using TrimAl 1.4 (Capella-Gutiérrez et al. 2009). TrimAl was used in automated1 mode. The gap-containing columns in the result in automated1 mode were extracted, using the nogaps mode. The number of remaining sites in the final alignment (KF ver. 2011) was 143 amino acid sites in the seven ARSs alignment, consisting of 380 sequences, and 194 amino acid sites in the five ARSs alignment, consisting of 257 sequences. The number of remaining sites in the final alignment (KF ver. 2021) was 135 amino acid sites in the seven ARSs alignment, consisting of 1,006 sequences. The 7ARSs final alignment of KF ver. 2011 was named alignment A (Supplemental Table S3b), and the 7ARSs final alignment of KF ver. 2021 was named alignment B (Supplemental Table S3c).

### Phylogenetic Analysis

Composite trees of each subclass of ARS were reconstructed by maximum likelihood (ML) and Bayesian inference (BI) analyses. ML analysis was performed with the IQ-TREE 1.6.9 program (Nguyen et al. 2015), using an optimal amino acid substitution model (7ARSs alignment A: LG + R7, 5ARSs: LG + R5; 7ARSs alignment B: LG + R9) selected by ModelFinder (Kalyaanamoorthy et al. 2017). An ultrafast bootstrap analysis (Hoang et al. 2018) was also performed in IQ-TREE analysis. Subsequently, a standard bootstrap analysis was performed in IQ-TREE analysis. A posterior probability consensus tree in BI analysis was constructed using PhyloBayes 4.1c (Lartillot et al. 2009), running two chains until the maximal discrepancy dropped below 0.3 on the CAT Poisson + G(4) model. The consensus tree was outputted using the readpb program. The trees used in the readpb analysis were sampled every 10 generations in each analysis.

### Ancestral Sequence Reconstruction

The ancestral sequences of each node in seven class II ARS trees (ML tree and Bayesian tree) were estimated with Codeml in PAML 4.9i (Yang 2007), IQ-TREE, nhPhyloBayes 0.2.3 (Blanquart and Lartillot 2008), RAxML 8.1.12 (Stamatakis 2014), and PhyML 3.3 (Guindon et al. 2010). Codeml was performed under LG + G(8). IQ-TREE was performed under LG + R7 for 7ARSs alignment A and LG + R9 for 7ARSs alignment B. nhPhyloBayes was performed under a CAT + BP model. RAxML was performed under a PROTGAMMALG model. PhyML was performed under a LG + G model. GASP (Edwards and Shields 2004) was used to infer the ancestral gap sites at the ancestral node on the phylogenetic tree. The ancestral sequences of the divergent point (ancestral node) of each ARS were picked up. In the ML tree from final alignment A, the amino acid sequences and per site posterior probability of AncDK, AncHGSPT, AncHG, AncSPT, and AncSP were estimated. AncDK is the common ancestor of AspRS and LysRS. AncHGSPT, AncHG, AncSPT, and AncSP were abbreviated in the same way. The amino acid sequences and per site posterior probability of ComD, ComK, ComH, ComG, ComT, ComP, and ComS were estimated. ComD is the AspRS of the last common ancestor of all living organisms *C. commonote*. ComK, ComH, ComG, ComT, ComP, and ComS were abbreviated as for ComD. In the Bayesian tree from final alignment A and the ML tree from final alignment B, the amino acid sequences and per site posterior probability were estimated of AncDK, AncHGSPT, AncHSPT, AncSPT, AncSP, ComK, ComH, ComG, ComT, ComP, and ComS.

### Estimation of Amino Acid Specificity of Contemporary ARS and Ancestral ARS

We define the predicted amino acid specificity (PAAS) as the degree of conservation of the substrate amino acid interacting residues in an ARS. The amino acid residues interacting with substrates in ARSs have been described in reports of the crystal structures of respective ARSs (Belrhali et al. 1995; Åberg et al. 1997; Arnez et al. 1997, 1999; Schmitt et al. 1998; Eiler et al. 1999; Sankaranarayanan et al. 2000; Onesti et al. 2000; Crepin et al. 2006; Bilokapic et al. 2006), and are summarized in Table 1 and Supplemental Table 4, and shown in Supplemental Figures S2A–H. PAAS is estimated as follows. The number of amino acid residues interacting with a side chain or amino group of substrate amino acids directly or through zinc ion binding with a distance of less than 3.0 Å in an ARS is denoted as *n*_*0*_. The number of conserved residues among *n*_*0*_ found in another ARS is denoted as *n*. The PAAS is then defined as *n*/*n*_*0*_ in the ARS in question. Based on our definition, the PAAS of a contemporary ARS for cognate amino acid is defined as full conservation; i.e., PAAS = 1 (Table 2).

**Table 1:**
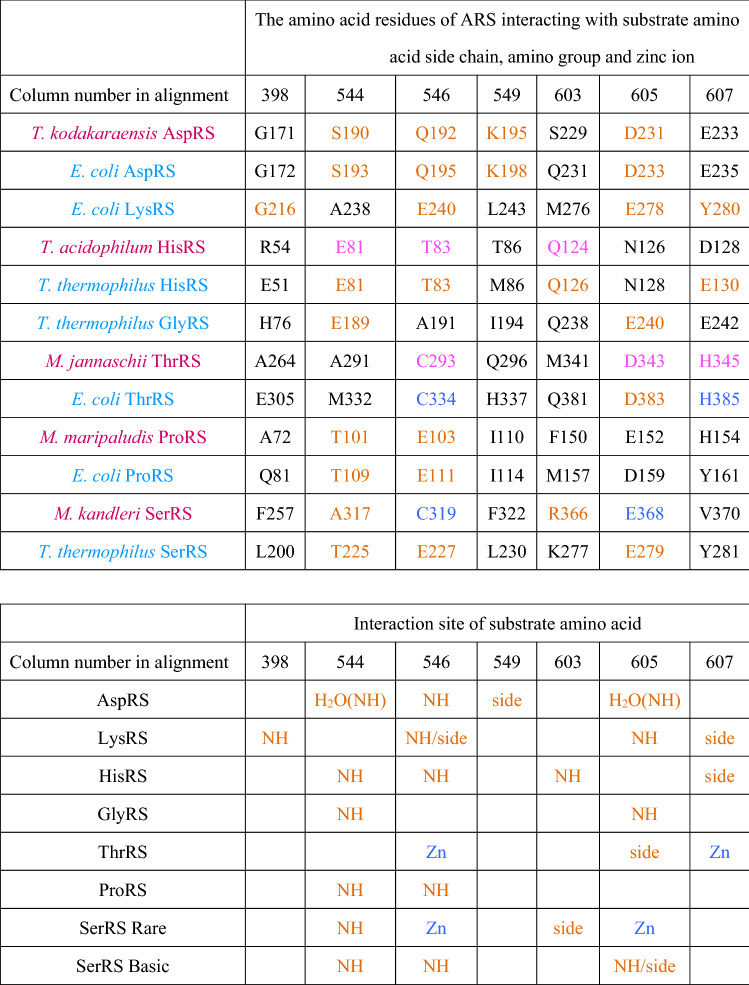

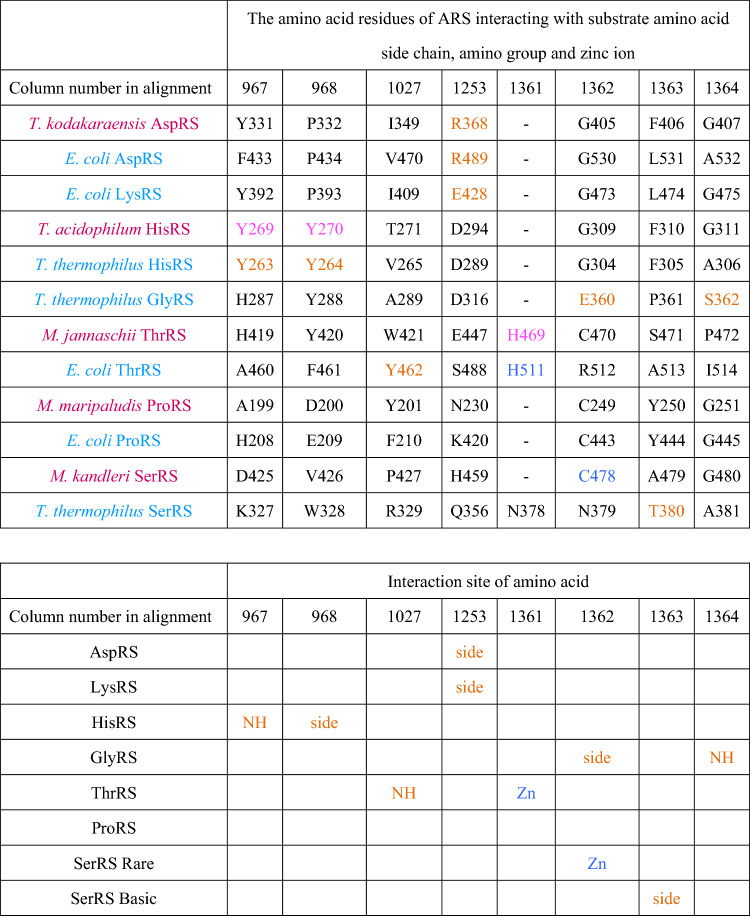
Substrate amino acid interaction sites of extant ARS, interacting portion of the substrate amino acid and indication of presence of ion in respective contemporary ARSs

Archaea and Bacteria are shown in red and pale blue, respectively. The amino acid residues interacting with substrate amino acid side chain and a zinc ion in crystal structures of contemporary ARSs are shown in orange and blue, respectively. The corresponding residues in other ARSs are shown in black. The amino acid residues colored pink are predicted to interact with a substrate amino acid side chain, amino group and a zinc ion from the structure of homologous ARS. The number at the top of column indicates the column number in alignment used in phylogenetic analyses (Supplemental Table S3b)

**Table 2:**
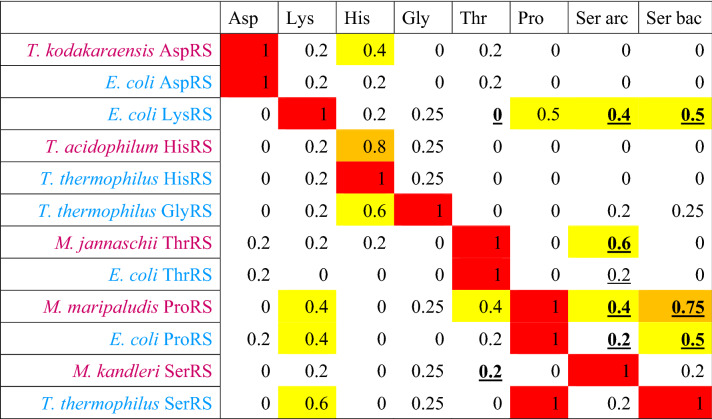
The conservation of substrate recognition residues in contemporary ARSs

Predicted amino acid specificities (PAASs) higher than 0.8 are highlighted in red. PAASs lower than 0.8 and higher than 0.6 are highlighted in orange. PAASs between 0.6 and 0.4 are highlighted in yellow. PAAS values with experimental evidence supporting the binding of noncognate amino acid are in bold and underlined

The amino acid specificities of ancestral ARSs for seven amino acids were estimated, based on the average posterior probability of ancestral amino acid residues responsible for each substrate amino acid recognition. The average posterior probability of the substrate recognition residues is abbreviated as APP-SRR. When the ancestral amino acid residue is identical to the residue involved in the substrate specificity of a contemporary ARS (Table 1, Supplemental Table S4), the posterior probability of the ancestral residue is considered to have the same degree of contribution to the amino acid specificity in the ancestral ARSs (Table 3, Supplemental Table S5). When the ancestral amino acid residue differs from the residue involved in the substrate specificity of a contemporary ARS, the posterior probability of the ancestral residue was assumed to be zero. These values were averaged in APP-SRR for each ancestral ARS against seven substrate amino acid species.

**Table 3:**
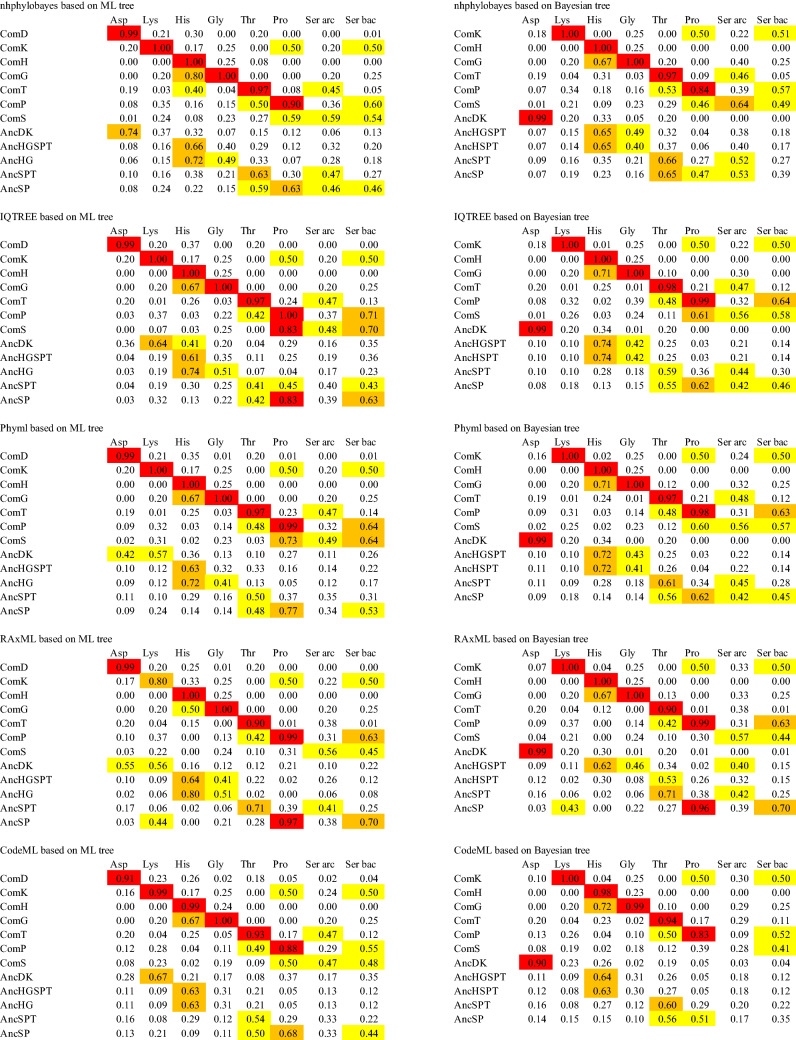

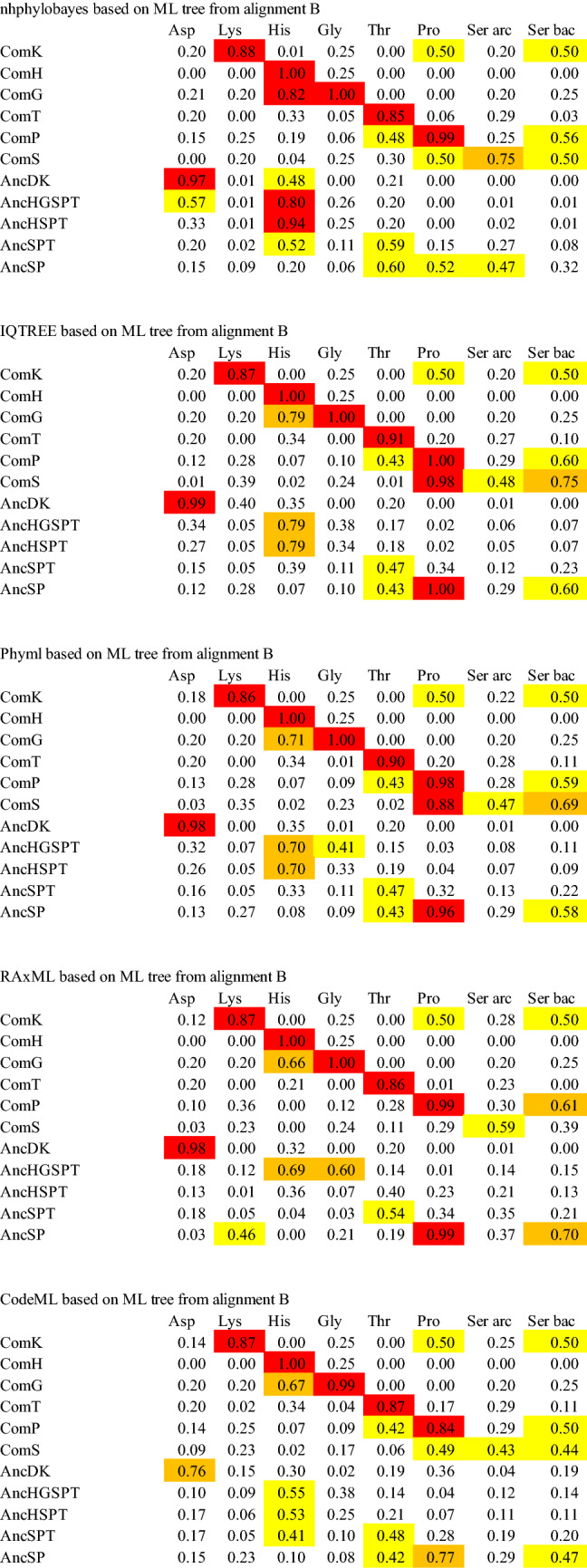
Average posterior probability of the substrate recognition residues (APP-SRR) in ancestral ARSs

APP-SRRs higher than 0.8 are highlighted in red. APP-SRRs lower than 0.8 and higher than 0.6 are highlighted in orange. APP-SRRs between 0.6 and 0.4 are highlighted in yellow

## Results

### Roots of Class IIa and Class IIb ARSs

Amino acid sequence alignments of class IIa and class IIb ARSs are shown in Supplemental Table S3. They have several common conserved domains and motifs, including the catalytic domain, motif 1, motif 2, and motif 3. The anticodon binding domain is conserved in several class IIa ARSs (HisRS, GlyRS-1, ThrRS, and ProRS).

**Fig. 2:**
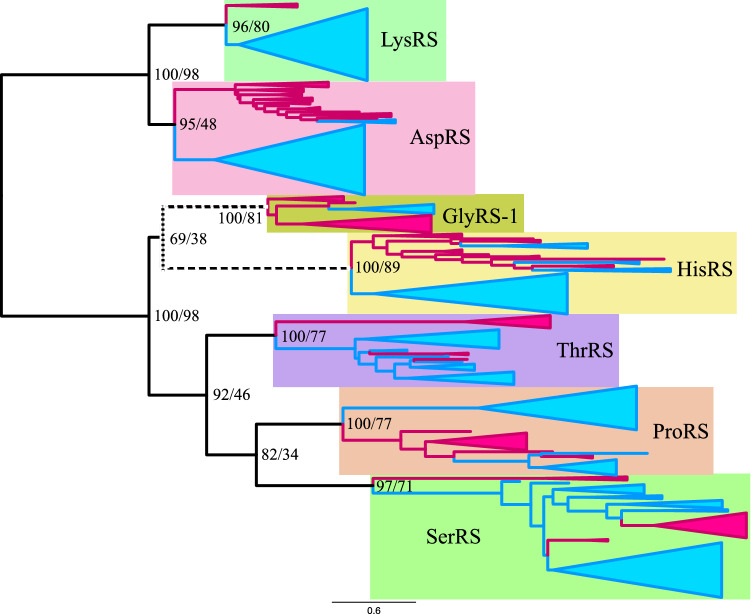
Maximum likelihood composite tree of class IIa ARSs (HisRS, GlyRS-1, ThrRS, ProRS, and SerRS) and class IIb ARSs (LysRS and AspRS) from alignment A. Archaeal branches and bacterial branches are colored in magenta and blue, respectively. The scale bar indicates the number of substitutions per site. Numbers on each node indicate RELL bootstrap value/standard bootstrap value, respectively. Log likelihood of tree was − 60,237.4

**Fig. 3:**
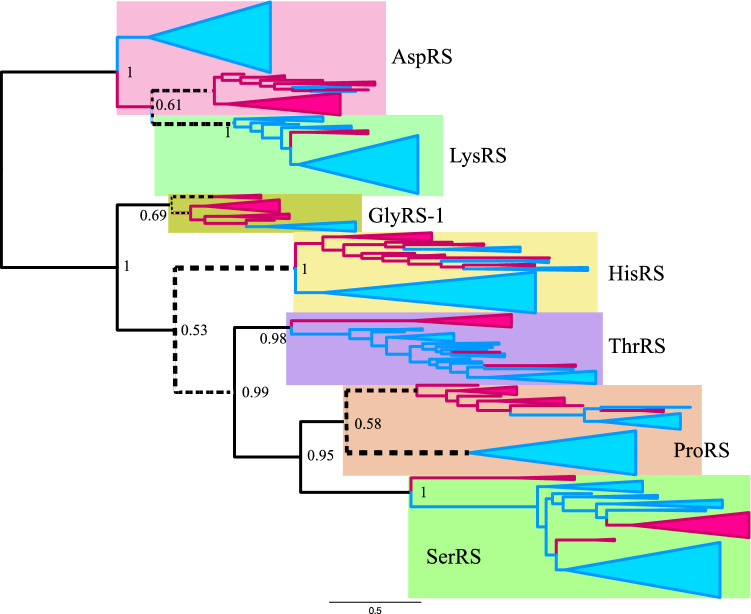
Bayesian composite tree of class IIa ARSs (HisRS, GlyRS-1, ThrRS, ProRS, and SerRS) and class IIb ARSs (AspRS and LysRS) from alignment A. Archaeal branches and bacterial branches are colored in magenta and blue, respectively. The scale bar indicates the number of substitutions per site. Numbers on each node indicate posterior probability. Log likelihood was − 61,416.4

**Fig. 4:**
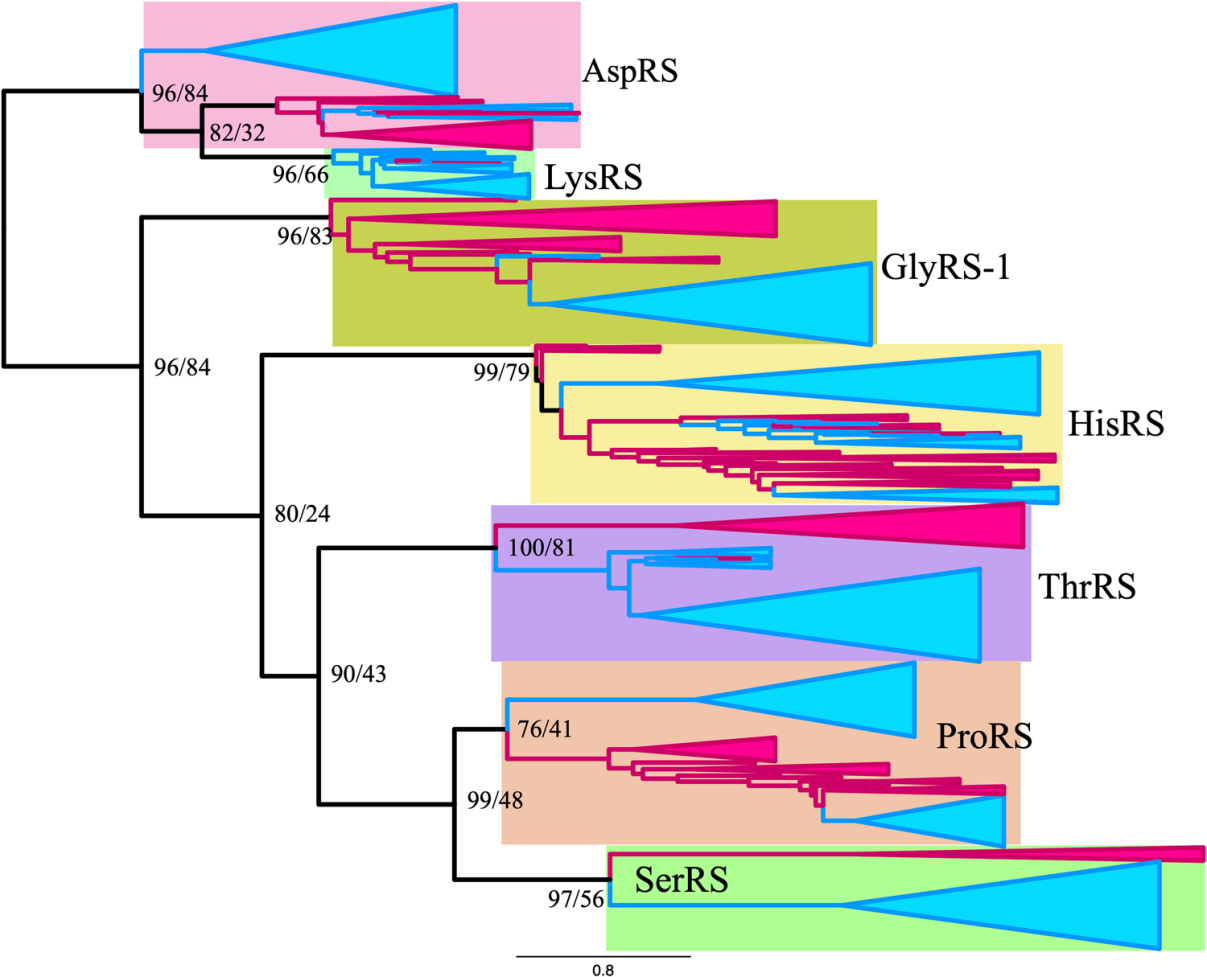
Maximum likelihood composite tree of class IIa ARSs (HisRS, GlyRS-1, ThrRS, ProRS, and SerRS) and class IIb ARSs (LysRS and AspRS) from alignment B. Archaeal branches and bacterial branches are colored in magenta and blue, respectively. The scale bar indicates the number of substitutions per site. Numbers on each node indicate RELL bootstrap value/standard bootstrap value, respectively. Log likelihood of tree was − 143,910.9

To define the root of class IIa ARSs, composite trees for class IIa and class IIb ARSs were reconstructed using ML and BI methods (Figs. 2, 3 and 4). In the ML tree from alignment A, the root of class IIa ARSs was found between the HisRS/GlyRS-1 branch and the ThrRS/ProRS/SerRS branch, when class IIb ARSs were used as outgroups. The root of class IIb ARSs was found between AspRS and LysRS. In the ML tree (Fig. 2), monophyly of class IIa and class IIb ARSs was supported with 100% RELL bootstrap probability (rbp) and 98% bootstrap probability (bp) respectively. In the Bayesian tree from alignment A (Fig. 3) and the ML tree from alignment B (Fig. 4), the root of class IIa ARSs was found between the GlyRS-1 branch and the other four ARSs. The root of class IIb ARSs was found in AspRS. Monophyly of class IIa and class IIb ARSs was supported with 1.00 posterior probability (pp) in a Bayesian tree, and supported with 96% rbp and 84% bp in the ML tree from alignment B.

### Phylogenetic Relationship in Class IIa and Class IIb ARSs

The ML tree from alignment A demonstrates that the HisRS/GlyRS-1 branch diverged earliest and that ThrRS diverged second (Fig. 2). The monophyletic group of ProRS and SerRS diverged third. In the Bayesian tree from alignment A and the ML tree from alignment B, the GlyRS-1 branch diverged earliest, HisRS second, ThrRS third, and ProRS and SerRS last. The sisterhood of ProRS and SerRS was supported with 82% rbp and 34% bp in the ML tree from alignment A and 0.95 pp in the Bayesian tree, and 99% rbp and 48% bp in the ML tree from alignment B. This monophyletic relationship of ProRS and SerRS was supported in the composite tree of class IIa ARSs lacking class IIb (Supplemental Fig. S1). The monophyletic relationship of ThrRS, ProRS, and SerRS was supported in both the ML and Bayesian trees from alignment A and the ML tree from alignment B, with 92% rbp and 46% bp, 0.99 pp, and 90% rbp and 43% bp, respectively. The sisterhood of HisRS and GlyRS-1 was weakly supported with 69% rbp and 38% bp in the ML tree from alignment A. In the Bayesian tree and ML tree from alignment B, the sisterhood of HisRS and GlyRS-1 was not supported, and the monophyletic group HisRS/ThrRS/ProRS/SerRS was weakly supported with 0.53 pp, 80% rbp and 24% bp. The monophyly of HisRS, GlyRS-1, ThrRS, ProRS, and SerRS was strongly supported with 100%, 100%, 100%, 100%, and 97% bp respectively in the ML tree from alignment A. The same was true for HisRS, ThrRS, and SerRS in the Bayesian tree from alignment A. The phylogenetic relationship of AspRS and LysRS differs between the ML tree from alignment A and other trees (the Bayesian tree from alignment A and the ML tree from alignment B). In the ML tree from alignment A, AspRS and LysRS were monophyletic, whereas in the Bayesian tree from alignment A and the ML tree from alignment B, LysRS diverged from AspRS, implying that AspRS is an ancestor of LysRS. The monophyly of AspRS was strongly supported with 95% rbp in the ML tree from alignment A. However, the monophyly of LysRS and archaeal AspRS was supported with 0.61 pp in the Bayesian tree from alignment A and with 82% rbp and 32% bp in the ML tree from alignment B. Our trees tend to support the latter case, that LysRS was derived from archaeal AspRS.

### Root Position of Each Class IIa ARS and Class IIb ARS

Each of the AspRS, HisRS, ThrRS, ProRS, and SerRS branches was rooted between the Bacteria and Archaea groups in both the ML and Bayesian analyses of alignments A and B, except for HisRS in the ML tree from alignment B, which was rooted in the archaeal group. Thus, five ARSs supported the proposal that the position of *C. commonote* is between the Bacteria and Archaea/Eukarya groups, as reported in many previous publications (Iwabe et al. 1989; Brown and Doolittle 1995; Lawson et al. 1996; Labedan et al. 1999). Previous composite trees (Nagel and Doolittle 1991, 1995; Härtlein and Cusack 1995; De Pouplana et al. 2001) did not show a similar root position for some ARSs, probably because the trees included fewer taxonomical sequences. The composite tree of class IIa and class IIb/c by Andam and Gogarten (2011) showed that the root positions of each branch of ProRS, ThrRS, and SerRS only are between Bacteria and Archaea. In our trees, the root position of GlyRS-1 was in the archaeal group. This is probably related to the fact that GlyRS-2 is used in Bacteria and plastids instead of GlyRS-1. The root position of LysRS was between the Bacteria and Archaea groups in the ML tree from alignment A, whereas it is in the bacterial group in the BI tree and the ML tree from alignment B.

### Amino Acid Specificity of Contemporary Class IIa and Class IIb ARS

The three-dimensional structures of the ARSs of contemporary organisms have been reported from crystal structure analysis. The amino acid residues interacting with cognate amino acid molecules have been assigned in some extant class II ARSs. In class II ARSs, two arginine residues are absolutely conserved, one in motif 2 and the other in motif 3 (Kaiser et al. 2018). One arginine residue in motif 2 binds the carboxyl group of the substrate amino acid and α-phosphate of ATP. The other arginine residue in motif 3 binds the adenine and γ-phosphate of ATP. Because these two arginine residues play an important role in aminoacylation, they have been termed “Arginine Tweezers” (Kaiser et al. 2018). However, they may not be relevant to the selection of substrate amino acid.

To understand the evolution of specificity of an amino acid in an ARS, we focused on the residues binding to the side chain or amino group of the substrate amino acid in the ARS. In the ARS, a zinc (Zn) ion is also involved in interacting with the cognate amino acid, and is coordinated by amino acid residues in the catalytic core of the ARS. The residues interacting with the substrate amino acid and the Zn ion have been described in reports of crystal structures of ARSs (Belrhali et al. 1995; Åberg et al. 1997; Arnez et al. 1997, 1999; Schmitt et al. 1998; Eiler et al. 1999; Sankaranarayanan et al. 2000; Onesti et al. 2000; Crepin et al. 2006; Bilokapic et al. 2006). We have summarized the amino acid residues interacting with the target group of the substrate amino acid in ARSs in Table 1 and Supplemental Table S4. The amino acid residues interacting with substrates in crystal structures are shown in Supplemental Figures S2A‒H.

The amino acid specificity of each contemporary ARS has been estimated by evaluating the conservation of amino acid residues interacting with substrate amino acids (Table 2). Amino acid residues at the substrate binding site of an ARS were compared with the corresponding residues of another ARS. We define an ARS as having amino acid specificity when it possesses amino acid residues interacting with a side chain or amino group of substrate amino acid, and a Zn ion binding to the side chain of a substrate amino acid. For example, *Thermococcus kodakarensis* AspRS has glycine at position 171. This is the same amino acid type found in the equivalent position in *E. coli* LysRS, which has five substrate binding residues. Accordingly, the PAAS of *T. kodakaraensis* AspRS to lysine is estimated to be 0.2. The evaluated PAAS of contemporary ARSs is higher than 0.8 with the cognate amino acid. Some ARSs can interact with noncognate amino acids, as reviewed by Tawfik and Gruic-Sovulj (2020); some of them are noted below.

SerRS of *T. thermophilus* has all the amino acid binding sites for proline and three of the five amino acid binding sites of lysine (Table 2). While mis-activation of noncognate amino acid by bacterial SerRS has not been reported, the SerRS of the methanogen, *Methanosarcina barkeri,* has been reported to mis-activate threonine weakly (Bilokapic et al. 2006). Although *M. barkeri* SerRS has only one of the five threonine binding sites, it has Zn ion binding sites (Table 1) which may be able to bind threonine.

LysRS of *E. coli* has amino acid binding sites for proline and serine. Mis-acylation of *E. coli* LysRS has been reported (Jakubowski 1999). This work showed that LysRS could activate arginine, threonine, methionine, leucine, alanine, serine, and cysteine. The result agrees with the partial possession of a serine binding site in *E. coli* LysRS, although no threonine binding site was found (Tables 1 and 2).

ThrRS of *M. jannaschii* has three of the five archaeal serine binding sites in addition to threonine binding sites (Tables 1 and 2). This is consistent with a previous study that showed that ThrRS of the methanogen *Methanosarcina mazei* mis-activated serine (Beebe et al. 2004). ThrRS of *E. coli* can bind serine, and a Zn ion in the active site prevents the binding of valine (Sankaranarayanan et al. 2000). Although ThrRS of *E. coli* has only one of the five serine binding sites, the bound Zn ion may be able to contribute to binding serine (Table 1).

ProRSs always have some serine binding sites (Table 1). In general, ProRS sometimes mis-activates alanine or cysteine, but the editing domain of ProRS hydrolyzes any mischarged tRNA to maintain translational accuracy (Beuning and Musier-Forsyth 2000, 2001). This implies that ProRS has the potential to bind noncognate amino acid. Furthermore, experimental evidence for the de-acylation mechanism of Ser-tRNA^Pro^ has been reported, showing that ProRS can mis-activate serine (Kumar et al. 2012). These studies support our estimation of the broad amino acid specificity of ProRS (Table 2).

Although GlyRS-1 of *T. thermophilus* has three of the five histidine binding sites (Tables 1 and 2), no experimental evidence for mis-activation of noncognate amino acid has yet been reported. In summary, some of the mischarging experimental results are compatible with the PAAS estimated from the substrate amino acid binding residues (Table 2, bolded and underlined), although experimental results are largely missing. More results of the mis-activation experiment will be needed in future, to confirm the correlation between the substrate recognition residue and the mis-activation of noncognate amino acid for ARSs, including the other class and the subclasses of ARSs.

### Amino Acid Specificity of Ancestral Class IIa and Class IIb ARSs

Ancestral sequences were predicted of the nodes of *C. commonote* ARS, ComD, ComK, ComH, ComG, ComT, ComP, and ComS, and of five ARS nodes, AncDK, AncHG, AncHGSPT, AncSPT, and AncSP (Supplemental Table S5). Amino acid residues at positions relevant to substrate amino acid recognition were selected from the ancestral ARS sequence, and the posterior probability of each amino acid residue was estimated. The four highest posterior probabilities for the amino acid in the position of substrate recognition residues in ancestral ARSs are listed in Supplemental Table S5. In the ML tree from alignment A, sequences were predicted at the nodes of *C. commonote* for ARSs, ComD, ComK, ComH, ComG, ComT, ComP, and ComS. The sequences at the node of the common ancestor of ARSs, AncDK, AncHGSPT, AncHG, AncSPT, and AncSP were also predicted in the ML tree from alignment A. In the Bayesian tree from alignment A and the ML tree from alignment B, sequences at 11 ancestral nodes were predicted: ComK, ComH, ComG, ComT, ComP, ComS, AncDK, AncHGSPT, AncHSPT, AncSPT, and AncSP. In the three different trees, ComD and AncHG are unique to the ML tree from alignment A, and AncHSPT is unique to the Bayesian tree from alignment A and the ML tree from alignment B.

For these *C. commonote* and ancestral ARS nodes in each tree, the amino acid specificities of ancestral ARS against seven substrate amino acids were estimated by averaging the posterior probability of each amino acid residue that matches with the substrate recognition residues (APP-SRR) (Table 3).

ComD in the ML tree showed the highest APP-SRR for aspartate in all analyses (Table 3). ComK and ComH showed the highest APP-SRR for lysine and histidine, respectively, in all analyses in three different trees. ComK also showed medium APP-SRR for proline and serine. The tendency is similar in the PAAS of contemporary LysRS (Table 2). ComG showed the highest APP-SRR for glycine, with high or medium APP-SRR for histidine in all the analyses. The tendency is similar in the PAAS of contemporary GlyRS-1 (Table 2). ComT showed the highest APP-SRR for threonine in all analyses, with medium APP-SRR for serine in seven analyses, which is similar to contemporary ThrRS. ComP showed the highest APP-SRR for proline, with high or medium APP-SRR for serine and medium APP-SRR for threonine. The tendency is similar in contemporary ProRS. ComS showed medium or high APP-SRR for serine in all analyses, with medium or high APP-SRR for proline in 11 analyses. The tendency is similar in contemporary bacterial SerRS. The substrate specificity thus estimated for ancestral ARS corresponding to the node of *C. commonote* is, in general, similar to the counterpart in contemporary ARS.

In the Bayesian tree and the ML tree from alignment B, LysRS diverged from AspRS (Figs. 3 and 4) and AncDK corresponds to ComD in the ML tree from alignment A. AncDK in the Bayesian tree and the ML tree from alignment B showed high APP-SRR for aspartate in all 10 analyses (Table 3). AncDK in the ML tree from alignment A showed medium or high APP-SRR for lysine, while medium or high APP-SRR for aspartate was also noted in three analyses (Table 3). Summarizing these results, the amino acid specificity of AncDK was for aspartate.

AncHGSPT showed high APP-SRR for histidine in all 15 analyses (Table 3). AncHGSPT also showed medium APP-SRR for glycine in seven analyses. These results suggest that AncHGSPT had high specificity for histidine, although glycine may also have been recognized.

AncHG, which is present only in the ML tree from alignment A, had high APP-SRR for histidine in all five analyses and showed an APP-SRR higher than 0.5 for glycine in IQ-TREE and RAxML analyses (Table 3). AncHSPT, which is present only in the Bayesian tree and the ML tree from alignment B, showed high APP-SRR for histidine in eight analyses (Table 3). AncHSPT also showed medium APP-SRR for glycine in three analyses.

AncSPT showed high or medium APP-SRR for threonine in all analyses and medium APP-SRR for serine in seven analyses. Medium APP-SRR for histidine was noted for AncSPT in two analyses from alignment B, with low specificity for serine. AncSPT showed specificity for threonine. AncSP was the most complicated, showing relatively high APP-SRR for threonine, proline, and serine, but with differing tendencies. Thus, AncSP was able to bind those three amino acids with weak specificity.

## Discussion

### Evolutionary Divergence in Class IIa and Class IIb ARSs

A composite tree of class II ARSs was reported by Nagel and Doolittle (1991, 1995). It suggested that ThrRS, ProRS, and SerRS formed a monophyletic branch, with a close relationship between ThrRS and ProRS. GlyRS-1 had not been discovered at that time. Their analyses also demonstrated that HisRS was close to AlaRS, and that AspRS and LysRS also formed a monophyletic branch. Härtlein and Cusack reported a composite tree based on the amino acid sequence alignment of 140 residues (1995). Their tree showed a similar relationship between ThrRS, ProRS, and SerRS to that reported by Nagel and Doolittle (1991, 1995). Additionally, GlyRS-1 was included and was closer to SerRS than to ProRS and ThrRS in their tree. HisRS and AlaRS also formed a monophyletic branch (Härtlein and Cusack 1995). De Pouplana et al. also reported a composite tree of class IIa ARSs rooted with AspRS (2001). This tree also showed that ThrRS, ProRS, and SerRS were monophyletic, and in particular showed that ThrRS and ProRS were more closely related. Of the class IIa ARSs, HisRS diverged earliest, followed by GlyRS-1, SerRS, ThrRS, and ProRS, in that order. Andam and Gogarten (2011) reported a composite tree of class IIa ARSs (ThrRS, ProRS, and SerRS) and class IIb/c ARSs (AspRS, AsnRS, LysRS, and PheRS). This composite tree also showed that ThrRS, ProRS, and SerRS were monophyletic and that ThrRS and ProRS were more closely related. LysRS was diverged from AspRS, which indicates that LysRS originated from AspRS after divergence between Archaea and Bacteria.

O’Donoghue and Luthey-Schulten proposed a dendrogram of class II ARSs based on similarities between the crystal structures of ARSs (2003). HisRS, GlyRS-1, ThrRS, ProRS, and SerRS were classified into the same structural cluster (class IIa) in this dendrogram, while AspRS and LysRS were classified into class IIb. Additionally, a RMSD cluster dendrogram based on 80 residues in the conserved core of class II ARSs supported the classification of these ARS into class IIa and class IIb (Valencia-Sánchez et al. 2016).

The conclusion in previous analyses, that ThrRS, ProRS, and SerRS are monophyletic, is supported in our analysis. However, in previous analysis ThrRS and ProRS were closer and formed a monophyletic branch, whereas in our results SerRS and ProRS are closest. Our detailed sequence alignment and a more complex evolutionary model of phylogenetic analysis indicates a closer relationship between SerRS and ProRS based on strong statistical support, compared with the monophyletic ThrRS and ProRS relationship proposed in previous analyses (Härtlein and Cusack 1995; De Pouplana et al. 2001; Andam and Gogarten 2011). Part of the amino acid binding sites for both Pro and Ser are conserved in ProRS and bacterial SerRS (Table 1), which also supports the notion of a monophyletic branch consisting of SerRS and ProRS.

As for the evolutionary relationship between HisRS and GlyRS-1, our composite tree produced by ML analysis from alignment A suggests that HisRS and GlyRS-1 constitute a monophyletic branch. This is consistent with some dendrograms reported previously by other groups (O’Donoghue and Luthey-Schulten 2003; Valencia-Sánchez et al. 2016). In contrast, in our Bayesian tree and ML tree from alignment B, GlyRS-1 is demonstrated to have diverged earlier, with HisRS diverging second in the evolutionary tree of class IIa ARSs. The existence of a monophyletic branch containing GlyRS-1/HisRS is supported with low statistical confidence in the ML tree from alignment A, and the monophyletic branch of HisRS and three other ARSs (ThrRS, ProRS, and SerRS) is supported with medium statistical confidence in the Bayesian tree and the ML tree from alignment B. Accordingly, our study supports GlyRS-1 diverging earlier.

Comparison of the composite trees reveals different results for the evolutionary relationship between AspRS and LysRS. In the ML tree from alignment A, the monophyletic branches of AspRS and LysRS showed sisterhood. But in the Bayesian tree and the ML tree from alignment B, LysRS diverged from archaeal AspRS with low posterior probability and medium rbp. Our trees tend to support the latter case: that LysRS derived from archaeal AspRS.

Some lateral gene transfers in each of the ARS gene trees were noted and reported by Furukawa et al. (2017). In this study, the root of SerRS was found between the methanogenic SerRS and bacterial SerRS branch, including some archaeal SerRSs. It is likely that *C. commonote* had SerRS, which diverged into archaeal and bacterial SerRS. After divergence between Archaea and Bacteria, most Archaea accepted the bacterial SerRS gene in the early stage of archaeal evolution, with the archaeal SerRS gene subsequently being lost in most Archaea.

### Domain Evolution in Class IIa ARS

**Fig. 5:**
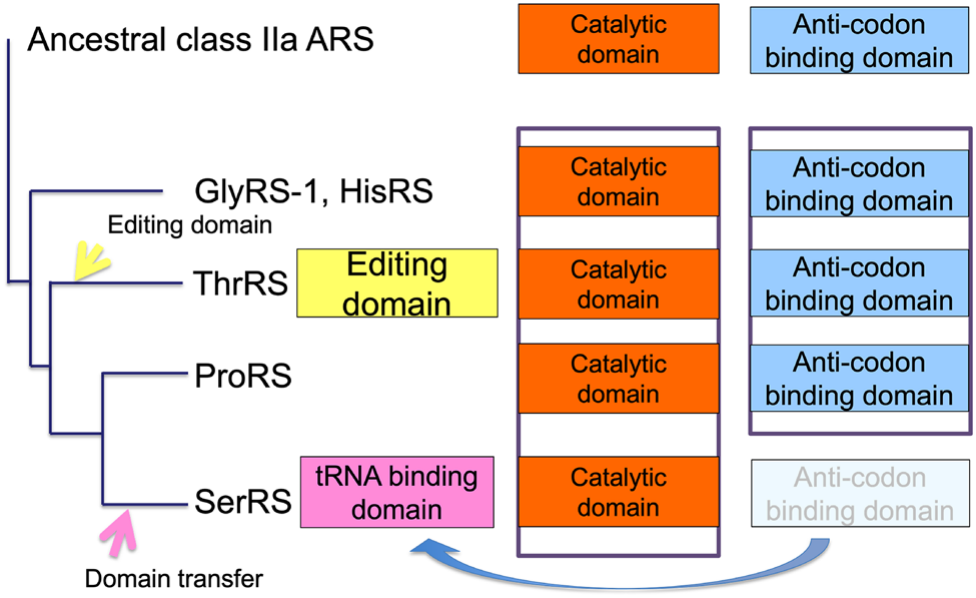
Domain evolution in class IIa ARS. From the ancestral class IIa ARSs, consisting of a catalytic domain and anticodon binding domain, class IIa ARSs diverged. An editing domain was added to the N-terminus during the evolution of ThrRS. The anticodon binding domain was transferred from the C-terminal end to the N-terminal end forming the tRNA binding domain during the evolution of SerRS

Based on alignment of class IIa ARSs (Supplemental Table S3) and the phylogenetic tree of class IIa ARSs (Supplemental Fig. S1), we propose a new evolutionary model for class IIa ARS structural domains (Fig. 5). The catalytic domain is always conserved in crystal structures of class IIa ARSs. The anticodon binding domain is also conserved in GlyRS-1, HisRS, ThrRS, and ProRS. An editing domain has been identified at the N-terminal side of ThrRS, and a tRNA binding domain has been identified at the N-terminal side of SerRS.

Despite efforts to align, we could find no similarity in secondary structural topology between the N-terminal editing domain of ThrRS and the N-terminal tRNA binding domain of SerRS (Supplemental Table S3a). In contrast, we found similarity in the secondary structural topology between the tRNA binding domain of archaeal SerRS and the anticodon binding domain of HisRS, GlyRS-1, ThrRS, and ProRS (Supplemental Table S3b). This suggests the transfer of the anticodon domain from the C-terminus to the N-terminus, resulting in the formation of the tRNA binding domain in SerRS (Fig. 5).

A phylogenetic tree was constructed from the sequence in which the tRNA binding domain of SerRS was moved from the N-terminal to the C-terminal side, and the phylogenetic tree showed the same topology as that in the seven ARSs composite tree, with the same SerRS position (Supplemental Fig. S1). In particular, the topology in the crystal structure of the tRNA binding domain of archaeal SerRS is similar to the topology of the anticodon binding domain of four ARSs (HisRS, GlyRS-1, ThrRS, and ProRS; Supplemental Table S3b). The tRNA binding domain of contemporary SerRS binds to the variable arm of tRNA^Ser^ and does not recognize the anticodon of tRNA^Ser^ (Biou et al. 1994). The domain transfer of the anticodon binding domain might be related to a need for flexibility in tRNA binding by SerRS, so as to accept tRNA^Ser^ with anticodons corresponding to six serine codons.

### Ancestral Amino Acid Specificity of *C. commonote* ARSs

By comparing the substrate binding residues with those of the contemporary ARS, the amino acid specificities of *C. commonote* ARSs were predicted. ComD, ComH, and ComT were predicted to have specificity only for the respective cognate amino acid (Table 3). ComT might mis-activate serine, similar to contemporary ThrRS. However, the N-terminal editing domain in ComT is likely to edit mis-acylated noncognate amino acid. ComK was predicted to have specificity for lysine and possible binding activity for proline and serine, which is partially consistent with the mis-acylation observed with extant LysRS, which can activate arginine, threonine, methionine, leucine, alanine, serine, and cysteine (Jakubowski 1999). ComG was predicted to have specificity for glycine and histidine. ComP was predicted to have specificity for proline and serine, and possible binding activity for threonine. Contemporary ProRS has an editing domain that prevents mis-acylation. However, the position and sequence length of the editing domain differ in Archaea and Bacteria (Yaremchuk et al. 2000), which suggests that ComP had no editing domain in *C. commonote*. The editing domain may have been added after the divergence of Archaea and Bacteria. Accordingly, ComP might have ambiguous amino acid specificity. ComS was predicted to have specificity for serine and possible binding activity for proline. To summarize the amino acid specificities for *C. commonote* ARSs, each *C. commonote* ARS is predicted to have specificity for its cognate amino acid, with possible mis-activation and mis-acylation activities, as illustrated by the specificities of ComK, ComG, ComT, ComP, and ComS. However, this relaxed specificity is also found in many extant ARSs. Accordingly, the accuracy of the translation system of *C. commonote* was probably similar to that of contemporary organisms, although there may have been some ambiguity.

### Amino Acid Specificity of Ancestral Translation System Before *C. commonote*

**Fig. 6:**
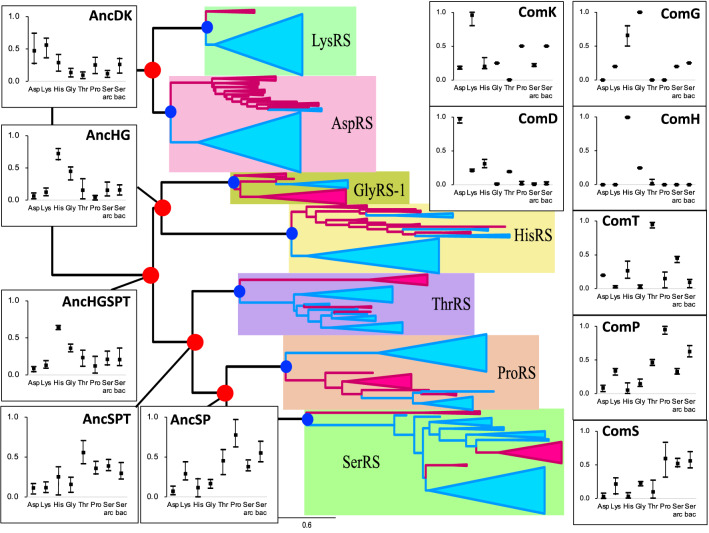
Amino acid specificity of ancestral ARSs in the ML tree from alignment A. The range and average of APP-SRR for the specific amino acid of the ancestor at the node estimated in Table 3 is indicated at each node of the ML tree. AncX and ComX were pointed in red and blue, respectively

**Fig. 7:**
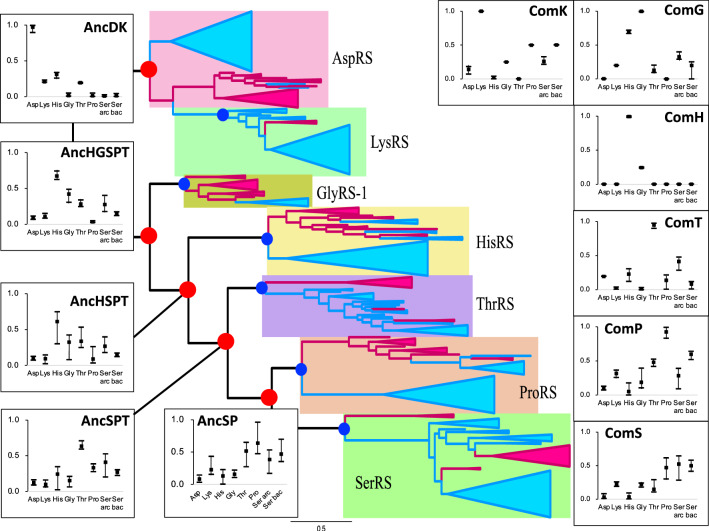
Amino acid specificity of ancestral ARSs in the Bayesian tree from alignment A. The range and average of APP-SRR for specific amino acids of the ancestor at the node estimated in Table 3 are indicated at each node of the Bayesian tree. AncX and ComX are indicated in red and blue, respectively

**Fig. 8:**
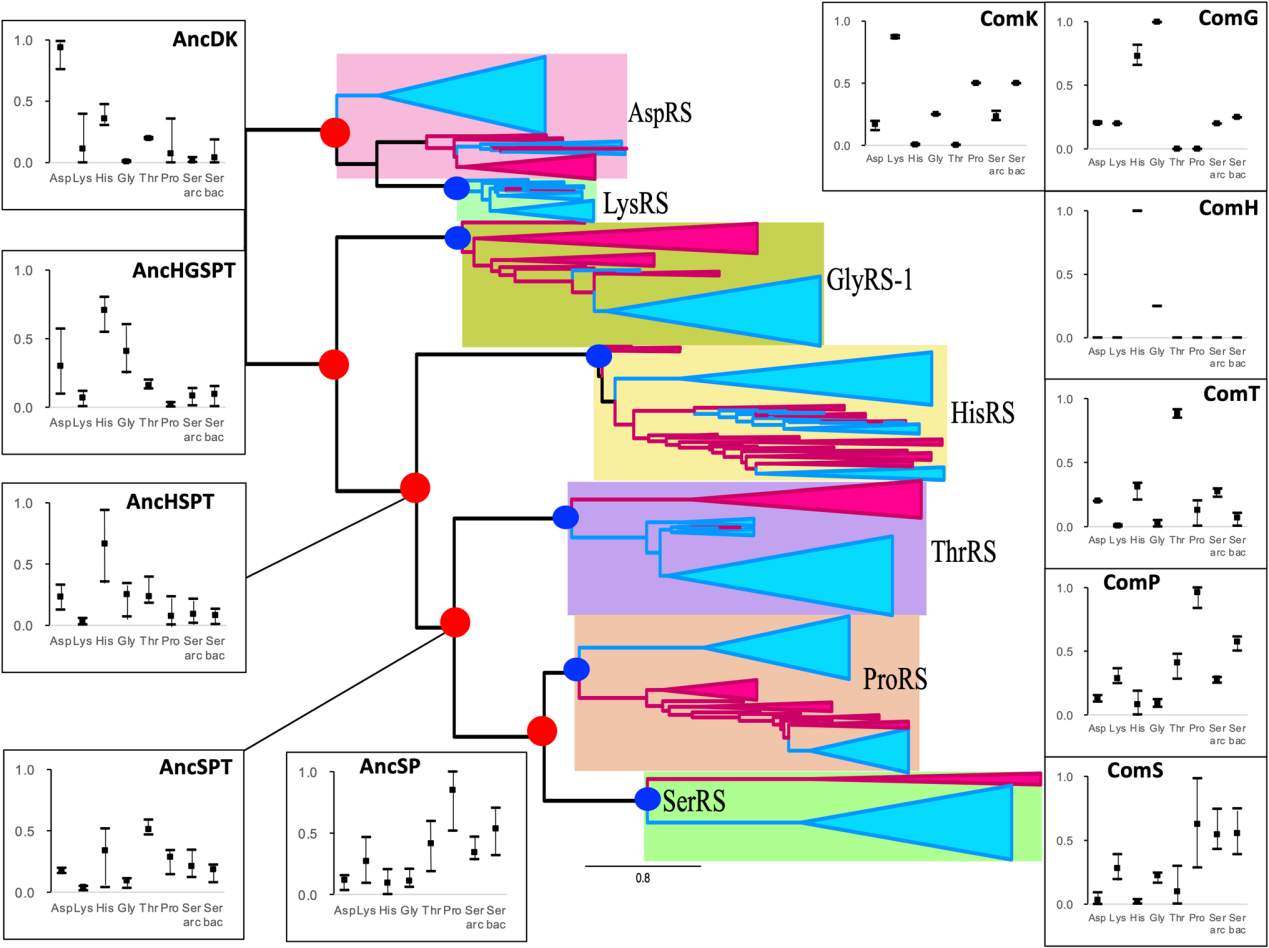
Amino acid specificity of ancestral ARSs in the ML tree from alignment B. The range and average of APP-SRR for specific amino acids of the ancestor at the node estimated in Table 3 are indicated at each node of the ML tree. AncX and ComX are indicated in red and blue, respectively

The amino acid specificity of ancestral ARSs prior to *C. commonote* was predicted from the sequences of six ancestral ARSs: AncDK, AncHGSPT, AncHG, AncHSPT, AncSPT, and AncSP (Table 3, Figs. 6, 7 and 8). The deepest nodes in this study are AncHGSPT and AncDK, which correspond to the ancestors of class IIa ARSs and class IIb ARSs, respectively. Several methods using three trees were employed to study the ancestral sequences. In all analyses with three trees, AncHGSPT showed binding specificity for histidine (Table 3, Figs. 6, 7 and 8).

In general, the substrate specificity of ancestral proteins is considered to be broad, and these characteristics are termed promiscuous (reviewed in Siddiq et al. 2017). If the ancestral ARSs had broad specificity and randomly activated any amino acid, primitive proteins or peptides would be disordered, with very low catalytic efficiency. However, AncHGSPT was predicted to have specificity for histidine, with its specificities for glycine, threonine, proline, and serine being much lower (Figs. 6, 7 and 8). After gene duplication, AncHGSPT evolved to AncHG, AncSPT, and AncHSPT. AncHG and AncHSPT have specificity for histidine and possible weak specificity for glycine or threonine.

AncSPT had specificity for threonine and possibly serine, having lost specificity for histidine. In most analyses, threonine binding sites were conserved (Table 3, Supplemental Table S5). This suggests that AncSPT may have had a Zn ion involved in specific binding of both threonine and serine (Table 1, column numbers 546, 605, 607, and 1362). Because a Zn ion directly binds threonine in extant ThrRS (Sankaranarayanan et al. 2000) and serine in extant archaeal type SerRS (Beebe et al. 2004), a Zn ion in AncSPT is predicted to bind both threonine and serine. The fact that extant ThrRS mis-activates serine (Sankaranarayanan et al. 2000) supports the prediction. Proline binding sites and bacterial type serine binding sites were absent in most analyses, which suggests that AncSPT did not bind proline (Table 3, Supplemental Table S5).

AncSP was predicted to have multiple specificities, depending on the presence of Zn ion binding sites. In about half of the analyses, threonine binding sites were conserved (Table 3, Supplemental Table S5), which suggests that AncSP had a Zn ion and could also recognize serine (Table 1, column numbers 546, 605, 607, and 1362). Proline binding sites were conserved in most analyses (Table 3). Bacterial type serine binding sites were conserved in about half of the analyses. To summarize these predictions, AncSP is predicted to bind threonine, serine, and proline, which suggests that AncSP was promiscuous for these three amino acids (Figs. 6, 7 and 8).

AncDK was predicted to have a specificity to aspartate. In predictions based on the ML tree, AncDK had specificity for lysine in four analyses, and specificity for aspartate in three analyses based on alignment A. AncDK from RAxML analysis showed specificity for both lysine and aspartate. AncDK based on alignment B showed specificity for aspartate. These results suggest that AncDK tended to bind aspartate but there was a possibility of binding lysine. In estimations based on the Bayesian tree, AncDK corresponds to ComD, that had only an aspartate binding mode. In the Bayesian tree, LysRS originated from AspRS, suggesting that lysine is a late addition to the protein ARS system. As an alternative way of activating lysine, it is possible that there was a ribozyme ARS involved, as discussed later.

### Model of Evolution of Translation System Before *C. commonote*

Several publications have tested the idea of reducing the number of amino acid species while maintaining proteinaceous structure and function. They reported that about 10 amino acid species are required to obtain a proteinaceous structure with function (reviewed in Longo and Blaber 2012; Longo et al. 2013; Shibue et al. 2018; Kimura and Akanuma 2020). Our prediction of the ancestral ARS sequence suggests that a limited number of amino acid species may have been used in a translation system prior to *C. commonote*. If we assume that approximately 10 amino acid species are needed to produce active ARS, then 10 ARSs must have been present at the beginning of the proteinaceous ARS-dependent translation system, in order for the system to emerge. Or the initial translation system might have used ARSs consisting of RNA, namely ribozyme ARSs, with the proteinaceous ARSs appearing later and gradually replacing the ribozyme ARSs.

Phylogenetic analyses of the Rossmann fold superfamily have suggested that the class I ARS ancestor diverged from nucleotide-binding proteins (Aravind et al. 2002). The class II ARS fold is also structurally similar to the biotin synthase superfamily, which suggests that the class II ARSs may be diverged from this superfamily (Artymiuk et al. 1994; Anantharaman et al. 2002). These hypotheses also imply that protein synthesis was performed without proteinaceous ARSs during the early stages of the development of the translation system (Härtlein and Cusack 1995). Wolf and Koonin suggested an RNA-molecule-only translation system, in which peptides were translated using ribozymes, specifically the proto-tRNAs (2007). The translation system using proteinaceous ARSs may therefore have appeared after the RNA-molecule-only translation system.

**Fig. 9:**
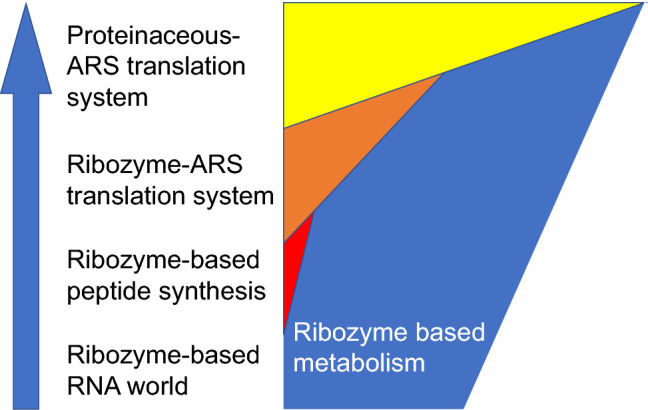
An evolutionary model for the proteinaceous translation system. The blue arrow indicates the direction of historical progress. In the RNA world, ribozymes were involved in all metabolic reactions in primitive cells surrounded by a cell membrane. Peptide synthesis started upon the emergence of peptidyl transferase, without using codons or tRNA. Ribozyme-ARSs became involved in the aminoacylation of tRNA upon the emergence of tRNA itself. Ribozyme-ARSs were gradually replaced by proteinaceous ARSs, after a sufficient number of amino acid species were incorporated into the translation system to produce efficient proteinaceous ARSs. After the proteinaceous ARSs replaced all the ribozyme-ARSs, the number of proteinaceous ARSs may have increased, resulting in an increased number of proteinaceous enzymes with increased catalytic efficiency. We are unsure when proteinaceous ARSs appeared; it may have been later, during a period when more amino acid species were used with ribozyme ARSs. The blue and red areas indicate the degree of metabolic reaction catalyzed by ribozyme and peptide, respectively. The orange and yellow areas indicate the degree of metabolic reaction catalyzed by proteinaceous enzymes synthesized by ribozyme-ARS and by proteinaceous ARS, respectively

We are able to hypothesize a model of the evolutionary history of the translation system before *C. commonote* (Fig. 9). In the RNA world, organisms were maintained by the metabolism catalyzed by ribozymes. Peptide synthesis started upon the development of peptidyl transferase. The first peptide may not have been encoded by an RNA sequence and may have been a random sequence (Bernhardt and Tate 2010). The system gradually shifted toward RNA encoded translation upon development of primitive tRNA and ribozyme ARSs, whose properties and number are unknown. The primitive translation system matured when the system incorporated more than 10 amino acid types using more than 10 ribozyme ARSs. (There is an alternative scheme in the model, in which fewer than 10 ribozyme ARSs were involved in the amino acylation of primitive tRNA, and the tRNA itself was responsible for selecting a cognate substrate amino acid.)

The two old ARSs, the ancestral class I and class II ARSs must then have appeared, to partially replace the role of ribozyme ARSs. If RNA-only translation had already evolved to achieve high fidelity prior to the involvement of protein-based ARS enzymes, then the protein-based ARSs would have needed higher specificity to compete with ribozyme ARSs. If specific charging was somehow difficult to achieve using ribozymes, the translation system might have evolved further to take advantage of the greater specificity afforded by protein-based ARSs. The ancestral class II ARS diverged into several subclass ARS ancestors. In this study we describe some inferences about the evolution of class IIa and class IIb ARSs.

Of the ancestral ARSs, AncHGSPT and AncDK appeared with specificity for a limited number of amino acids (Asp, Lys, and His). However, Lys and His are not included in the pre-biotic set of amino acids proposed by Longo and Blaber (2012), based on the meteorite information, spark experiments, and hydrothermal experiments. That set comprises: Ala, Asp, Glu, Gly, Ile, Leu, Pro, Ser, Thr, and Val. The presence of His is necessary for catalytic activity (Shibue et al. 2018; Kimura and Akanuma 2020). Accordingly, Lys and His might have been synthesized by a primitive metabolic system consisted of ribozyme and/or proteins consisting of pre-biotic amino acids.

Subsequently the ancestral organism used three proteinaceous ARSs (AncDK, AncHG, and AncSPT), recognizing lysine, aspartate, histidine, glycine, and threonine. Later, the ancestral organism started to use four proteinaceous ARSs (Asp, His, Thr, and Pro/Ser). Judging from the separation of archaeal and bacterial branches at the *C. commonote* node in each of AspRS, HisRS, ThrRS, ProRS, and SerRS, as shown in Figs. 2, 3 and 4, the last universal common ancestor *C. commonote* shifted to use ComD, ComH, ComT, ComP, and ComS (which are the class IIa and IIb ARSs). Although *C. commonote* may not have possessed LysRS-II or GlyRS-1, it probably possessed the other types of ARSs, LysRS-I and GlyRS-2. Taking into consideration all other classes and subclasses of ARSs, *C. commonote* synthesized protein via a translation system using ARSs for 20 standard amino acids. In this work we were able to obtain the partial history related to the evolution only of class IIa and class IIb ARSs. The ARSs in the other subclasses, IIc and IId, and in class I, are still to be analyzed to obtain the full picture of evolution from the early age of life to that of *C. commonote* having a full set of proteinaceous ARSs*.*

### Conclusion

In conclusion, our study suggests a domain-shifting event in the evolutionary history of class IIa ARSs. It predicts the specificity of ancestral ARSs by combining the ancestral sequence and the substrate recognition amino acid residues. The prediction suggests that a limited number of amino acids species were catalyzed by ancestral ARSs. In particular, AncHGSTP and AncDK, with narrow amino acid specificity, fill an information gap in evolutionary history, from an early translation system consisting of both ribozyme and proteinaceous ARSs to the present translation system performed by proteinaceous ARSs. However, our prediction requires experimental evidence for amino acid specificity of ancestral ARSs. To clarify the actual amino acid specificity of ancestral ARSs, we are planning biochemical experiments resurrecting ancestral proteinaceous ARSs, using the resurrection method we employed for the analysis of *C. commonote* (Akanuma et al. 2015).

In this work we analyzed the substrate amino acid specificity of ancient ARSs. The same approach can be applied to the tRNA binding domains of the synthetases, and the results can be correlated with the corresponding evolutionary trees for the tRNA isoacceptor species. Information on ancestral tRNA identity elements that evolved into modern identity elements of the synthetases will be obtained in the future.

There are many metabolites in cells. The pathways that consisted of certain metabolites would have evolved at different periods of evolution. It is unclear what kind of metabolite pool might have been present in *C. Commonote*, or organisms present at the nodes of the evolutionary tree. In addition to the efficient charging of one or a few standard alpha-amino acids to tRNAs, the ancestral ARSs had to be able to exclude a much larger set of unknown non-standard alpha-amino acids and metabolites from mis-activation. Geological and metabolic evolutionary events in the history of life might be obtained in future analysis, constructing such datasets using ancestral ARSs.

## Supplementary Information

Below is the link to the electronic supplementary material.

Supplementary file1 (DOCX 1680 kb)

Supplementary file2 (XLSX 56 kb)

Supplementary file3 (XLSX 1192 kb)
