# Supplementary material for: Amino Acid Specificity of Ancestral Aminoacyl-tRNA Synthetase Prior to the Last Universal Common Ancestor Commonote commonote
Source: J Mol Evol. 2022 Jan 27;90(1):73–94. doi: 10.1007/s00239-021-10043-z (PMC8821087; doi:10.1007/s00239-021-10043-z)
Supplement: Supplementary file 1 — Supplementary file1 (DOCX 1680 kb) [file 239_2021_10043_MOESM1_ESM.pdf]

Amino acid specificity of ancestral aminoacyl tRNA synthetase prior to the last universal common ancestor *Commonote commonote*

Ryutaro Furukawa [1,2], Shin-ichi Yokobori [1], Riku Sato [1], Taimu Kumagawa [1], Mizuho Nakagawa [1], Kazutaka Katoh [3,4] and Akihiko Yamagishi [1]

1. Department of Applied Life Sciences, School of Life Sciences, Tokyo University of Pharmacy and Life sciences, 1432-1 Horinouchi, Hachioji, Tokyo, Japan
2. Faculty of Human Science, Waseda University, 2-579-15 Mikajima, Tokorozawa, Saitama 359-1192, Japan
3. Department of Genome Informatics, Genome Information Research Center, Research Institute for Microbial Diseases, Osaka University, 3-1 Yamadaoka, Suita 565-0871, Japan
4. Systems Immunology Laboratory, Immunology Frontier Research Center, Osaka University, 3-1 Yamadaoka, Suita 565-0871, Japan

Corresponding author: Akihiko Yamagishi, E-mail: Yamagish@toyaku.ac.jp

Supplementary materials

Supplemental figure S1. Maximum likelihood composite tree of class IIa ARSs (HisRS, GlyRS-1, ThrRS, ProRS and SerRS). Archaeal branches and bacterial branches are colored magenta and blue, respectively. The scale bar indicates the number of substitutions per site. Numbers on each node indicate RELL bootstrap values. Log likelihood was -64323.7.

Supplemental figure S2. The structure showing binding between substrate amino acid and active sites in each ARS. The oxygen and nitrogen atoms are colored red and blue, respectively. Active site residues are colored orange. Zn and Zn binding site residues are colored marine blue.

A. The structure around the bound amino acid in *T. kodakaraensis* AspRS (Schmitt et al. 1998). AMO is aspartyl-adenosine-5'-monophosphate. S190, Q192, K195, D231, and R368 are active site residues in 3NEM.

B. The structure around the bound amino acid in *E. coli* LysRS (Onesti et al. 2000). LAD is adenosine-5'-[lysyl-phosphate]. G216, E240, E278, Y280, and E428 are active site residues in 1E1T.

C. The structure around the bound amino acid in *T. thermophilus* HisRS (Aberg et al. 1997). HSS is 5'-O-[(L-histidylamino)sulfonyl]adenosine. E83, T85, Q127, E131, Y263, and Y264 are active site residues in 2EL9.

D. The structure around the bound amino acid in *T. thermophilus* GlyRS (Arnez et al. 1999) GAP is glycyl-adenosine-5'-phosphate. E188, E239, E359, and S361 are active site residues in 1GGM.

E. The structure around the bound amino acid in *E. coli* ThrRS (Sankaranarayanan et al. 2000) X16 is [[3-(4-amino-2-methylquinazolin-7-yl)phenyl]sulfonyl]-L-threoninamide. D383 and Y462 are active site residues in 4HWP. C334, H385 and H511 are Zn binding site residues.

F. The structure around the bound amino acid in *T. thermophilus* ProRS (Crepin et al. 2006). P5A is 5'-O-(N-(L-prolyl)-sulfamoyl)adenosine. T117 and E119 are active site residues in 1NJ5.

G. The structure around the bound amino acid in *M. barkeri* SerRS (Bilokapic et al. 2006). SSA is 5'-O-(L-seryl)-sulfamoyl)adenosine. A304 and R353 are active site residues in 2CJ9. C306, E355, and C461 are Zn binding site residues.

H. The structure around the bound amino acid in *T. thermophilus* SerRS (Belrhali et al. 1995). T252, E254, E306, and T406 are active site residues in 2DQ0.

Supplemental figure S1

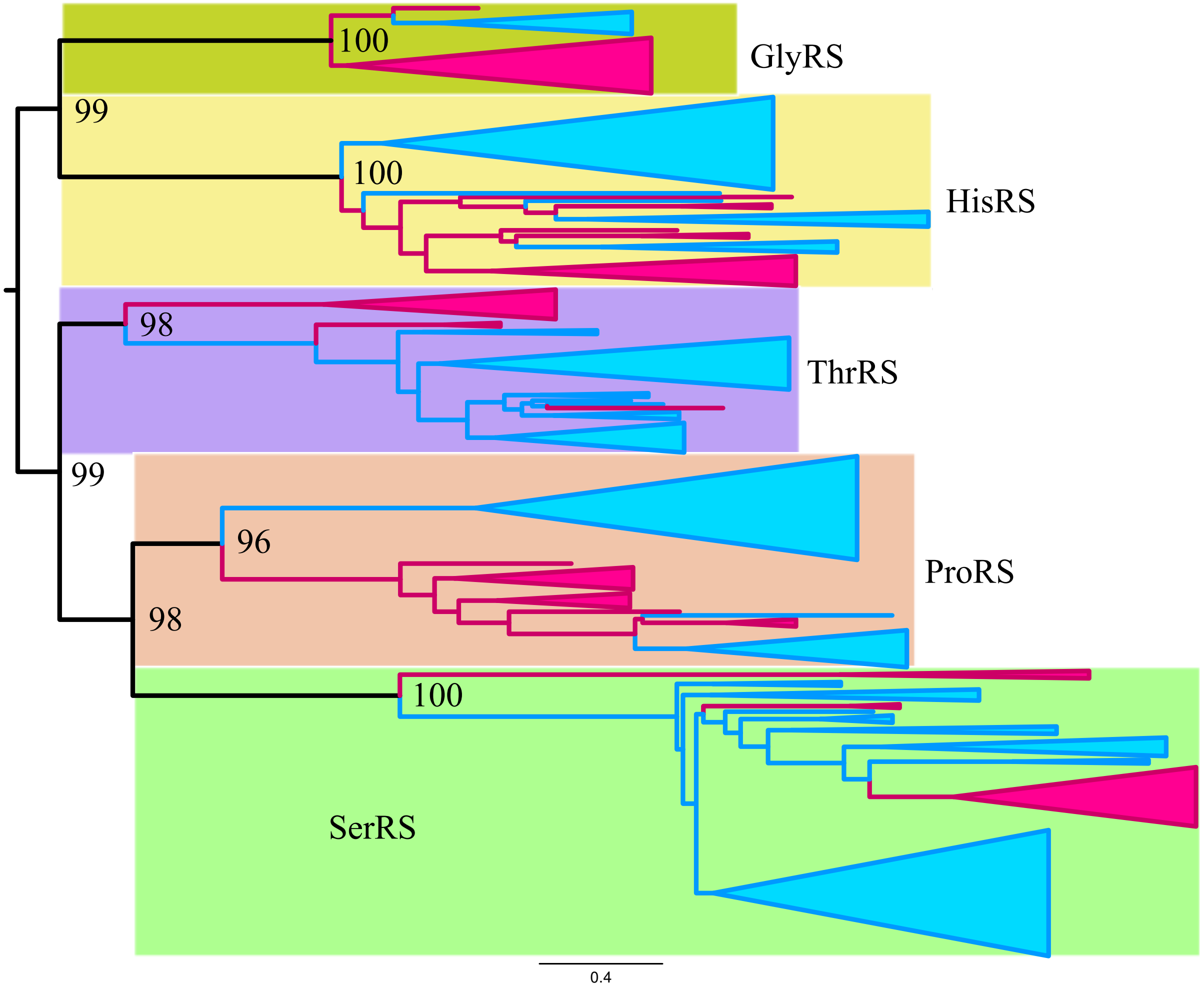

Supplemental figure S2

A.

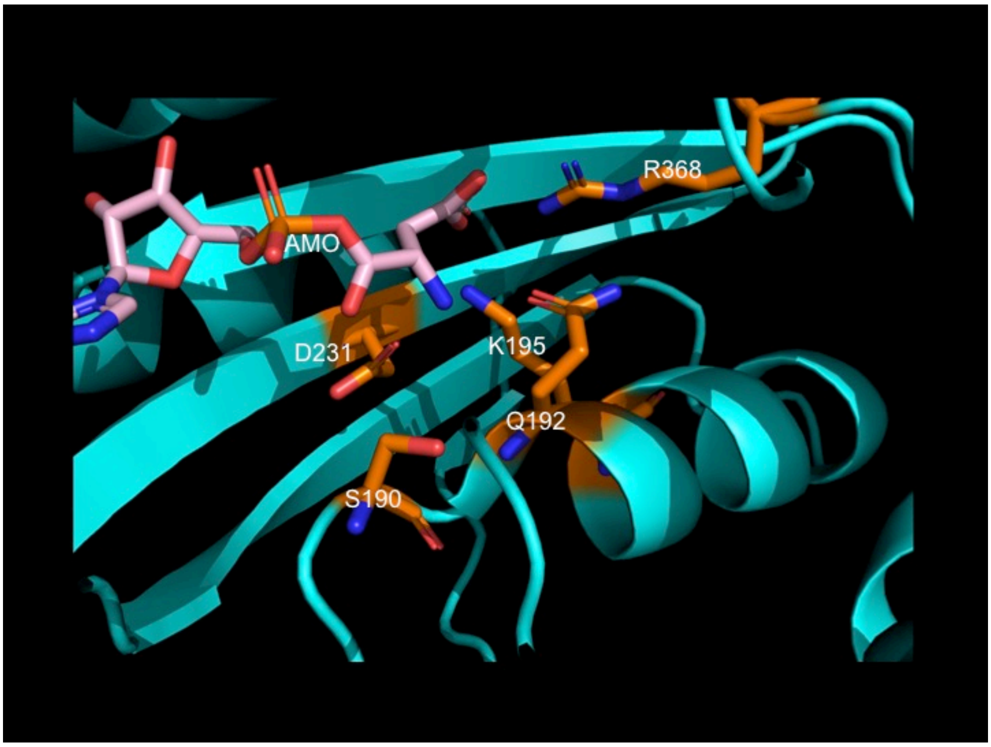

B.

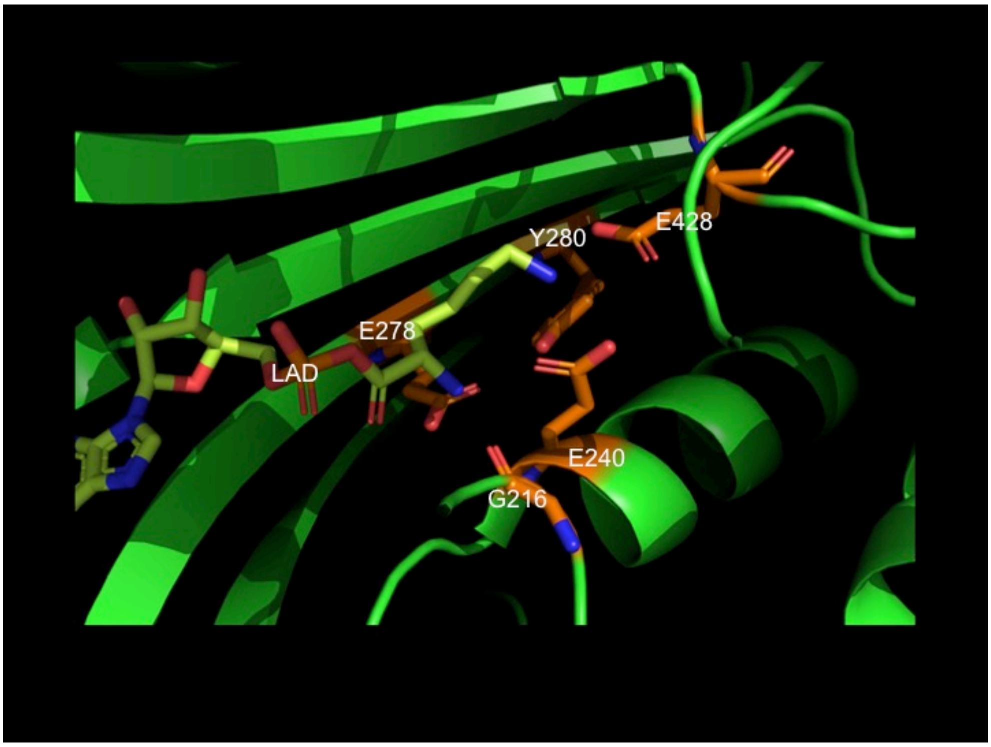

C.

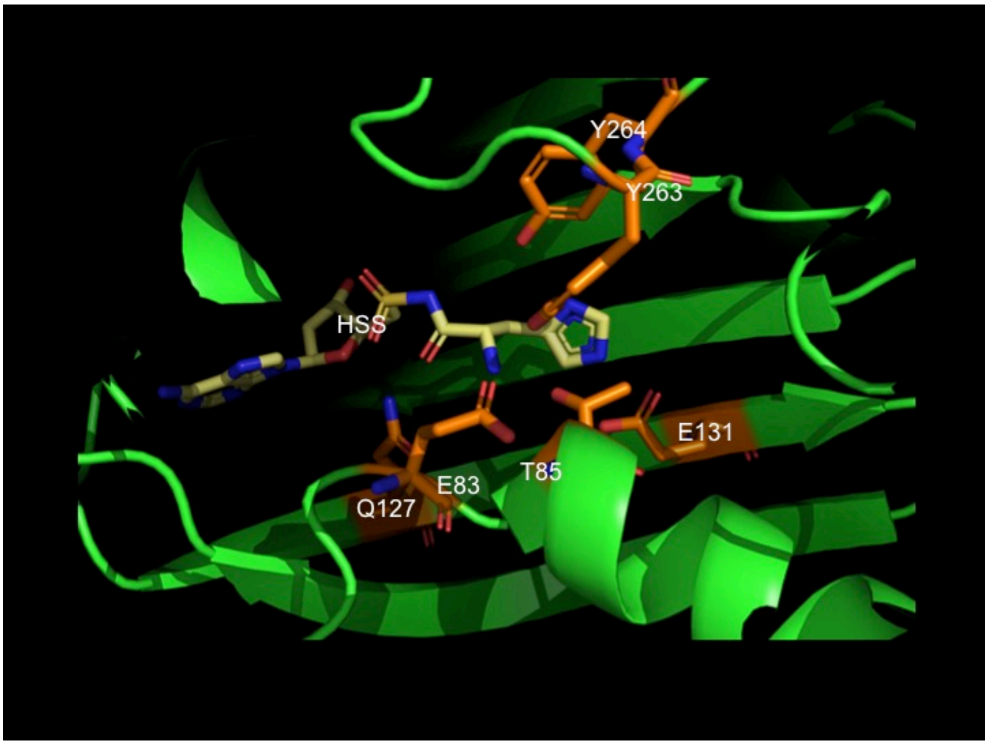

D.

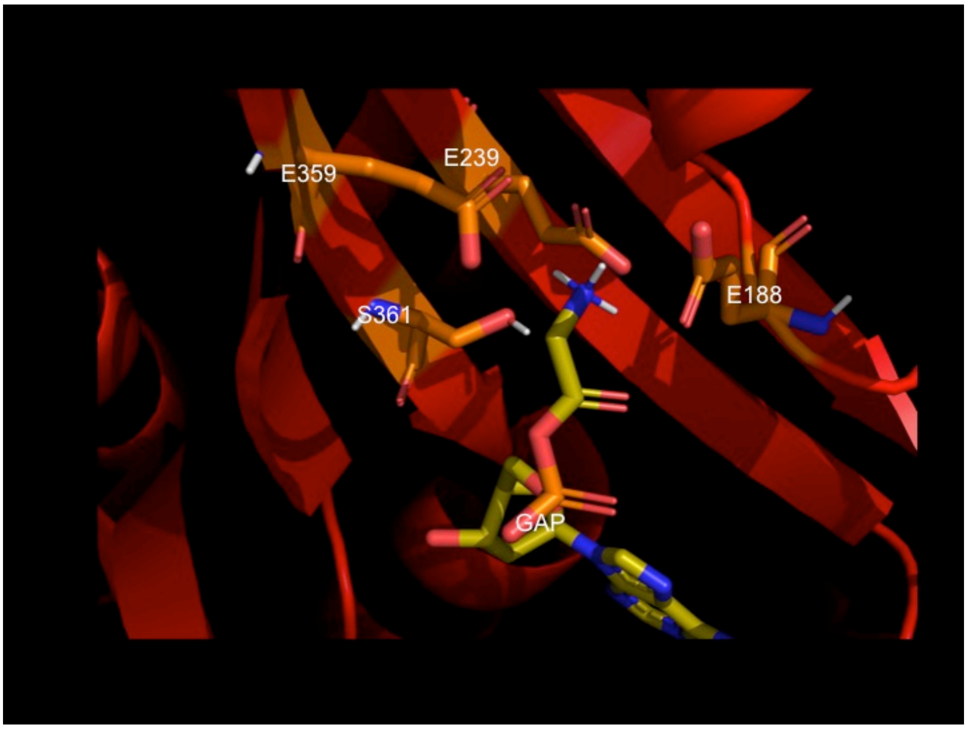

E.

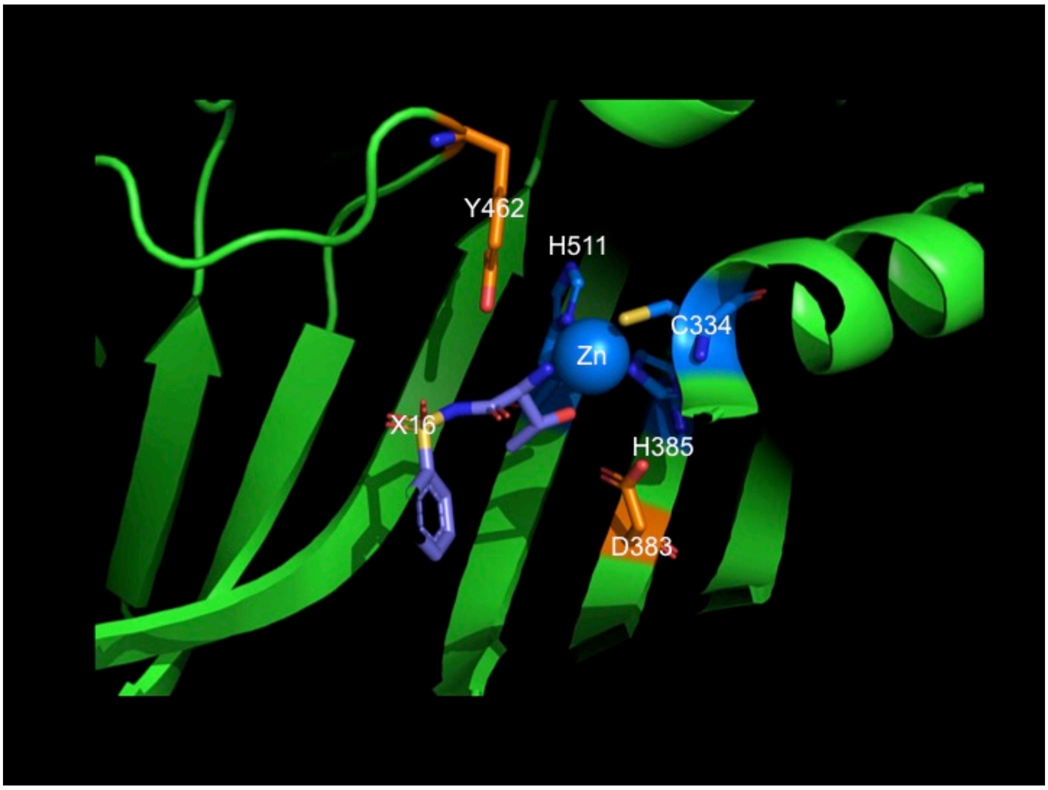

F.

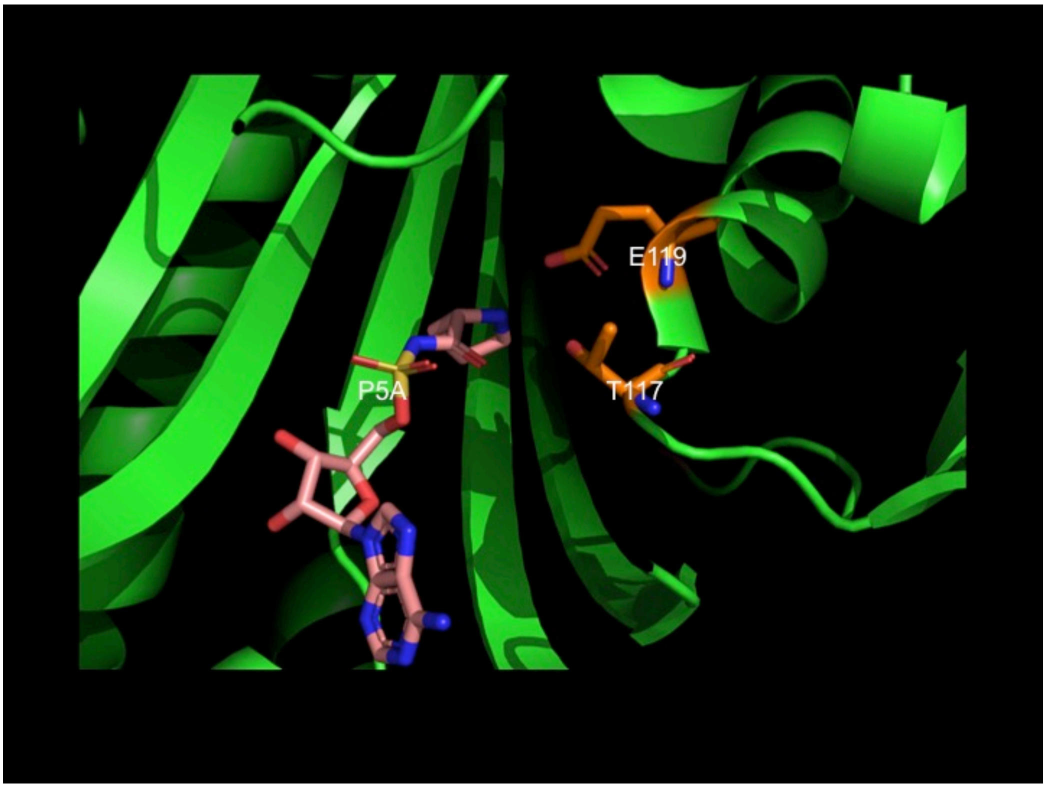

G.

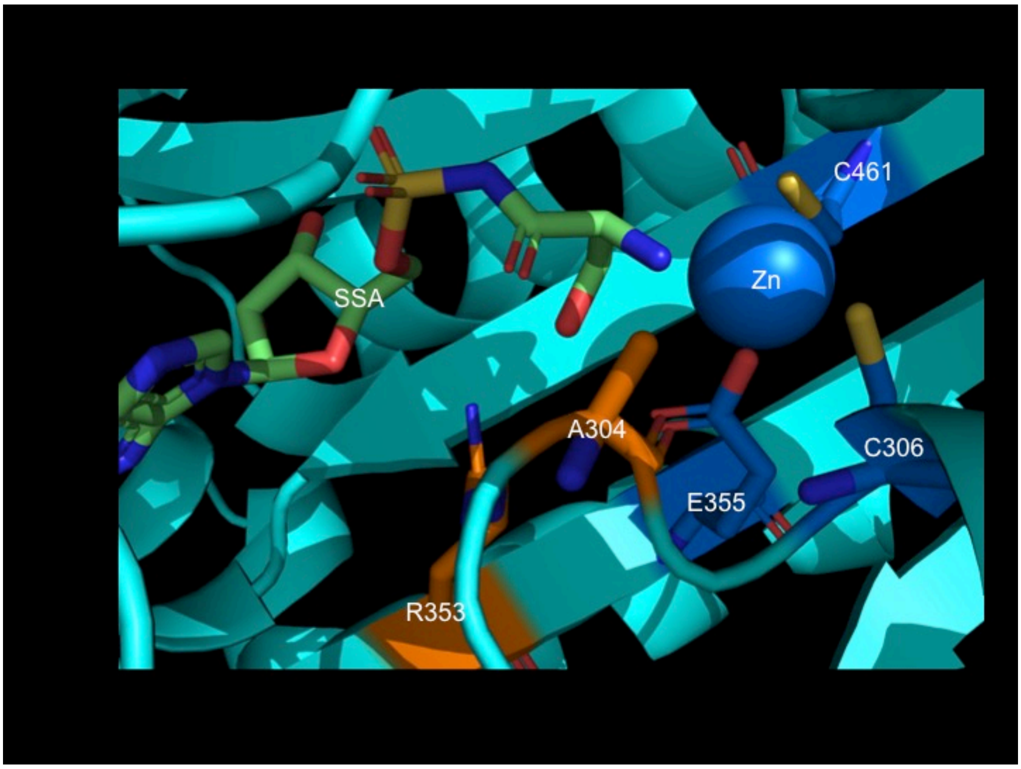

H.

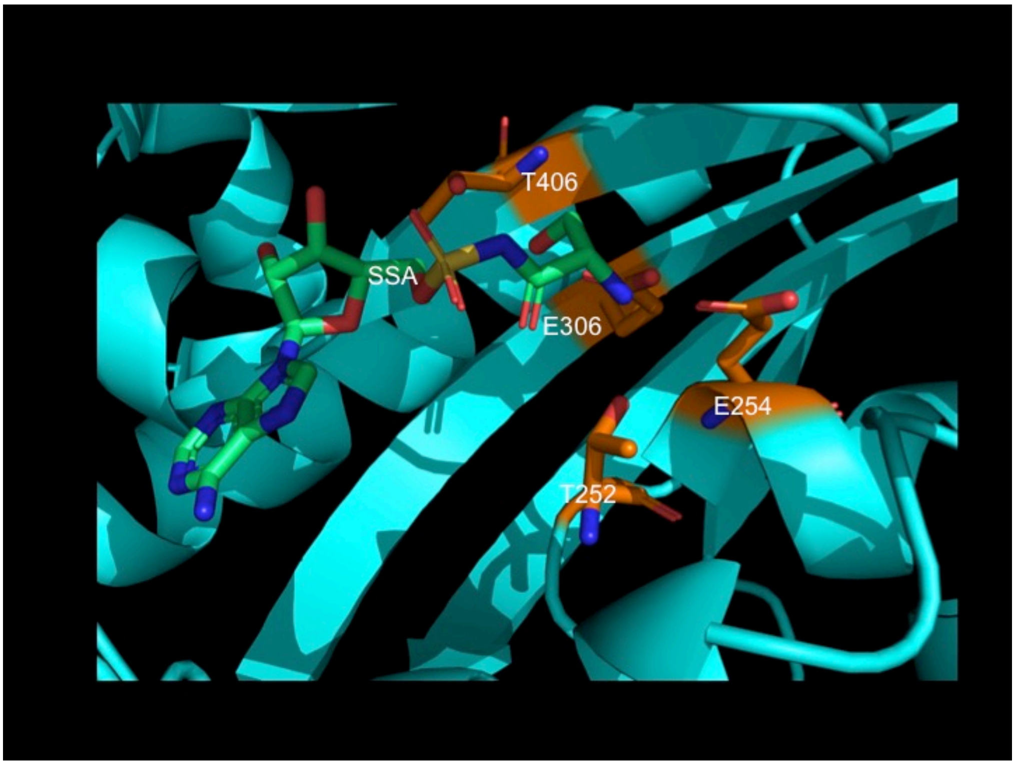

Supplemental table S1. Collected sequence list of seven aminoacyl tRNA synthetases used in alignment A. Numbers colored in red were excluded from final alignment.

Supplemetal table 1 Species List	AspRS	GlyRS-1	HIsRS	Class II-LysRS	ProRS	SerRS	SerRS-rare	ThrRS
Candidatus_Caldiarchaeum_subterraneum	BAJ50936	BAJ49678	BAJ47360		BAJ50117	BAJ49618		BAJ49105
Ignicoccus_hospitalis_KIN4/I	ABU82611.1	YP_001435157	YP_001435468.1		YP_001434952.1	YP_001435576.1		YP_001434903.1
Aeropyrum_pernix_K1	NP_148450	NP_148072	NP_147398		NP_148542	NP_148300		NP_147510
Sulfolobus_tokodaii_str._7	Q976I3.1	NP_376224	Q975U9.1	NP_378073.1	Q971B5.1	Q970Y4.1		Q973C8.1
Thermoproteus_neutrophilus_V24Sta	YP_001795348.1	YP_001794804	B1YCS2.1	YP_001794156.1	B1Y963.1	B1Y9Y4.1		B1YD09.1
Thermofilum_pendens_Hrk_5	YP_920035	YP_919701	YP_920064		YP_920221	YP_919820		YP_920159
Pyrobaculum_aerophilum_str._IM2	NP_558783.1	AAL64073.1	AAL63558.1	NP_559586.1	NP_559815.1	AAL64715.1		AAL63698.1
Archaeoglobus_fulgidus_DSM_4304	O29342	NP_069749	NP_070470		O28664	NP_070859		NP_069384
Halobacterium_salinarum_R1	YP_001688611.1	CAP14760.1	B0R6V2.1		YP_001688566.1	B0R6Z8.1		B0R6G6.1
Halorubrum_lacusprofundi_ATCC_49239	ACM57242.1	YP_002564968	YP_002566866.1		YP_002565447.1	YP_002566721.1		YP_002566413.1
Methanocaldococcus_jannaschii_DSM_2661	AAB99575.1	NP_247199	NP_247994.1		NP_248233.1		NP_248070.1	NP_248192.1
Methanococcus_maripaludis_S2	CAF31172.1	NP_987332	NP_988734.1		NP_987816.1		NP_987999.1	NP_987534.1
Methanopyrus_kandleri_AV19	AAM01924.1	NP_613439.1	NP_614821.1		NP_614512.1		NP_614743.1	NP_613829.1
Thermococcus_kodakarensis_KOD1	YP_182905.1	YP_183391	CAT70479.1		CAT72658.1	YP_183553.1		YP_183583.1
Pyrococcus_furiosus_DSM_3638	NP_578598.1	NP_579356	NP_577993.1		NP_579022.1	NP_578933.1		NP_579080.1
Pyrococcus_horikoshii_OT3	NP_142932.1	NP_143467	NP_142278.1		NP_142919.1	NP_142656.1		NP_142646.1
Thermoplasma_acidophilum_DSM_1728	NP_394405.1	NP_613439	NP_393577.1		NP_394395.1	NP_393946.1		NP_393810.1
Picrophilus_torridus_DSM_9790	YP_023992	YP_023892	YP_023290		YP_024142	YP_024010		YP_023372
Candidatus_Korarchaeum_cryptofilum_OPF8	YP_001737629	YP_001737665	YP_001737676		YP_001737624	YP_001737372		YP_001737367
Nanoarchaeum_equitans_Kin4-M	NP_963815.1	AAR39262.1	AAR38959.1		NP_963502.1	AAR39157.1		AAR39031.1
Cenarchaeum_symbiosum_A	YP_875187, YP_875548	YP_876837	YP_876645		YP_875309	YP_875049		YP_875197
Nitrosopumilus_maritimus_SCM1	YP_001582205.1, YP_001582400.1	ABX11963.1	ABX12132.1		YP_001581992.1	A9A4V3.1		A9A2B1.1
Candidatus_Nitrosoarchaeum_koreensis	ZP_08668019, ZP_08668263	EGP92792	EGP92965		ZP_08667689	EGP94373		EGP93821
Mycobacterium_tuberculosis_H37Rv	CAB01271.1	NP_216873	NP_217096.1	NP_218115.1, NP_216156.1	NP_217361.1	NP_218351.1		NP_217130.1
Streptomyces_coelicolor_A3	Q9F323	Q9L2H9	Q9KXP2		NP_629827	Q9ZBX1		NP_625810
Nocardia_farcinica	YP_119861	Q5YZT7	Q5YTH9	YP_116617	YP_120286	Q5Z3K9		YP_119924
Aquifex_aeolicus	O67589		NP_213082	O67258	NP_213250	CAY05312		AAC07549
Hydrogenobacter_thermophilus	BAI68819.1		YP_003432147.1	BAI68641.1	YP_003432908.1	YP_003433243.1		YP_003432950.1
Bacteroides_fragilis_YCH46	YP_099662	YP_097695	YP_100693	YP_101086	YP_099717	YP_098291		YP_098968
Flavobacterium_johnsoniae_UW101	A5FKJ2.1	YP_001193149	YP_001197370.1	ABQ04951.1	A5FA31.1	YP_001193200.1		YP_001192381.1
Chlorobaculum_parvum_NCIB_8327	ACF11743	ACF10451	ACF12190	ACF11192	ACF11976	ACF11043		ACF10546
Chlorobium_phaeobacteroides_DSM_266	ABL65671	ABL64115	ABL64423	ABL65756	ABL65893	ABL65782,		YP_912913
Prosthecochloris_aestuarii_DSM_271	B4S8V2.1	YP_002014737	B4S4H1.1	YP_002016175.1	B4S9D5.1	B4S6M3.1		B4S3D2.1
Chlamydia_trachomatis_D/UW-3/CX	NP_220057		NP_220058	NP_220300	NP_219903	NP_220248		NP_220096
Chlamydophila_pneumoniae_CWL029	NP_224858		NP_224859	NP_225126	NP_224696	NP_225065		NP_225001
Akkermansia_muciniphila_ATCC_BAA-835	ACD05220	ACD05592	ACD05221	ACD04632	ACD03881	ACD05924		ACD05550
Methylacidiphilum_infernorum_V4	YP_001938740	ACD83294.1	YP_001938739	ACD83350.1	ACD83744.1	YP_001940375		YP_001940627
Chloroflexus_aurantiacus_J-10-fl	ABY35529	YP_001636083	ABY36141	ABY34455	ABY34538	ABY33694		ABY34425
Dehalococcoides_ethenogenes_195	YP_181449.1	AAW39908.1	AAW39179.1	YP_181320.1	YP_181114.1	AAW40145.1		AAW39977.1
Thermomicrobium_roseum_DSM_5159	YP_002522081.1	B9L0I4.1	YP_002522253.1	YP_002522097.1	YP_002521851.1	B9L131.1		YP_002522276.1
Synechococcus_elongatus_PCC6301	YP_170950.1		YP_172545.1	YP_172401.1	YP_172846.1	YP_171779.2		YP_172214.1
Thermosynechococcus_elongatus	NP_681934.1		NP_682688.1	NP_681003.1	NP_681566.1	NP_682342.1		NP_682992.1
Anabaena_variabilis	ABA19873		ABA21878, ABA20609	ABA22758	ABA21929	ABA21346		ABA24938
Deinococcus_radiodurans	NP_294779.1, NP_295070.1	AAF11606.1	AAF10919.1	NP_294095.1	NP_294990.1	AAF10848.1		AAF11630.1
Thermus_thermophilus_HB27	YP_005056.1, Q72KH6.1	Q72L85.1	YP_004335	Q72JT9.1	Q72GF9.1	P34945.2		Q72HH1.1
Elusimicrobium_minutum	ACC97941.1		YP_001875279.1	ACC97625.1	YP_001875576.1	YP_001876388.1		YP_001875458.1
Acidobacterium_capsulatum	ACO33733		ACO33958	ACO32608	ACO33322	ACO33447		ACO34358
Candidatus_Solibacter_usitatus	ABJ82529		ABJ82528	ABJ82032	ABJ82833	ABJ83472		YP_828491
Fibrobacter_succinogenes_subsp. succinogenes_S85	ACX76416.1	YP_003251124	YP_003250680.1	ACX76218.1	YP_003250966.1	YP_003250463.1		YP_003250157.1
Bacillus_subtilis	BAI86239		CAB14715	P37477	NP_389539	BAA05249		AAA22863, AAA22864
Clostridium_acetobutylicum	AAK80921.1, AAK80226.1	AAK81132	AAK80686.1	AAK81133.1	AAK81115.1	AAK78008.1, AAK78004.1		AAK80318.1
Streptococcus_pyogenes_M1	NP_270072.1		NP_270073.1	NP_268853.1	NP_269934.1	NP_269765.1		NP_268795.1
Fusobacterium_nucleatum	AAL94505.1		NP_603205.1	AAL94662.1	NP_602474.1	NP_603020.2		NP_603508.1
Streptobacillus_moniliformis	YP_003306406.1		YP_003306407.1	YP_003306376.1	YP_003305686.1	YP_003305397.1		YP_003305752.1
Gemmatimonas_aurantiaca_T-27	BAH38757.1	YP_002760421	YP_002761266.1	BAH38621.1	BAH38795.1	YP_002761055.1		YP_002760797.1
Thermodesulfovibrio_yellowstonii	B5YJP5.1		B5YHK1.1	YP_002249051.1	B5YKW0.1	B5YGU2.1		YP_002249047.1
Candidatus_Nitrospira_defluvii	WP_013247998		WP_013250876	WP_013248595	WP_041186451	WP_013248730		WP_041186861
Pirellula_staleyi_DSM_6068	ADB19348	ADB19011	ADB16850	ADB15333	ADB19307	ADB17076		ADB16173
Rhodopirellula_baltica_SH_1	Q7UFY6	NP_869478	Q7UZ20	NP_869666	NP_866551	CAD71875		NP_870331
Agrobacterium_tumefaciens_C58	Q8UG87		NP_353700	Q8U7H6	Q8UFV9	NP_354691		NP_354730
Brucella_melitensis_16M	ZP_05834278		AAL54298		NP_540057, ZP_05834339	EEW89019		AAL52096
Rhizobium_etli_CFN_42	YP_469027		YP_468359	YP_471139	YP_469149	YP_469346		YP_469704
Rickettsia_prowazekii	NP 220536.1		NP_220692.1		NP_220765.1	NP_221133.1		NP_220607.1
Magnetococcus_sp._MC-1	A0L667.1		AAW39896.1	A0L6F7.1	YP_865681.1	YP_864997.1		YP_864295.1
Burkholderia_mallei_ATCC_23344	AAU49011		AAU47557	AAU47811	AAU50103	AAU49997		AAU49312
Nitrosomonas_europaea	NP_842254.1		NP_840246.2	NP_842352.1	NP_841366.1	CAD84091.1		CAD84869.1
Escherichia_coli_K12_MG1655	AAC74936.1		NP_417009.1	AAA97029.1, AAC75928.1	NP_414736.1	NP_415413.1		NP_416234.1
Pseudomonas_putida_GB-1	ABZ00094		ABY96807	ABY97009	ABZ00102	ABY99496		ABY99372
Azotobacter_vinelandii	ACO79820		ACO80164	CBV26804	ACO79822	ACO78990		ACO78245
Geobacter_sulfurreducens	AAR34837.1		NP_952710.1	AAR35647.1	NP_952511.1	NP_951099.1		NP_952566.1
Myxococcus_xanthus	YP 632850		YP_631910	YP_632893	YP_634772	YP_630179		YP_631780
Campylobacter_jejuni_NCTC11168	YP_002344069		YP_002344172	YP_002343838	YP_002343974	YP_002343826		YP_002343664
Helicobacter_pylori_J99	AAD06143.1		Q9ZK27.1	AAD05751.1	NP_222944.1	Q9ZJD9.1		Q9ZMV3.1
Leptospira_interrogans_serovar_Lai_str._56601	AAN48879.1	NP_711569	NP_710235.1, NP 711292.1	AAN49194.1	NP_713470.1	NP_714519.1		NP_711421.1
Borrelia_burgdorferi_B31	NP_212580	NP_212505	NP_212269		NP_212536	NP_212360		AAC67076
Thermanaerovibrio_acidaminovorans	YP_003317100.1		YP_003317099.1	YP_003317632.1	YP_003316832.1	YP_003317520.1		YP_003317238.1
Mycoplasma_genitalium_G37	ZP 05405573.1	NP 072916.1	ZP_05405572.1	AAC71353.1	NP_072950	NP_072665.1		NP_073047.1
Ureaplasma_parvum_serovar_3_str._ATCC_700970	NP_078120	NP_078330	NP_078119	NP_077892	NP_078289	NP_077937		NP_078372
Thermotoga_maritima	NP 229240.1		NP_228896.1	NP_229505.1	NP_228324.1	NP_229180.1		NP_228549.1
Kosmotoga_olearia	ACR78936.1		YP_002940105.1	ACR80711.1	YP_002941401.1	YP_002940568.1		YP_002941770.1

Supplemental Table S2. Collected sequence list of seven aminoacyl tRNA synthetases used in alignment B. Numbers colored in red were excluded from final alignment.

Supplemental Table S3. Amino acid sequence alignment of seven ARSs (LysRS, AspRS, HisRS, GlyRS-1, ThrRS, ProRS, and SerRS). (3a) Original alignment A of seven ARSs. (3b) The N-terminal region of SerRS was transferred to the C-terminus and aligned with the anticodon binding region of other ARSs in alignment A. (3c) Alignment B of seven ARSs after the N-terminal region of SerRS was transferred to the C-terminus.
The alignments were used for phylogenetic analyses and inference of ancestral sequences. The region shaded in blue is the anticodon binding domain. The region shaded in yellow is the editing domain. The region shaded in pink is the tRNA binding domain, transferred from the N-terminal region of SerRSs to the C-terminus. The region shaded in orange is the catalytic domain. The three strongly conserved motifs proposed by Eriani et al. (1990) are shown above.

Supplemental Table S4. Substrate amino acid interaction sites of extant ARS, interacting portion of the substrate amino acid, and indication of presence of ion in respective contemporary ARSs.

Archaea and Bacteria are shown in red and pale blue, respectively. The amino acid residues interacting with substrate amino acid side chain and a zinc ion in crystal structures of contemporary ARSs are shown in orange and blue, respectively. The corresponding residues in other ARSs are shown in black. The amino acid residues colored pink are predicted to interact with a substrate amino acid side chain and a zinc ion from the structure of homologous ARS. The number at the top of column indicates the column number in alignment, using phylogenetic analyses (Supplemental Table S3c).

organism and ARS	408	541	543	546	597	599	601	935	978	979	1176	1305	1307	1308	1309
T. kodakaraensis Asprs	E170	S190	Q192	K195	S229	D231	E233	Y339	E348	I349	R368	–	G405	F406	G407
E. coli Asprs	E171	S193	Q195	K198	Q231	D233	E235	G440	A469	V470	R489	–	G530	L531	A532
E. coli Lysrs	G217	A239	E241	L244	M277	E279	Y281	A401	E409	I410	E429	–	G474	L475	G476
T. acidophilum Hisrs	Y53	E81	T83	T86	Q124	N126	D128	–	Y270	T271	G290	–	G309	F310	G311
T. thermophilus Hisrs	F50	E81	T83	M86	Q126	N128	E130	Y263	Y264	V265	G285	–	G304	F305	A306
T. thermophilus Glyrs	L75	E189	A191	I194	Q238	E240	E242	–	Y288	A289	R312	–	E360	P361	S362
M. jannaschii Thrrs	P259	A287	C289	Q292	M337	D339	H341	–	Y416	W417	V438	H465	S467	P468	T469
E. coli Thrrs	W304	M332	C334	H337	Q381	D383	H385	–	F461	Y462	V483	H511	A513	I514	L515
M. maripaludis Prors	L57	T101	E103	I110	F150	E152	H154	–	D200	Y201	V220	–	C249	Y250	G251
E.coli Prors	W80	T109	E111	I114	M157	D159	Y161	D219	D228	Y229	I414	–	C443	Y444	G445
M. kandleri serrs	M256	A317	C319	F322	R366	E368	V370	E417	V426	P427	F456	–	C478	A479	G480
T. thermophilus serrs	F199	T225	E227	L230	K277	E279	Y281	–	W328	R329	C349	–	N379	T380	A381

interaction site of amino acid	408	541	543	546	597	599	601	935	978	979	1176	1305	1362	1363	1364
AspRS		H_2O(NH)	NH	side		H_2O(NH)					side				
LysRS	NH		NH/side			NH	side				side				
HisRS		NH	NH		NH		side	NH	side						
GlyRS		NH				NH							side		NH
ThrRS			Zn			side	Zn			NH		Zn			
ProRS		NH/side	NH/side												
SerRS Rare		NH	Zn		side	Zn							Zn		
SerRS basic		NH	NH			NH/side								side	

Supplemental Table S5.

Posterior probability of residues at the substrate and ion interaction sites in ancestral ARSs, based on the ML tree (a) and the Bayesian tree (b) from alignment A, and the ML tree (c) from alignment B. Predicted amino acid residues of the ancestral ARSs generated in two different trees by five methods, at the substrate and ion interaction sites shown in Table 1 or Supplemental Table S4. The predicted posterior probability of the residue appearing at the site is shown in the pair of rows from the best to the fourth highest probability.

(a)Based on the ML tree from alignment A

		398	544	546	549	603	605	607	967	968	1027	1253	1361	1362	1363	1364
AncDK_nhphylobayes	Best	G	S	Q	K	Q	D	E	Y	P	L	R	-	G	W	G
		0.987	0.990	0.668	0.704	0.618	0.698	0.888	0.439	0.999	0.343	0.653		0.992	0.330	0.982
	second	E	A	E	L	S	E	Y	W	Y	I	E	-	C	I	A
		0.004	0.003	0.242	0.209	0.289	0.268	0.088	0.244	0.001	0.213	0.243		0.004	0.254	0.013
	Third	K	E	C	I	M	S	D	F	L	R	D	-	N	L	S
		0.001	0.003	0.028	0.033	0.063	0.007	0.003	0.202	0.000	0.209	0.031		0.001	0.240	0.002
	Fourth	A	D	A	M	L	A	A	H	-	V	N	-	A	V	C
		0.001	0.001	0.013	0.015	0.010	0.005	0.002	0.015		0.046	0.012		0.000	0.050	0.000
AncDK_raxml	Best	G	S	Q	K	M	D	Y	Y	P	L	R	-	G	L	G
		0.998	0.969	0.559	0.353	0.640	0.520	0.572	0.520	1.000	0.346	0.366		0.998	0.280	0.986
	second	A	A	E	L	Q	E	E	W	S	M	E	-	A	M	A
		0.001	0.015	0.413	0.290	0.194	0.467	0.222	0.335	0.000	0.165	0.356		0.001	0.259	0.011
	Third	S	T	K	M	S	N	H	F	A	I	K	-	S	W	S
		0.000	0.008	0.009	0.125	0.062	0.005	0.076	0.133	0.000	0.151	0.115		0.000	0.207	0.002
	Fourth	N	N	D	R	T	Q	Q	H	Q	V	Q	-	N	I	N
		0.000	0.003	0.006	0.066	0.035	0.004	0.034	0.007	0.000	0.088	0.111		0.000	0.101	0.000
AncDK_codeml	Best	G	S	E	L	M	E	Y	Y	P	L	E	-	G	L	G
		0.986	0.722	0.693	0.664	0.368	0.664	0.353	0.819	0.994	0.74	0.645		0.993	0.524	0.978
	second	A	A	Q	M	Q	D	E	F	A	I	K	-	A	M	A
		0.005	0.169	0.216	0.071	0.163	0.314	0.251	0.101	0.002	0.105	0.109		0.003	0.137	0.013
	Third	S	T	D	I	T	Q	H	W	S	V	R	-	S	F	S
		0.003	0.051	0.026	0.064	0.092	0.007	0.101	0.072	0.002	0.048	0.083		0.002	0.123	0.005
	Fourth	N	N	K	K	L	N	D	H	-	M	Q	-	N	I	N
		0.002	0.018	0.026	0.051	0.077	0.006	0.073	0.005		0.040	0.078		0.001	0.091	0.001
AncDK_phyml	Best	G	S	E	L	Q	E	E	Y	P	L	E	-	G	L	G
		0.970	0.981	0.530	0.725	0.675	0.523	0.770	0.720	0.996	0.698	0.707		1.000	0.570	0.982
	second	A	A	Q	K	M	D	Y	F	A	S	R	-	A	I	A
		0.012	0.011	0.444	0.084	0.208	0.476	0.125	0.164	0.001	0.134	0.137		0.000	0.111	0.017
	Third	S	T	A	M	S	N	H	W	S	A	K	-	S	M	S
		0.007	0.005	0.007	0.068	0.055	0.001	0.034	0.109	0.001	0.052	0.060		0.000	0.110	0.001
	Fourth	N	N	K	I	T	Q	D	H	T	H	Q	-	-	F	N
		0.004	0.001	0.007	0.056	0.019	0.001	0.025	0.004	0.000	0.037	0.046			0.088	0.000
AncDK_iqtree	Best	G	S	E	L	Q	E	E	Y	P	L	E	-	G	L	G
		0.975	0.999	0.582	0.676	0.758	0.799	0.913	0.812	0.998	0.710	0.806		1.000	0.637	0.983
	second	A	T	Q	K	M	D	Y	F	S	I	R	-	A	M	A
		0.010	0.001	0.406	0.103	0.183	0.201	0.059	0.122	0.001	0.137	0.097		0.000	0.107	0.014
	Third	S	A	A	M	S	Q	H	W	A	V	K	-	S	I	S
		0.006	0.000	0.009	0.070	0.039	0.000	0.015	0.062	0.000	0.053	0.032		0.000	0.105	0.002
	Fourth	N	E	T	I	T	N	Q	H	H	M	D	-	N	F	N
		0.003	0.000	0.001	0.050	0.006	0.000	0.005	0.003	0.000	0.043	0.027		0.000	0.065	0.000
AncHG_nhphylobayes	Best	E	E	C	I	Q	E	E	H	Y	Y	D	-	C	P	G
		0.450	0.920	0.382	0.682	0.998	0.587	0.973	0.374	0.999	0.366	0.925		0.398	0.284	0.552
	second	I	T	A	V	M	D	H	Y	F	S	N	-	G	S	S
		0.104	0.044	0.292	0.163	0.001	0.296	0.021	0.278	0.001	0.255	0.060		0.335	0.220	0.248
	Third	G	A	T	M	F	G	A	A	H	T	E	-	E	F	A
		0.089	0.010	0.151	0.040	0.000	0.088	0.001	0.269	0.000	0.113	0.004		0.210	0.190	0.123
	Fourth	K	M	E	H	I	A	R	F	-	A	R	-	S	Y	I
		0.087	0.008	0.089	0.035	0.000	0.004	0.001	0.013		0.073	0.003		0.017	0.105	0.028
AncHG_raxml	Best	E	E	T	I	Q	G	E	H	Y	T	D	-	G	P	S
		0.229	0.962	0.473	0.487	0.978	0.445	0.963	0.550	0.983	0.500	0.977		0.445	0.354	0.467
	second	Q	D	A	V	H	E	D	Y	F	S	E	-	E	F	A
		0.159	0.020	0.325	0.448	0.006	0.305	0.020	0.430	0.010	0.337	0.011		0.305	0.311	0.419
	Third	D	Q	S	M	E	D	Q	F	H	A	N	-	D	Y	T
		0.109	0.011	0.112	0.022	0.005	0.092	0.011	0.008	0.004	0.104	0.010		0.092	0.064	0.042
	Fourth	K	K	V	L	K	A	K	N	W	N	S	-	A	L	C
		0.105	0.003	0.023	0.022	0.003	0.043	0.003	0.003	0.002	0.013	0.001		0.043	0.043	0.023
AncHG_codeml	Best	E	E	A	I	Q	D	E	Y	Y	T	D	-	G	F	G
		0.685	0.674	0.409	0.454	0.906	0.545	0.537	0.576	0.851	0.238	0.821		0.579	0.369	0.455
	second	Q	T	T	V	M	E	H	H	F	Y	N	-	A	Y	A
		0.239	0.091	0.208	0.304	0.035	0.405	0.192	0.344	0.141	0.217	0.13		0.143	0.307	0.359
	Third	K	D	S	L	K	G	Q	F	H	S	E	-	C	A	S
		0.033	0.064	0.199	0.136	0.015	0.02	0.127	0.032	0.003	0.191	0.041		0.125	0.079	0.156
	Fourth	D	A	C	M	R	N	D	A	L	A	Q	-	S	S	T
		0.022	0.05	0.072	0.092	0.014	0.014	0.086	0.01	0.002	0.109	0.002		0.088	0.068	0.012
AncHG_phyml	Best	E	E	A	I	Q	E	E	Y	Y	T	D	-	G	F	A
		0.623	0.890	0.648	0.654	0.983	0.563	0.934	0.468	0.875	0.253	0.906		0.867	0.291	0.449
	second	Q	T	T	V	M	D	H	H	F	S	N	-	A	Y	G
		0.155	0.060	0.178	0.238	0.016	0.430	0.031	0.452	0.120	0.248	0.089		0.061	0.258	0.361
	Third	K	A	S	M	K	G	Q	F	H	Y	E	-	C	S	S
		0.076	0.022	0.100	0.083	0.000	0.005	0.022	0.025	0.002	0.199	0.005		0.032	0.144	0.186
	Fourth	D	S	E	L	R	N	D	A	L	A	Q	-	S	A	T
		0.073	0.009	0.040	0.024	0.000	0.001	0.012	0.015	0.001	0.112	0.000		0.028	0.125	0.002
AncHG_iqtree	Best	E	E	A	I	Q	E	E	H	Y	S	D	-	G	F	A
		0.665	0.979	0.774	0.591	0.987	0.855	0.990	0.539	0.941	0.243	0.945		0.938	0.253	0.436
	second	Q	T	T	V	M	D	H	Y	F	Y	N	-	A	S	G
		0.155	0.014	0.135	0.260	0.013	0.144	0.006	0.429	0.057	0.220	0.052		0.031	0.248	0.344
	Third	K	A	E	M	K	G	Q	F	H	T	E	-	C	Y	S
		0.062	0.004	0.070	0.100	0.000	0.001	0.003	0.012	0.001	0.217	0.003		0.014	0.184	0.209
	Fourth	D	S	S	L	R	N	D	A	L	A	Q	-	S	A	T
		0.062	0.001	0.016	0.046	0.000	0.000	0.001	0.007	0.000	0.116	0.000		0.013	0.163	0.004

AncHGSPT_nhphylobayes	Best	E	E	C	I	Q	E	E	A	Y	Y	D	-	C	S	G
		0.441	0.742	0.504	0.648	0.989	0.546	0.901	0.342	0.991	0.476	0.701		0.503	0.276	0.684
	second	G	T	A	V	M	D	H	H	F	S	N	-	G	P	S
		0.119	0.140	0.200	0.157	0.008	0.372	0.083	0.321	0.003	0.191	0.243		0.310	0.204	0.162
	Third	K	A	T	M	F	G	V	Y	E	T	R	-	E	F	A
		0.093	0.033	0.109	0.052	0.001	0.060	0.004	0.256	0.001	0.089	0.014		0.140	0.150	0.074
	Fourth	I	M	E	H	R	A	D	F	R	A	E	-	S	Y	I
		0.079	0.031	0.106	0.047	0.001	0.004	0.002	0.015	0.001	0.060	0.012		0.012	0.138	0.034
AncHGSPT_raxml	Best	E	E	A	I	Q	D	E	H	Y	S	D	-	C	S	A
		0.418	0.897	0.411	0.477	0.978	0.512	0.752	0.830	0.874	0.342	0.826		0.556	0.394	0.559
	second	Q	T	C	V	M	E	H	Y	F	T	N	-	G	Y	S
		0.203	0.044	0.277	0.256	0.020	0.452	0.130	0.139	0.119	0.326	0.170		0.241	0.148	0.302
	Third	D	Q	T	M	H	G	Q	A	W	Y	E	-	A	P	G
		0.102	0.020	0.193	0.214	0.001	0.022	0.078	0.009	0.003	0.165	0.003		0.091	0.140	0.108
	Fourth	K	A	S	L	K	N	D	S	H	A	H	-	S	F	P
		0.092	0.014	0.099	0.039	0.001	0.006	0.030	0.007	0.003	0.061	0.001		0.062	0.132	0.012
AncHGSPT_codeml	Best	E	E	A	I	Q	D	E	Y	Y	T	D	-	G	F	G
		0.685	0.674	0.409	0.454	0.906	0.545	0.537	0.576	0.851	0.238	0.821		0.579	0.369	0.455
	second	Q	T	T	V	M	E	H	H	F	Y	N	-	A	Y	A
		0.239	0.091	0.208	0.304	0.035	0.405	0.192	0.344	0.141	0.217	0.13		0.143	0.307	0.359
	Third	K	D	S	L	K	G	Q	F	H	S	E	-	C	A	S
		0.033	0.064	0.199	0.136	0.015	0.020	0.127	0.032	0.003	0.191	0.041		0.125	0.079	0.156
	Fourth	D	A	C	M	R	N	D	A	L	A	Q	-	S	S	T
		0.022	0.050	0.072	0.092	0.014	0.014	0.086	0.010	0.002	0.109	0.002		0.088	0.068	0.012
AncHGSPT_phyml	Best	E	E	A	I	Q	E	E	Y	Y	Y	D	-	G	F	G
		0.620	0.598	0.638	0.633	0.937	0.552	0.812	0.452	0.833	0.314	0.830		0.855	0.275	0.442
	second	Q	T	T	V	M	D	H	H	F	T	N	-	A	Y	A
		0.153	0.267	0.168	0.221	0.061	0.444	0.136	0.436	0.155	0.198	0.160		0.058	0.273	0.405
	Third	K	A	S	M	L	G	Q	F	L	S	E	-	C	S	S
		0.076	0.074	0.103	0.113	0.001	0.002	0.029	0.031	0.003	0.190	0.008		0.051	0.150	0.149
	Fourth	D	S	E	L	K	N	D	A	H	A	Q	-	S	A	P
		0.074	0.033	0.052	0.031	0.000	0.001	0.014	0.026	0.003	0.090	0.001		0.027	0.130	0.002
AncHGSPT_iqtree	Best	E	T	A	I	Q	E	E	H	Y	Y	D	-	G	S	G
		0.662	0.492	0.755	0.563	0.915	0.851	0.922	0.528	0.905	0.352	0.861		0.930	0.259	0.417
	second	Q	E	T	V	M	D	H	Y	F	S	N	-	A	F	A
		0.153	0.363	0.124	0.241	0.084	0.149	0.069	0.419	0.088	0.177	0.131		0.029	0.238	0.399
	Third	D	A	E	M	L	G	Q	A	V	T	E	-	C	Y	S
		0.063	0.093	0.097	0.134	0.000	0.000	0.005	0.016	0.001	0.162	0.006		0.026	0.196	0.173
	Fourth	K	S	S	L	K	N	D	F	L	A	Q	-	S	A	T
		0.063	0.048	0.016	0.056	0.000	0.000	0.002	0.016	0.001	0.088	0.000		0.012	0.170	0.004
AncSTP_nhphylobayes	Best	E	T	C	I	Q	D	H	A	Y	Y	N	-	C	S	G
		0.447	0.451	0.791	0.580	0.929	0.502	0.953	0.751	0.975	0.884	0.871		0.976	0.415	0.788
	second	G	E	E	V	M	E	E	H	F	S	D	-	G	Y	I
		0.170	0.279	0.150	0.127	0.060	0.474	0.028	0.179	0.009	0.025	0.071		0.010	0.228	0.064
	Third	K	M	A	H	F	G	V	Y	V	F	E	-	R	A	S
		0.101	0.120	0.026	0.087	0.005	0.012	0.009	0.024	0.004	0.014	0.015		0.008	0.213	0.062
	Fourth	A	A	T	M	R	A	Y	T	I	T	R	-	E	P	P
		0.086	0.095	0.017	0.087	0.001	0.003	0.004	0.018	0.003	0.011	0.010		0.004	0.051	0.036
AncSTP_raxml	Best	E	T	C	M	M	D	H	A	F	Y	N	-	C	S	G
		0.239	0.701	0.763	0.463	0.957	0.815	0.984	0.613	0.384	0.971	0.908		1.000	0.598	0.616
	second	Q	M	E	H	Q	E	Y	S	M	F	H	-	R	A	P
		0.163	0.177	0.078	0.183	0.029	0.182	0.015	0.153	0.164	0.015	0.027		0.000	0.194	0.129
	Third	K	A	A	Q	W	N	Q	T	I	W	Q	-	S	Y	A
		0.106	0.080	0.055	0.144	0.004	0.001	0.000	0.103	0.121	0.011	0.027		0.000	0.103	0.105
	Fourth	D	S	S	L	R	Q	N	C	Y	H	K	-	A	T	S
		0.094	0.021	0.035	0.047	0.003	0.001	0.000	0.028	0.111	0.003	0.016		0.000	0.037	0.044
AncSTP_codeml	Best	E	T	A	I	Q	D	H	A	Y	Y	N	-	C	Y	G
		0.692	0.573	0.363	0.308	0.751	0.68	0.933	0.253	0.577	0.896	0.491		0.962	0.471	0.57
	second	Q	A	S	M	M	E	Q	H	F	F	D	-	G	F	A
		0.234	0.171	0.189	0.258	0.162	0.315	0.023	0.239	0.379	0.063	0.421		0.012	0.189	0.295
	Third	K	S	C	V	R	N	Y	Y	L	H	E	-	A	S	S
		0.037	0.096	0.176	0.195	0.027	0.003	0.022	0.201	0.016	0.017	0.054		0.011	0.111	0.096
	Fourth	D	E	T	L	K	Q	N	S	I	W	Q	-	S	A	P
		0.015	0.069	0.114	0.187	0.02	0.001	0.007	0.125	0.009	0.008	0.01		0.01	0.11	0.013
AncSTP_phyml	Best	E	T	A	I	Q	D	H	H	Y	Y	N	-	C	Y	G
		0.635	0.732	0.602	0.543	0.789	0.505	0.988	0.322	0.614	0.920	0.788		0.995	0.380	0.502
	second	Q	A	T	M	M	E	Y	A	F	F	D	-	G	S	A
		0.155	0.177	0.127	0.244	0.208	0.495	0.006	0.294	0.351	0.048	0.195		0.003	0.215	0.370
	Third	K	E	S	V	R	N	Q	Y	L	H	E	-	A	A	S
		0.077	0.039	0.112	0.168	0.001	0.000	0.004	0.142	0.012	0.013	0.011		0.001	0.164	0.112
	Fourth	D	S	C	L	K	-	E	S	I	W	Q	-	S	F	P
		0.059	0.023	0.076	0.035	0.001		0.002	0.111	0.007	0.006	0.003		0.001	0.143	0.010
AncSTP_iqtree	Best	E	T	A	I	Q	E	H	H	Y	Y	N	-	C	S	G
		0.674	0.791	0.742	0.450	0.775	0.832	0.996	0.413	0.749	0.910	0.845		0.998	0.346	0.490
	second	Q	A	T	M	M	D	Y	A	F	F	D	-	G	Y	A
		0.156	0.159	0.117	0.290	0.223	0.168	0.002	0.317	0.230	0.052	0.144		0.002	0.280	0.363
	Third	K	E	E	V	K	-	E	Y	M	H	E	-	A	A	S
		0.063	0.023	0.101	0.173	0.001		0.001	0.108	0.005	0.015	0.007		0.000	0.201	0.125
	Fourth	D	S	S	L	R	-	Q	S	I	W	Q	-	S	F	P
		0.050	0.015	0.019	0.069	0.001		0.001	0.094	0.005	0.007	0.002		0.000	0.118	0.010

AncSP_nhphylobayes	Best	E	T	C	I	Q	E	H	A	Y	Y	N	-	C	Y	G
		0.394	0.938	0.677	0.588	0.497	0.600	0.948	0.806	0.824	0.945	0.885		0.997	0.417	0.997
	second	G	A	E	M	M	D	V	H	V	I	D	-	G	S	I
		0.282	0.033	0.314	0.163	0.205	0.392	0.018	0.085	0.053	0.008	0.037		0.002	0.378	0.002
	Third	K	E	T	V	F	G	Y	T	I	S	K	-	E	A	S
		0.059	0.012	0.004	0.117	0.128	0.004	0.014	0.049	0.049	0.007	0.017		0.000	0.159	0.000
	Fourth	A	M	A	H	W	A	E	S	A	F	H	-	-	F	-
		0.045	0.007	0.002	0.041	0.048	0.001	0.013	0.014	0.028	0.006	0.013			0.015	
AncSP_raxml	Best	E	T	E	M	M	E	H	A	I	Y	N	-	C	Y	G
		0.131	0.974	0.964	0.552	0.474	0.853	0.472	0.331	0.266	0.780	0.507		0.999	0.842	0.999
	second	D	S	D	I	W	D	Y	T	M	H	H	-	G	S	A
		0.104	0.014	0.014	0.216	0.320	0.141	0.379	0.268	0.246	0.089	0.211		0.000	0.038	0.001
	Third	Q	A	Q	L	F	Q	V	S	V	F	K	-	A	H	S
		0.101	0.006	0.009	0.095	0.057	0.004	0.034	0.204	0.230	0.025	0.113		0.000	0.030	0.000
	Fourth	K	V	A	V	R	N	F	N	L	P	Q	-	S	F	N
		0.082	0.001	0.004	0.081	0.048	0.001	0.033	0.035	0.103	0.014	0.112		0.000	0.020	0.000
AncSP_codeml	Best	E	T	E	I	Q	D	H	A	I	Y	N	-	C	Y	G
		0.666	0.807	0.543	0.347	0.547	0.593	0.906	0.372	0.219	0.939	0.662		0.993	0.626	0.966
	second	Q	A	A	M	M	E	Y	S	V	F	D	-	G	F	A
		0.265	0.081	0.163	0.306	0.227	0.404	0.064	0.227	0.175	0.03	0.202		0.003	0.106	0.025
	Third	K	S	C	L	R	N	Q	T	F	H	E	-	A	S	S
		0.028	0.075	0.081	0.173	0.076	0.001	0.013	0.159	0.173	0.016	0.038		0.002	0.094	0.008
	Fourth	D	E	S	V	K	N	N	H	L	S	K	-	S	A	N
		0.016	0.011	0.079	0.152	0.044	0.001	0.005	0.087	0.159	0.003	0.033		0.001	0.075	0.001
AncSP_phyml	Best	E	T	E	I	Q	E	H	A	I	Y	N	-	C	Y	G
		0.611	0.918	0.625	0.565	0.661	0.573	0.981	0.404	0.198	0.948	0.907		0.999	0.491	0.980
	second	Q	A	A	M	M	D	Y	S	V	F	D	-	G	S	A
		0.183	0.068	0.242	0.262	0.276	0.427	0.015	0.207	0.176	0.025	0.061		0.001	0.200	0.016
	Third	K	S	C	V	R	-	Q	T	F	H	H	-	A	A	S
		0.067	0.010	0.049	0.143	0.027		0.002	0.131	0.174	0.014	0.009		0.000	0.128	0.004
	Fourth	D	E	S	L	K	-	E	H	Y	S	Q	-	A	F	N
		0.060	0.002	0.042	0.028	0.021		0.001	0.130	0.149	0.004	0.008		0.000	0.086	0.000
AncSP_iqtree	Best	E	T	E	I	Q	E	H	A	V	Y	N	-	C	Y	G
		0.645	0.945	0.722	0.476	0.652	0.863	0.993	0.431	0.300	0.941	0.942		1.000	0.369	0.969
	second	Q	A	A	M	M	D	Y	S	I	F	D	-	G	S	A
		0.187	0.054	0.245	0.319	0.295	0.137	0.005	0.235	0.241	0.027	0.036		0.000	0.339	0.024
	Third	K	S	T	V	R	-	E	H	M	H	Q	-	-	A	S
		0.055	0.001	0.015	0.140	0.024		0.000	0.132	0.156	0.016	0.006			0.169	0.007
	Fourth	D	E	C	L	K	-	Q	T	Y	S	H	-	-	F	N
		0.050	0.000	0.012	0.058	0.020		0.000	0.130	0.106	0.004	0.006			0.064	0.000

		398	544	546	549	603	605	607	967	968	1027	1253	1361	1362	1363	1364
ComS_nhphylobayes		E	T	C	V	R	E	V	A	V	Y	N	-	C	S	G
		0.193	0.888	0.695	0.325	0.300	0.917	0.940	0.458	0.315	0.283	0.320		0.975	0.492	1.000
ComS_raxml		Y	T	C	F	K	E	V	D	A	P	H	-	C	S	G
		0.092	0.487	0.477	0.281	0.485	0.938	0.853	0.329	0.267	0.456	0.571		0.662	0.517	0.926
ComS_codeml		E	T	E	I	R	E	V	S	V	P	H	-	C	S	G
		0.173	0.58	0.422	0.272	0.394	0.747	0.411	0.289	0.378	0.168	0.333		0.922	0.333	0.989
ComS_phyml		E	T	E	V	K	E	V	S	V	K	H	-	C	S	G
		0.126	0.867	0.602	0.382	0.482	0.933	0.874	0.332	0.391	0.198	0.466		0.988	0.432	0.997
ComS_iqtree		E	T	E	V	K	E	V	S	V	K	H	-	C	S	G
		0.133	0.940	0.720	0.335	0.488	0.992	0.979	0.465	0.554	0.183	0.489		0.995	0.575	0.994
ComP_nhphylobayes		E	T	E	I	Q	E	H	A	Y	Y	N	-	C	Y	G
		0.396	0.993	0.803	0.611	0.363	0.598	0.955	0.830	0.784	0.987	0.895		1.000	0.909	1.000
ComP_raxml		E	T	E	M	W	E	H	A	M	Y	N	-	C	Y	G
		0.270	0.989	0.986	0.713	0.377	0.530	0.681	0.394	0.238	0.959	0.661		1.000	0.989	0.998
ComP_codeml		E	T	E	I	M	D	H	A	I	Y	N	-	C	Y	G
		0.675	0.913	0.852	0.365	0.435	0.577	0.909	0.433	0.274	0.979	0.742		0.999	0.911	0.993
ComP_phyml		E	T	E	I	M	E	H	A	I	Y	N	-	C	Y	G
		0.625	0.992	0.981	0.594	0.679	0.577	0.980	0.493	0.265	0.988	0.951		1.000	0.940	0.999
ComP_iqtree		E	T	E	I	M	E	H	A	I	Y	N	-	C	Y	G
		0.654	0.999	0.998	0.497	0.724	0.861	0.993	0.517	0.298	0.986	0.970		1.000	0.963	0.997
ComT_nhphylobayes		E	M	C	H	Q	D	H	A	Y	Y	N	H	C	S	I
		0.438	0.394	0.997	0.394	0.928	0.963	1.000	0.770	0.959	0.886	0.908	0.994	0.962	0.484	0.409
ComT_raxml		E	M	C	H	M	D	H	H	F	Y	N	H	C	A	P
		0.213	0.727	1.000	0.693	0.530	0.989	0.997	0.345	0.496	0.495	0.345	0.997	0.725	0.481	0.347
ComT_codeml		E	T	C	Q	Q	D	H	A	Y	Y	N	H	C	A	A
		0.645	0.347	0.947	0.352	0.75	0.897	0.986	0.292	0.562	0.875	0.486	0.947	0.955	0.389	0.385
ComT_phyml		E	T	C	Q	Q	D	H	A	Y	Y	N	H	C	S	A
		0.572	0.458	0.988	0.442	0.786	0.952	1.000	0.330	0.595	0.903	0.793	0.995	0.995	0.429	0.387
ComT_iqtree		E	T	C	Q	Q	D	H	H	Y	Y	N	H	C	S	A
		0.612	0.488	0.998	0.438	0.774	0.982	1.000	0.421	0.720	0.892	0.851	0.999	0.997	0.521	0.397
ComG_nhphylobayes		E	E	A	I	Q	E	E	H	Y	S	D	-	E	P	S
		0.652	1.000	1.000	1.000	1.000	1.000	1.000	1.000	1.000	0.783	1.000		1.000	1.000	1.000
ComG_raxml		E	E	A	I	Q	E	E	H	Y	S	D	-	E	P	S
		0.854	1.000	1.000	0.996	1.000	1.000	1.000	1.000	1.000	0.584	1.000		1.000	1.000	1.000
ComG_codeml		E	E	A	I	Q	E	E	H	Y	S	D	-	E	P	S
		0.986	1	0.999	0.982	1	0.999	0.999	1	1	0.527	1		0.999	1	0.999
ComG_phyml		E	E	A	I	Q	E	E	H	Y	S	D	-	E	P	S
		0.975	1.000	1.000	0.999	1.000	1.000	1.000	1.000	1.000	0.601	1.000		1.000	1.000	1.000
ComG_iqtree		E	E	A	I	Q	E	E	H	Y	S	D	-	E	P	S
		0.981	1.000	1.000	0.998	1.000	1.000	1.000	1.000	1.000	0.585	1.000		1.000	1.000	1.000
ComH_nhphylobayes		Q	E	T	V	Q	G	E	Y	Y	T	D	-	G	F	A
		0.242	1.000	1.000	0.984	1.000	0.998	1.000	0.999	1.000	0.940	1.000		0.998	0.998	0.996
ComH_raxml		Q	E	T	V	Q	G	E	Y	Y	T	D	-	G	F	A
		0.490	0.995	0.999	0.962	0.999	0.999	0.998	0.999	0.999	0.993	0.999		1.000	0.999	0.998
ComH_codeml		Q	E	T	V	Q	G	E	Y	Y	T	D	-	G	F	A
		0.693	0.969	0.985	0.873	0.998	0.992	0.982	0.996	0.997	0.965	0.996		0.998	0.99	0.975
ComH_phyml		Q	E	T	V	Q	G	E	Y	Y	T	D	-	G	F	A
		0.575	0.999	0.998	0.978	1.000	0.998	1.000	0.998	0.999	0.977	1.000		1.000	0.996	0.998
ComH_iqtree		Q	E	T	V	Q	G	E	Y	Y	T	D	-	G	F	A
		0.609	1.000	1.000	0.972	1.000	1.000	1.000	0.999	1.000	0.973	1.000		1.000	0.998	0.996
ComK_nhphylobayes		G	S	E	L	M	E	Y	Y	P	L	E	-	G	L	G
		1.000	0.991	0.999	1.000	0.645	0.999	1.000	1.000	1.000	0.998	1.000		1.000	0.785	1.000
ComK_raxml		P	S	E	L	M	E	Y	Y	P	L	E	-	G	M	G
		1.000	0.871	0.999	0.998	0.970	0.999	1.000	0.986	1.000	0.669	0.999		1.000	0.498	1.000
ComK_codeml		G	S	E	L	M	E	Y	Y	P	L	E	-	G	L	G
		1	0.774	0.991	0.986	0.854	0.985	0.995	0.994	1	0.961	0.991		1	0.645	1
ComK_phyml		G	S	E	L	M	E	Y	Y	P	L	E	-	G	L	G
		0.9997	0.9852	0.9985	0.999	0.9142	0.9978	0.9997	0.9967	1	0.9837	0.9994		1	0.7596	1
ComK_iqtree		G	S	E	L	M	E	Y	Y	P	L	E	-	G	L	G
		1.000	1.000	1.000	0.998	0.912	1.000	1.000	0.999	1.000	0.981	1.000		1.000	0.809	1.000
ComD_nhphylobayes		G	S	Q	K	Q	D	E	W	P	R	R	-	G	W	G
		0.998	1.000	0.986	1.000	0.643	0.981	0.991	0.389	1.000	0.324	0.990		1.000	0.454	0.972
ComD_raxml		G	S	Q	K	Q	D	E	W	P	R	R	-	G	W	G
		0.998	0.982	0.994	0.989	0.480	0.993	0.989	0.800	1.000	0.173	0.992		0.998	0.732	0.627
ComD_codeml		G	S	Q	K	Q	D	E	F	P	L	R	-	G	L	G
		0.999	0.924	0.914	0.896	0.349	0.923	0.918	0.358	1	0.435	0.885		0.999	0.365	0.936
ComD_phyml		G	S	Q	K	Q	D	E	F	P	L	R	-	G	L	G
		0.996	0.999	0.985	0.988	0.796	0.979	0.995	0.387	1.000	0.458	0.979		1.000	0.425	0.968
ComD_iqtree		G	S	Q	K	Q	D	E	F	P	L	R	-	G	L	G
		0.997	1.000	0.998	0.971	0.865	0.993	0.999	0.396	1.000	0.453	0.991		1.000	0.478	0.962

(b)Based on the Bayesian tree from alignment A

		398	544	546	549	603	605	607	967	968	1027	1253	1361	1362	1363	1364
AncDK_nhphylobayes	Best	G	S	Q	K	Q	D	E	F	P	R	R	-	K	V	F
		0.996	0.997	0.995	0.999	0.978	0.982	0.998	0.988	1.000	0.479	0.995		0.677	0.756	0.966
	second	K	A	E	I	S	E	Y	W	-	I	E	-	E	N	I
		0.001	0.001	0.002	5E-04	0.011	0.015	0.001	0.007		0.273	0.002		0.177	0.085	0.018
	Third	E	E	C	L	M	G	T	H	-	S	D	-	A	R	L
		0.001	0.000	0.001	0.000	0.006	0.001	0.000	0.001		0.052	0.000		0.024	0.050	0.004
	Fourth	D	T	K	M	V	A	V	A	-	V	L	-	R	L	V
		0.000	0.000	0.001	0.000	0.001	0.000	0.000	0.001		0.049	0.000		0.021	0.021	0.002
AncDK_raxml	Best	G	S	Q	K	Q	D	E	F	P	V	R	-	G	I	G
		1.000	0.990	0.991	0.983	0.744	0.985	0.983	0.632	1.000	0.155	0.982		1.000	0.456	0.708
	second	A	A	E	R	M	E	Q	W	S	I	K	-	A	W	A
		0.000	0.005	0.007	0.009	0.094	0.013	0.006	0.299	0.000	0.154	0.011		0.000	0.176	0.275
	Third	S	T	H	Q	S	N	D	Y	A	R	Q	-	S	L	S
		0.000	0.003	0.001	0.004	0.090	0.001	0.006	0.064	0.000	0.143	0.004		0.000	0.125	0.011
	Fourth	N	N	K	M	T	S	H	L	Q	M	E	-	N	M	C
		0.000	0.001	0.001	0.001	0.025	0.000	0.001	0.002	0.000	0.128	0.001		0.000	0.089	0.002
AncDK_codeml	Best	G	S	Q	K	Q	D	E	F	P	I	R	-	G	L	G
		0.998	0.897	0.911	0.875	0.475	0.925	0.921	0.673	1.000	0.368	0.868		0.999	0.311	0.93
	second	A	A	E	R	S	E	D	W	-	V	K	-	A	I	A
		0.001	0.055	0.068	0.061	0.200	0.070	0.038	0.173		0.325	0.087		0.001	0.307	0.06
	Third	-	T	K	Q	T	N	Q	Y	-	L	Q	-	-	F	S
			0.030	0.008	0.02	0.078	0.004	0.023	0.150		0.156	0.023			0.113	0.007
	Fourth	-	N	R	L	A	-	K	L	-	M	E	-	-	W	N
			0.008	0.003	0.01	0.058		0.005	0.003		0.043	0.013			0.108	0.001
AncDK_phyml	Best	G	S	Q	K	Q	D	E	F	P	I	R	-	G	I	A
		0.993	0.997	0.996	0.966	0.974	0.992	0.999	0.910	0.999	0.246	0.990		1.000	0.724	0.636
	second	A	A	E	R	S	E	D	W	A	V	K	-	-	L	G
		0.003	0.002	0.004	0.019	0.012	0.008	0.001	0.049	0.000	0.223	0.006			0.14	0.355
	Third	S	T	K	Q	M	N	Q	Y	S	R	E	-	-	V	S
		0.002	0.001	0.000	0.005	0.005	0.000	0.000	0.039	0.000	0.218	0.002			0.057	0.008
	Fourth	N	N	A	M	T	-	-	L	-	L	Q	-	-	F	C
		0.001	0.000	0.000	0.002	0.002			0.002		0.082	0.002			0.03	2E-04
AncDK_iqtree	Best	G	S	Q	K	Q	D	E	F	P	I	R	-	G	I	G
		0.991	0.999	0.997	0.977	0.985	0.991	1.000	0.935	1.000	0.277	0.992		1.000	0.722	0.595
	second	A	A	E	R	S	E	D	W	A	V	K	-	A	L	A
		0.004	0.001	0.003	0.011	0.008	0.009	0.000	0.035	0.000	0.248	0.003		0.000	0.160	0.388
	Third	S	T	A	Q	M	N	Q	Y	S	R	E	-	-	V	S
		0.002	0.000	0.000	0.004	0.005	0.000	0.000	0.029	0.000	0.150	0.003			0.045	0.013
	Fourth	N	N	K	L	T	-	K	L	Q	L	Q	-	-	F	T
		0.001	0.000	0.000	0.002	0.001		0.000	0.001	0.000	0.108	0.001			0.028	0.001
AncHSPT_nhphylobayes	Best	K	E	C	I	Q	E	E	F	Y	Y	D	-	G	S	G
		0.181	0.812	0.994	0.349	0.994	0.563	0.930	0.359	0.994	0.462	0.947		0.459	0.242	0.572
	second	E	T	A	V	M	D	H	A	F	T	N	-	C	F	A
		0.179	0.116	0.002	0.219	0.004	0.339	0.065	0.32	0.001	0.137	0.039		0.394	0.195	0.211
	Third	G	A	T	M	F	G	D	Y	A	A	R	-	E	P	S
		0.146	0.025	0.002	0.194	0.000	0.081	0.001	0.152	0.001	0.122	0.005		0.113	0.154	0.118
	Fourth	Q	M	E	H	T	A	Y	H	I	S	K	-	S	Y	P
		0.141	0.012	0.001	0.070	0.000	0.005	0.001	0.127	0.001	0.081	0.003		0.008	0.146	0.060
AncHSPT_raxml	Best	K	T	C	M	M	D	H	A	Y	Y	N	-	C	Y	G
		0.184	0.517	0.512	0.497	0.566	0.618	0.803	0.358	0.545	0.715	0.684		0.906	0.413	0.411
	second	Q	E	T	I	Q	G	Q	Y	F	T	D	-	G	F	A
		0.164	0.17	0.28	0.128	0.4	0.219	0.069	0.327	0.366	0.106	0.275		0.064	0.322	0.359
	Third	R	A	S	V	L	E	E	H	M	H	E	-	A	S	P
		0.146	0.11	0.089	0.12	0.008	0.093	0.064	0.079	0.019	0.045	0.012		0.012	0.134	0.16
	Fourth	E	M	A	L	K	N	D	S	W	F	H	-	S	A	S
		0.095	0.048	0.054	0.1	0.005	0.037	0.019	0.059	0.017	0.03	0.011		0.011	0.041	0.049
AncHSPT_codeml	Best	Q	E	C	I	Q	D	E	Y	Y	T	D	-	G	F	G
		0.518	0.652	0.455	0.396	0.908	0.579	0.643	0.645	0.834	0.231	0.748		0.515	0.473	0.485
	second	E	T	A	V	M	E	Q	H	F	Y	N	-	C	Y	A
		0.259	0.102	0.232	0.263	0.031	0.375	0.116	0.196	0.157	0.2	0.195		0.163	0.246	0.288
	Third	K	D	S	M	K	G	D	F	H	S	E	-	A	L	S
		0.163	0.072	0.128	0.162	0.016	0.019	0.101	0.11	0.003	0.187	0.043		0.154	0.068	0.182
	Fourth	R	A	T	L	R	N	H	A	L	A	Q	-	S	A	P
		0.026	0.052	0.127	0.153	0.013	0.013	0.096	0.014	0.002	0.145	0.004		0.096	0.05	0.023
AncHSPT_phyml	Best	E	E	C	I	Q	D	E	Y	Y	S	D	-	G	F	A
		0.329	0.882	0.564	0.412	0.995	0.523	0.972	0.541	0.870	0.237	0.768		0.869	0.420	0.479
	second	K	T	A	V	M	E	Q	H	F	T	N	-	A	Y	S
		0.245	0.07	0.291	0.226	0.005	0.473	0.012	0.26	0.126	0.229	0.228		0.056	0.192	0.273
	Third	Q	A	T	M	R	G	H	F	H	Y	E	-	C	S	G
		0.245	0.028	0.087	0.221	1E-04	0.003	0.009	0.142	0.002	0.205	0.003		0.043	0.088	0.235
	Fourth	R	S	S	L	E	N	D	A	L	A	Q	-	S	A	P
		0.077	0.007	0.048	0.122	1E-04	8E-04	0.007	0.024	0.001	0.156	3E-04		0.025	0.084	0.011
AncHSPT_iqtree	Best	E	E	C	I	Q	D	E	Y	Y	S	D	-	G	F	A
		0.339	0.906	0.500	0.439	0.999	0.505	0.995	0.549	0.931	0.230	0.788		0.952	0.379	0.347
	second	K	T	A	M	M	E	Q	H	F	Y	N	-	A	S	G
		0.24	0.062	0.366	0.27	0.001	0.494	0.002	0.26	0.068	0.221	0.21		0.024	0.166	0.336
	Third	Q	A	T	V	K	G	D	F	H	T	E	-	C	Y	S
		0.222	0.022	0.090	0.208	0.000	0.001	0.001	0.161	0.001	0.210	0.001		0.012	0.117	0.288
	Fourth	R	S	S	L	R	N	H	A	L	A	Q	-	S	A	P
		0.083	0.005	0.034	0.071	0.000	0.000	0.001	0.016	0.000	0.152	0.000		0.010	0.115	0.021

AncHGSPT_nhphylobayes	Best	E	E	C	I	Q	E	E	F	Y	Y	D	-	G	P	G
		0.287	0.856	0.995	0.542	0.999	0.625	0.970	0.590	0.994	0.286	0.959		0.446	0.287	0.510
	second	K	T	A	V	M	D	H	H	P	A	N	-	C	I	S
		0.126	0.077	0.002	0.156	0.001	0.309	0.025	0.192	0.002	0.244	0.02		0.266	0.218	0.232
	Third	G	S	T	M	P	G	A	A	F	S	R	-	E	S	A
		0.122	0.018	0.001	0.107	0.000	0.048	0.001	0.128	0.001	0.161	0.007		0.248	0.13	0.179
	Fourth	I	A	E	K	-	A	Y	Y	A	T	K	-	S	F	P
		0.106	0.017	0.000	0.060		0.004	0.001	0.064	0.001	0.087	0.004		0.009	0.122	0.034
AncHGSPT_raxml	Best	E	E	C	I	Q	E	E	H	Y	S	D	-	C	F	S
		0.194	0.884	0.910	0.376	0.962	0.532	0.731	0.622	0.871	0.286	0.813		0.552	0.312	0.439
	second	Q	T	A	M	M	D	H	Y	F	T	N	-	G	S	A
		0.149	0.049	0.052	0.374	0.034	0.440	0.146	0.279	0.118	0.276	0.18		0.201	0.229	0.326
	Third	K	Q	T	V	H	G	Q	F	W	Y	E	-	A	Y	G
		0.145	0.021	0.021	0.164	0.001	0.015	0.079	0.064	0.004	0.195	0.004		0.093	0.135	0.165
	Fourth	D	A	S	L	K	N	D	A	H	A	H	-	S	P	P
		0.110	0.016	0.014	0.066	0.001	0.005	0.031	0.015	0.004	0.119	9E-04		0.062	0.113	0.05
AncHGSPT_codeml	Best	Q	E	C	I	Q	D	E	Y	Y	T	D	-	G	F	G
		0.503	0.654	0.454	0.400	0.906	0.570	0.649	0.642	0.836	0.227	0.749		0.513	0.465	0.483
	second	E	T	A	V	M	E	Q	H	F	Y	N	-	A	Y	A
		0.274	0.098	0.236	0.262	0.030	0.384	0.114	0.197	0.154	0.194	0.191		0.155	0.239	0.283
	Third	K	D	S	M	K	G	D	F	H	S	E	-	C	L	S
		0.160	0.072	0.127	0.158	0.016	0.018	0.102	0.112	0.004	0.188	0.044		0.154	0.077	0.188
	Fourth	R	A	T	L	R	N	H	A	L	A	Q	-	S	A	P
		0.026	0.051	0.124	0.153	0.013	0.013	0.091	0.013	0.002	0.152	0.004		0.096	0.049	0.022
AncHGSPT_phyml	Best	E	E	C	I	Q	E	E	Y	Y	S	D	-	G	F	A
		0.380	0.887	0.562	0.452	0.997	0.499	0.980	0.524	0.880	0.250	0.781		0.863	0.374	0.447
	second	K	T	A	V	M	D	Q	H	F	T	N	-	A	Y	S
		0.213	0.065	0.304	0.217	0.003	0.498	0.009	0.270	0.114	0.204	0.213		0.06	0.159	0.317
	Third	Q	A	T	M	E	G	D	F	H	A	E	-	C	L	G
		0.212	0.027	0.077	0.189	0.000	0.002	0.006	0.155	0.003	0.196	0.004		0.028	0.13	0.225
	Fourth	R	S	S	L	K	N	H	A	L	Y	S	-	S	A	P
		0.070	0.008	0.046	0.121	0.000	0.001	0.004	0.018	0.001	0.169	6E-04		0.026	0.079	0.009
AncHGSPT_iqtree	Best	E	E	C	I	Q	D	E	Y	Y	S	D	-	G	F	A
		0.339	0.906	0.500	0.439	0.999	0.505	0.995	0.549	0.931	0.230	0.788		0.952	0.379	0.347
	second	K	T	A	M	M	E	Q	H	F	Y	N	-	A	S	G
		0.240	0.062	0.366	0.270	0.001	0.494	0.002	0.260	0.068	0.221	0.21		0.024	0.166	0.336
	Third	Q	A	T	V	K	G	D	F	H	T	E	-	C	Y	S
		0.222	0.022	0.090	0.208	0.000	0.001	0.001	0.161	0.001	0.210	0.001		0.012	0.117	0.288
	Fourth	R	S	S	L	R	N	H	A	L	A	Q	-	S	A	P
		0.083	0.005	0.034	0.071	0.000	0.000	0.001	0.016	0.000	0.152	0.000		0.010	0.115	0.021
AncSTP_nhphylobayes	Best	K	T	C	M	Q	E	H	A	Y	Y	N	-	C	S	G
		0.217	0.545	0.999	0.398	0.814	0.542	0.972	0.729	0.950	0.887	0.765		0.972	0.488	0.757
	second	G	E	E	I	M	D	E	F	F	A	D	-	G	Y	P
		0.202	0.246	0.001	0.190	0.163	0.440	0.018	0.156	0.014	0.016	0.139		0.018	0.283	0.122
	Third	Q	A	S	H	F	G	V	H	L	T	E	-	R	A	A
		0.137	0.102	0.000	0.130	0.008	0.013	0.004	0.062	0.008	0.016	0.032		0.006	0.065	0.061
	Fourth	E	M	-	V	L	A	Y	Y	A	S	K	-	E	F	S
		0.102	0.070		0.096	0.004	0.001	0.003	0.043	0.007	0.012	0.030		0.002	0.042	0.035
AncSTP_raxml	Best	K	T	C	M	M	D	H	A	F	Y	N	-	C	S	G
		0.187	0.698	0.799	0.508	0.966	0.783	0.984	0.750	0.390	0.969	0.885		1.000	0.614	0.562
	second	R	M	E	H	Q	E	Y	S	M	F	Q	-	R	A	P
		0.136	0.173	0.067	0.172	0.014	0.214	0.014	0.104	0.178	0.015	0.037		0.000	0.190	0.356
	Third	Q	A	A	Q	L	N	Q	T	L	W	H	-	S	Y	A
		0.132	0.085	0.047	0.133	0.010	0.002	0.000	0.029	0.111	0.011	0.032		0.000	0.088	0.031
	Fourth	E	S	S	L	F	Q	N	C	Y	H	K	-	A	T	S
		0.087	0.022	0.030	0.055	0.003	0.001	0.000	0.025	0.108	0.004	0.022		0.000	0.038	0.029
AncSTP_codeml	Best	Q	T	C	M	Q	D	H	A	Y	Y	N	-	C	Y	G
		0.53	0.575	0.518	0.382	0.666	0.687	0.921	0.313	0.567	0.891	0.507		0.964	0.447	0.621
	second	K	A	A	L	M	E	Q	Y	F	F	D	-	G	F	A
		0.252	0.174	0.211	0.225	0.234	0.309	0.03	0.264	0.388	0.056	0.396		0.012	0.25	0.193
	Third	E	S	S	I	L	N	Y	H	L	H	E	-	A	A	S
		0.145	0.097	0.119	0.212	0.037	0.002	0.021	0.176	0.019	0.021	0.057		0.01	0.091	0.118
	Fourth	R	E	T	V	R	Q	E	S	I	S	K	-	S	S	P
		0.042	0.069	0.072	0.123	0.019	0.001	0.01	0.101	0.007	0.007	0.013		0.009	0.084	0.056
AncSTP_phyml	Best	K	T	C	M	Q	D	H	A	Y	Y	N	-	C	Y	G
		0.336	0.689	0.587	0.504	0.702	0.555	0.990	0.402	0.653	0.915	0.825		0.996	0.350	0.382
	second	Q	A	A	I	M	E	Y	H	F	F	D	-	G	F	A
		0.247	0.201	0.278	0.184	0.294	0.445	0.003	0.215	0.321	0.041	0.162		0.002	0.228	0.353
	Third	E	E	T	L	L	-	Q	Y	L	H	E	-	A	S	S
		0.225	0.062	0.068	0.175	0.002		0.003	0.183	0.010	0.017	0.008		0.001	0.161	0.203
	Fourth	R	M	S	V	K	-	E	S	M	S	Q	-	S	A	P
		0.096	0.022	0.051	0.092	0.001		0.003	0.080	0.005	0.008	0.003		0.001	0.142	0.061
AncSTP_iqtree	Best	K	T	C	M	Q	D	H	A	Y	Y	N	-	C	S	G
		0.325	0.719	0.519	0.628	0.712	0.522	0.995	0.556	0.751	0.906	0.894		0.998	0.297	0.478
	second	E	A	A	I	M	E	Y	H	F	F	D	-	G	Y	A
		0.235	0.192	0.353	0.163	0.286	0.478	0.002	0.188	0.231	0.043	0.099		0.001	0.248	0.251
	Third	Q	E	T	L	L	N	E	Y	L	H	E	-	R	A	S
		0.228	0.055	0.076	0.118	0.001	0.000	0.002	0.115	0.006	0.019	0.004		0.000	0.196	0.202
	Fourth	R	M	S	V	K	G	Q	F	M	S	Q	-	A	F	P
		0.103	0.020	0.037	0.061	0.001	0.000	0.001	0.061	0.005	0.008	0.002		0.000	0.179	0.064

AncSP_nhphylobayes	Best	G	T	C	M	Q	E	H	A	Y	Y	N	-	C	S	G
		0.275	0.921	0.977	0.630	0.535	0.630	0.971	0.840	0.741	0.931	0.800		0.992	0.459	0.996
	second	K	A	E	I	M	D	E	F	L	A	D	-	G	Y	P
		0.164	0.049	0.022	0.13	0.244	0.361	0.011	0.063	0.054	0.01	0.088		0.008	0.429	0.002
	Third	Q	E	L	H	F	G	V	Y	A	K	K	-	R	A	S
		0.151	0.019	0.000	0.060	0.099	0.003	0.010	0.044	0.051	0.009	0.047		0.000	0.034	0.001
	Fourth	E	M	W	F	W	Q	Y	H	E	F	E	-	-	F	A
		0.095	0.006	0.000	0.044	0.033	0.001	0.006	0.023	0.046	0.007	0.021			0.015	0.000
AncSP_raxml	Best	K	T	E	M	M	E	H	A	M	Y	N	-	C	Y	G
		0.097	0.966	0.953	0.702	0.551	0.868	0.478	0.453	0.277	0.746	0.484		0.999	0.806	0.998
	second	Q	S	D	L	W	D	Y	S	I	H	H	-	G	S	A
		0.092	0.018	0.018	0.112	0.148	0.125	0.340	0.194	0.202	0.100	0.214		0.000	0.047	0.001
	Third	R	A	Q	I	F	Q	V	T	V	F	K	-	A	H	S
		0.091	0.008	0.012	0.092	0.098	0.004	0.043	0.084	0.180	0.026	0.135		0.000	0.037	0.000
	Fourth	E	V	A	F	R	N	F	E	L	P	Q	-	S	C	N
		0.090	0.002	0.005	0.042	0.051	0.001	0.037	0.045	0.126	0.020	0.124		0.000	0.023	0.000
AncSP_codeml	Best	Q	T	C	M	Q	D	H	A	F	Y	N	-	C	Y	G
		0.56	0.751	0.343	0.443	0.529	0.616	0.904	0.427	0.238	0.92	0.626		0.99	0.578	0.955
	second	K	A	E	L	M	E	Y	S	Y	F	D	-	G	F	A
		0.237	0.108	0.275	0.23	0.273	0.381	0.052	0.167	0.188	0.035	0.236		0.005	0.162	0.027
	Third	E	S	A	I	L	N	Q	Y	L	H	E	-	A	S	S
		0.117	0.087	0.166	0.213	0.05	0.001	0.021	0.135	0.176	0.022	0.046		0.002	0.079	0.014
	Fourth	R	E	S	V	R	Q	N	H	I	S	K	-	S	A	P
		0.052	0.018	0.091	0.087	0.046	0.001	0.006	0.11	0.131	0.005	0.034		0.002	0.071	0.002
AncSP_phyml	Best	K	T	C	M	Q	E	H	A	F	Y	N	-	C	Y	G
		0.316	0.900	0.427	0.582	0.594	0.551	0.986	0.563	0.183	0.939	0.924		0.999	0.486	0.981
	second	Q	A	E	I	M	D	Y	S	Y	F	D	-	G	S	A
		0.275	0.088	0.332	0.173	0.354	0.449	0.009	0.155	0.170	0.022	0.050		0.001	0.153	0.013
	Third	E	S	A	L	K	-	Q	H	L	H	Q	-	-	F	S
		0.196	0.007	0.17	0.172	0.021		0.002	0.101	0.17	0.017	0.008			0.138	0.006
	Fourth	R	E	S	V	R	-	E	Y	I	S	H	-	-	A	P
		0.109	0.002	0.039	0.056	0.020		0.002	0.072	0.138	0.006	0.008			0.115	0.000
AncSP_iqtree	Best	K	T	C	M	Q	E	H	A	V	Y	N	-	C	Y	G
		0.307	0.907	0.410	0.694	0.618	0.578	0.993	0.719	0.211	0.929	0.956		1.000	0.345	0.964
	second	Q	A	E	I	M	D	Y	S	I	F	D	-	G	S	A
		0.254	0.088	0.340	0.147	0.340	0.422	0.005	0.123	0.161	0.026	0.029		0.000	0.297	0.021
	Third	E	S	A	L	K	-	E	H	M	H	Q	-	A	A	S
		0.208	0.002	0.197	0.116	0.020		0.001	0.071	0.155	0.020	0.005		0.000	0.173	0.014
	Fourth	R	E	S	V	R	-	Q	Y	Y	S	H	-	S	F	P
		0.115	0.001	0.027	0.033	0.017		0.001	0.038	0.146	0.007	0.005		0.000	0.108	0.001

		398	544	546	549	603	605	607	967	968	1027	1253	1361	1362	1363	1364
ComS_nhphylobayes		R	T	C	F	R	E	V	A	Y	Y	N	-	C	S	G
		0.165	0.894	0.976	0.271	0.280	0.914	0.918	0.802	0.317	0.336	0.332		0.967	0.558	0.999
ComS_raxml		Y	T	C	F	R	E	V	D	A	P	H	-	C	S	G
		0.092	0.469	0.486	0.389	0.474	0.929	0.836	0.267	0.247	0.462	0.578		0.666	0.499	0.944
ComS_codeml		R	T	C	L	K	E	V	A	V	P	H	-	C	S	G
		0.289	0.548	0.349	0.515	0.384	0.722	0.384	0.361	0.319	0.165	0.314		0.922	0.306	0.989
ComS_phyml		R	T	C	L	K	E	V	A	V	K	H	-	C	S	G
		0.254	0.866	0.431	0.541	0.476	0.935	0.910	0.497	0.360	0.186	0.475		0.993	0.399	0.999
ComS_iqtree		R	T	C	L	K	E	V	A	V	K	H	-	C	S	G
		0.242	0.892	0.414	0.533	0.481	0.962	0.969	0.685	0.472	0.174	0.485		0.997	0.538	0.996
ComP_nhphylobayes		G	T	E	M	Q	E	H	A	Y	Y	N	-	C	Y	G
		0.353	0.992	0.678	0.854	0.363	0.627	0.976	0.859	0.635	0.980	0.831		0.999	0.957	1.000
ComP_raxml		Q	T	E	M	F	E	H	A	M	Y	N	-	C	Y	G
		0.234	0.988	0.985	0.857	0.411	0.564	0.710	0.466	0.264	0.956	0.662		1.000	0.992	0.998
ComP_codeml		Q	T	E	M	M	D	H	A	L	Y	N	-	C	Y	G
		0.599	0.898	0.763	0.534	0.512	0.587	0.912	0.487	0.256	0.977	0.728		0.999	0.907	0.992
ComP_phyml		Q	T	E	M	M	E	H	A	L	Y	N	-	C	Y	G
		0.329	0.994	0.970	0.694	0.802	0.559	0.986	0.628	0.249	0.989	0.963		1.000	0.943	0.999
ComP_iqtree		Q	T	E	M	M	E	H	A	M	Y	N	-	C	Y	G
		0.311	0.997	0.986	0.798	0.842	0.582	0.992	0.752	0.234	0.987	0.980		1.000	0.967	0.997
ComT_nhphylobayes		K	M	C	H	Q	D	H	A	Y	Y	N	H	C	S	P
		0.405	0.357	1.000	0.394	0.803	0.969	0.999	0.739	0.931	0.867	0.790	0.998	0.959	0.592	0.987
ComT_raxml		K	M	C	H	M	D	H	H	F	Y	N	H	C	S	P
		0.296	0.737	1.000	0.699	0.655	0.990	0.997	0.353	0.502	0.500	0.317	0.997	0.723	0.440	0.990
ComT_codeml		K	T	C	Q	Q	D	H	A	Y	Y	N	H	C	A	P
		0.411	0.33	0.976	0.38	0.639	0.914	0.987	0.339	0.544	0.882	0.5	0.956	0.957	0.393	0.918
ComT_phyml		K	T	C	Q	Q	D	H	A	Y	Y	N	H	C	S	P
		0.437	0.413	0.997	0.466	0.693	0.971	1.000	0.419	0.625	0.909	0.830	0.997	0.996	0.421	0.985
ComT_iqtree		K	T	C	Q	Q	D	H	A	Y	Y	N	H	C	S	P
		0.426	0.429	0.998	0.517	0.707	0.982	1.000	0.567	0.718	0.902	0.898	0.998	0.998	0.501	0.971
ComG_nhphylobayes		E	E	C	I	Q	E	E	F	Y	A	D	-	E	P	S
		0.650	0.999	0.994	0.996	1.000	0.999	1.000	0.613	1.000	0.542	1.000		1.000	0.631	1.000
ComG_raxml		E	E	C	I	Q	E	E	H	Y	A	D	-	E	P	S
		0.735	0.999	0.634	0.996	0.999	0.999	0.999	0.839	0.999	0.628	0.999		0.999	0.834	0.998
ComG_codeml		E	E	A	I	Q	E	E	H	Y	A	D	-	E	L	S
		0.957	0.995	0.506	0.973	0.999	0.991	0.995	0.503	0.998	0.643	0.997		0.991	0.378	0.987
ComG_phyml		E	E	C	I	Q	E	E	H	Y	A	D	-	E	L	S
		0.941	1.000	0.578	0.988	1.000	0.999	1.000	0.500	0.999	0.593	1.000		0.999	0.418	0.999
ComG_iqtree		E	E	C	I	Q	E	E	H	Y	A	D	-	E	P	S
		0.940	1.000	0.513	0.995	1.000	0.999	1.000	0.518	1.000	0.584	1.000		1.000	0.405	0.997
ComH_nhphylobayes		Q	E	T	V	Q	G	E	Y	Y	T	D	-	G	F	A
		0.487	0.996	0.998	0.982	1.000	0.999	1.000	0.999	1.000	0.998	1.000		0.999	0.998	0.999
ComH_raxml		Q	E	T	V	Q	G	E	Y	Y	T	D	-	G	F	A
		0.515	0.983	0.999	0.948	0.999	0.999	0.998	0.999	0.999	0.999	0.999		1.000	0.998	0.998
ComH_codeml		Q	E	T	V	Q	G	E	Y	Y	T	D	-	G	F	A
		0.794	0.924	0.982	0.841	0.997	0.99	0.978	0.995	0.996	0.98	0.994		0.997	0.991	0.983
ComH_phyml		Q	E	T	V	Q	G	E	Y	Y	T	D	-	G	F	A
		0.654	0.999	0.999	0.925	1.000	0.998	1.000	0.997	0.999	0.991	0.999		1.000	0.996	0.999
ComH_iqtree		Q	E	T	V	Q	G	E	Y	Y	T	D	-	G	F	A
		0.620	1.000	0.999	0.959	1.000	0.999	1.000	0.998	1.000	0.989	1.000		1.000	0.998	0.996
ComK_nhphylobayes		G	S	E	L	M	E	Y	F	P	V	E	-	G	I	G
		1.000	0.923	1.000	1.000	1.000	1.000	1.000	0.995	1.000	0.530	1.000		1.000	0.583	1.000
ComK_raxml		G	A	E	L	M	E	Y	F	P	L	E	-	G	L	G
		1.000	0.642	1.000	0.999	1.000	1.000	1.000	0.734	1.000	0.444	1.000		1.000	0.417	1.000
ComK_codeml		G	S	E	L	M	E	Y	F	P	L	E	-	G	L	G
		1	0.506	0.997	0.994	0.999	0.995	0.999	0.775	1	0.394	0.997		1	0.531	1
ComK_phyml		G	S	E	L	M	E	Y	F	P	V	E	-	G	L	G
		1.000	0.805	1.000	0.998	1.000	1.000	1.000	0.892	1.000	0.527	1.000		1.000	0.504	1.000
ComK_iqtree		G	S	E	L	M	E	Y	F	P	V	E	-	G	I	G
		1.000	0.893	1.000	0.999	1.000	1.000	1.000	0.945	1.000	0.476	1.000		1.000	0.537	1.000

(c)Based on the ML tree from alignment B

		408	541	543	546	597	599	601	935	978	979	1176	1305	1307	1308	1309
AncDK_nhphylobayes	Best	E	S	Q	K	Q	D	E	Y	K	L	R	-	G	V	A
		0.916	0.975	0.994	0.888	0.990	0.998	0.998	0.744	0.212	0.673	1		1	0.247	0.659
	second	G	A	T	L	S	E	M	A	A	V	-	-	-	W	G
		0.037	0.018	0.005	0.048	0.006	0.002	0.002	0.175	0.146	0.079				0.154	0.33
	Third	F	E	E	S	M	-	-	E	Y	F	-	-	-	I	S
		0.032	0.007	0.001	0.017	0.004			0.054	0.123	0.059				0.144	0.004
	Fourth	W	-	-	Y	-	-	-	N	Q	Y	-	-	-	F	E
		0.006			0.010				0.006	0.097	0.039				0.119	0.002
AncDK_raxml	Best	E	S	Q	K	Q	D	E	E	V	L	R	-	G	I	A
		0.993	0.945	0.995	0.992	0.845	0.993	0.992	0.606	0.291	0.533	0.992		1	0.661	0.596
	second	D	A	E	R	M	E	Q	Y	I	M	K	-	A	F	G
		0.004	0.051	0.003	0.004	0.057	0.006	0.004	0.078	0.216	0.148	0.005		1E-04	0.1	0.384
	Third	Q	T	H	Q	S	N	D	Q	K	I	Q	-	S	L	S
		0.001	0.003	0.000	0.002	0.056	0.001	0.002	0.062	0.080	0.128	0.002		1E-04	0.067	0.013
	Fourth	G	N	K	M	T	S	K	A	M	R	H	-	N	V	C
		9E-04	0.001	0.000	5E-04	0.013	0.000	0.001	0.056	0.074	0.059	4E-04		7E-05	0.057	0.002
AncDK_codeml	Best	E	S	E	K	Q	D	E	E	V	L	R	-	G	I	A
		0.936	0.761	0.693	0.926	0.801	0.958	0.962	0.680	0.374	0.761	0.951		0.996	0.599	0.562
	second	D	A	Q	R	S	E	D	Y	I	I	K	-	A	L	G
		0.027	0.188	0.216	0.037	0.047	0.036	0.019	0.065	0.242	0.095	0.038		0.002	0.141	0.391
	Third	Q	T	D	Q	E	N	Q	A	K	R	Q	-	S	F	S
		0.014	0.026	0.026	0.011	0.024	0.004	0.012	0.051	0.084	0.043	0.006		0.001	0.124	0.033
	Fourth	A	N	K	L	K	-	K	D	L	M	N	-	-	V	T
		0.006	0.008	0.026	0.004	0.024		0.003	0.050	0.059	0.036	0.001			0.074	0.003
AncDK_phyml	Best	E	S	Q	K	Q	D	E	E	V	L	R	-	G	I	A
		0.993	0.934	0.994	0.99	0.979	0.991	0.998	0.783	0.375	0.823	0.998		1	0.675	0.76
	second	D	A	E	M	S	E	Q	Y	I	I	K	-	A	L	G
		0.002	0.063	0.005	0.004	0.009	0.009	0.001	0.117	0.279	0.086	0.002		2E-04	0.113	0.225
	Third	Q	T	K	R	M	N	D	A	K	R	Q	-	S	F	S
		0.002	0.002	0.000	0.003	0.004	0.000	0.001	0.040	0.095	0.028	3E-04		1E-04	0.11	0.014
	Fourth	A	N	A	Q	T	-	K	D	L	M	E	-	-	V	T
		0.001	0.001	0.000	0.001	0.002		0.000	0.019	0.049	0.024	1E-04			0.059	4E-04
AncDK_iqtree	Best	E	S	Q	K	Q	D	E	E	V	L	R	-	G	I	A
		0.985	0.979	0.999	0.982	0.984	0.995	1.000	0.756	0.376	0.833	0.998		1	0.642	0.78
	second	D	A	E	R	S	E	Q	Y	I	I	K	-	A	F	G
		0.006	0.020	0.001	0.008	0.007	0.005	0.000	0.106	0.269	0.085	0.001		3E-05	0.255	0.206
	Third	Q	E	T	M	M	-	D	A	K	R	Q	-	-	L	S
		0.004	0.000	0.000	0.003	0.003		0.000	0.048	0.090	0.028	2E-04			0.064	0.013
	Fourth	A	T	A	Q	T	-	-	D	L	M	E	-	-	V	T
		0.002	0.000	0.000	0.003	0.001			0.025	0.053	0.021	4E-05			0.022	3E-04
AncHSPT_nhphylobayes	Best	F	E	T	I	Q	D	E	Y	Y	S	R	-	G	P	A
		0.939	0.946	0.976	0.638	1.000	0.969	0.982	0.790	0.947	0.987	0.602		0.95	0.995	0.708
	second	W	A	C	S	-	E	H	A	K	H	V	-	A	E	G
		0.054	0.036	0.014	0.133		0.028	0.017	0.139	0.010	0.003	0.172		0.028	0.003	0.264
	Third	E	S	A	K	-	G	M	D	E	G	G	-	E	F	S
		0.005	0.008	0.004	0.071		0.003	0.001	0.020	0.009	0.002	0.135		0.014	0.001	0.012
	Fourth	H	T	A	M	-	-	-	E	G	G	I	-	C	F	I
		0.001	0.007	0.004	0.035				0.016	0.007	0.002	0.067		0.008	0.001	0.004
AncHSPT_raxml	Best	W	T	T	M	M	D	H	Y	Y	W	V	-	C	F	A
		0.838	0.469	0.382	0.481	0.513	0.634	0.789	0.357	0.7	0.562	0.414		0.564	0.578	0.46
	second	F	E	C	V	Q	G	Q	D	F	Y	G	-	G	Y	G
		0.114	0.175	0.343	0.221	0.454	0.228	0.073	0.243	0.187	0.212	0.142		0.183	0.366	0.304
	Third	Y	A	S	I	L	E	E	H	H	F	A	-	A	I	M
		0.033	0.103	0.116	0.16	0.006	0.063	0.067	0.152	0.042	0.04	0.14		0.18	0.027	0.052
	Fourth	L	M	A	L	H	N	D	N	W	H	I	-	S	L	S
		0.005	0.084	0.068	0.039	0.005	0.042	0.021	0.091	0.026	0.037	0.125		0.057	0.009	0.048
AncHSPT_codeml	Best	F	E	A	M	Q	D	E	D	Y	T	R	-	G	F	A
		0.646	0.551	0.343	0.305	0.891	0.644	0.522	0.262	0.875	0.208	0.196		0.492	0.525	0.408
	second	W	T	T	I	R	E	Q	E	F	S	A	-	A	Y	G
		0.243	0.135	0.223	0.232	0.024	0.287	0.15	0.151	0.112	0.195	0.181		0.237	0.264	0.346
	Third	Y	D	S	V	K	G	H	Y	H	Y	V	-	S	L	S
		0.093	0.07	0.206	0.21	0.022	0.027	0.148	0.147	0.007	0.184	0.137		0.108	0.084	0.173
	Fourth	L	A	C	L	M	N	D	N	W	W	K	-	C	I	T
		0.012	0.068	0.088	0.178	0.02	0.021	0.11	0.113	0.002	0.111	0.084		0.079	0.037	0.028
AncHSPT_phyml	Best	F	E	T	M	Q	D	E	D	Y	S	R	-	G	F	A
		0.887	0.826	0.436	0.846	0.991	0.719	0.9	0.409	0.898	0.245	0.541		0.644	0.5	0.623
	second	W	T	A	I	M	E	Q	E	F	T	V	-	A	Y	S
		0.092	0.087	0.413	0.077	0.008	0.27	0.054	0.277	0.093	0.207	0.119		0.284	0.24	0.219
	Third	Y	A	S	V	K	G	H	Y	H	Y	A	-	S	L	G
		0.016	0.039	0.103	0.049	4E-04	0.006	0.027	0.148	0.006	0.167	0.111		0.043	0.105	0.146
	Fourth	L	S	C	L	R	N	D	N	W	W	G	-	E	I	T
		0.004	0.017	0.019	0.014	3E-04	0.003	0.016	0.051	0.002	0.103	0.075		0.012	0.051	0.006
AncHSPT_iqtree	Best	F	E	T	M	Q	D	E	D	Y	S	R	-	G	F	A
		0.824	0.932	0.791	0.705	0.993	0.76	0.992	0.381	0.88	0.276	0.586		0.869	0.612	0.662
	second	W	T	A	I	M	E	Q	E	F	T	V	-	A	Y	S
		0.13	0.041	0.186	0.12	0.006	0.238	0.004	0.211	0.107	0.252	0.105		0.122	0.183	0.194
	Third	Y	A	S	V	K	G	H	Y	H	Y	A	-	S	L	G
		0.036	0.016	0.014	0.095	2E-04	0.001	0.003	0.151	0.007	0.148	0.101		0.005	0.077	0.135
	Fourth	L	S	Q	L	R	N	D	N	W	W	G	-	E	I	T
		0.007	0.009	0.004	0.044	2E-04	2E-04	7E-04	0.082	0.002	0.081	0.077		0.003	0.069	0.004

AncHGSPT_nhphylobayes	Best	F	E	T	K	Q	D	E	Y	K	L	R	-	G	V	A
		0.941	0.946	0.975	0.888	1.000	0.968	0.996	0.779	0.212	0.673	1		0.952	0.247	0.699
	second	W	A	A	L	-	E	H	A	A	V	-	-	A	W	G
		0.048	0.034	0.008	0.048		0.029	0.003	0.149	0.146	0.079			0.026	0.154	0.261
	Third	E	S	A	S	-	G	M	E	Y	F	-	-	E	I	S
		0.009	0.010	0.008	0.017		0.002	0.001	0.023	0.123	0.059			0.019	0.144	0.026
	Fourth	H	T	A	E	-	V	-	D	Q	Y	-	-	C	F	I
		0.002	0.007	0.008	0.011		0.001		0.014	0.097	0.039			0.003	0.119	0.004
AncHGSPT_raxml	Best	F	E	A	M	Q	E	E	Y	Y	S	R	-	E	F	S
		0.823	0.911	0.409	0.898	0.975	0.584	0.640	0.330	0.918	0.362	0.504		0.335	0.317	0.581
	second	W	T	T	I	M	D	Q	D	F	W	V	-	A	P	A
		0.160	0.028	0.383	0.068	0.018	0.386	0.187	0.224	0.058	0.154	0.088		0.298	0.314	0.273
	Third	Y	Q	C	V	H	G	H	H	H	T	A	-	G	Y	G
		0.015	0.020	0.105	0.021	0.002	0.012	0.135	0.145	0.013	0.141	0.077		0.131	0.209	0.083
	Fourth	L	D	S	L	E	N	D	N	W	Y	G	-	S	H	T
		0.001	0.015	0.080	0.007	0.001	0.006	0.024	0.090	0.008	0.092	0.07		0.075	0.035	0.022
AncHGSPT_codeml	Best	F	E	A	M	Q	D	E	D	Y	S	W	-	G	F	S
		0.687	0.616	0.465	0.333	0.870	0.481	0.681	0.229	0.865	0.284	0.375		0.452	0.305	0.314
	second	W	D	S	I	E	E	Q	E	F	T	L	-	A	L	A
		0.146	0.083	0.175	0.228	0.031	0.469	0.108	0.183	0.113	0.172	0.293		0.175	0.163	0.297
	Third	Y	T	T	L	K	N	D	Y	H	Y	F	-	E	Y	G
		0.122	0.071	0.149	0.167	0.030	0.015	0.107	0.112	0.011	0.111	0.085		0.133	0.157	0.286
	Fourth	L	A	C	V	R	G	H	N	L	A	M	-	S	P	T
		0.023	0.058	0.046	0.166	0.026	0.010	0.043	0.093	0.003	0.083	0.052		0.081	0.11	0.045
AncHGSPT_phyml	Best	F	E	A	M	Q	D	E	D	Y	S	R	-	G	F	A
		0.907	0.845	0.485	0.862	0.997	0.622	0.943	0.366	0.896	0.345	0.928		0.641	0.305	0.51
	second	W	T	T	I	M	E	Q	E	F	T	K	-	A	L	S
		0.064	0.066	0.389	0.076	0.002	0.374	0.042	0.318	0.089	0.170	0.031		0.229	0.164	0.355
	Third	Y	A	S	V	E	N	D	Y	H	Y	A	-	E	Y	G
		0.021	0.034	0.084	0.031	5E-04	0.002	0.009	0.139	0.008	0.108	0.01		0.072	0.15	0.123
	Fourth	L	S	Q	L	K	G	H	N	W	A	G	-	S	P	T
		0.005	0.025	0.013	0.011	4E-04	0.002	0.005	0.046	0.002	0.078	0.006		0.037	0.125	0.007
AncHGSPT_iqtree	Best	F	E	T	M	Q	D	E	D	Y	S	R	-	G	F	A
		0.850	0.935	0.772	0.729	0.998	0.736	0.996	0.343	0.875	0.369	0.943		0.872	0.459	0.564
	second	W	T	A	I	M	E	Q	E	F	T	K	-	A	L	S
		0.088	0.034	0.206	0.116	0.001	0.263	0.003	0.249	0.105	0.205	0.025		0.106	0.142	0.31
	Third	Y	A	S	V	E	G	D	Y	H	Y	A	-	E	Y	G
		0.046	0.014	0.012	0.067	0.000	0.000	0.000	0.137	0.010	0.099	0.008		0.016	0.133	0.117
	Fourth	L	S	Q	L	K	N	D	N	W	A	G	-	S	V	T
		0.009	0.014	0.007	0.039	0.000	0.000	0.000	0.073	0.003	0.070	0.005		0.005	0.069	0.005
AncSTP_nhphylobayes	Best	W	E	C	I	Q	D	H	Y	Y	S	V	-	A	P	G
		0.757	0.406	0.835	0.599	0.986	0.971	0.960	0.584	1.000	0.419	0.766		0.393	0.852	0.467
	second	F	A	T	S	M	E	E	A	-	Y	I	-	G	Y	A
		0.165	0.279	0.103	0.060	0.013	0.029	0.036	0.156		0.191	0.199		0.358	0.038	0.367
	Third	L	T	E	Q	F	-	V	D	-	W	R	-	C	I	I
		0.027	0.236	0.057	0.054	0.001		0.002	0.093		0.121	0.021		0.22	0.024	0.076
	Fourth	P	M	M	M	-	-	V	E	-	M	G	-	E	E	F
		0.019	0.066	0.002	0.049			0.002	0.047		0.050	0.008		0.015	0.018	0.029
AncSTP_raxml	Best	W	T	C	M	M	D	H	D	Y	W	I	-	C	Y	G
		0.976	0.593	0.658	0.454	0.968	0.86	0.981	0.552	0.235	0.777	0.499		0.87	0.523	0.374
	second	F	M	E	H	Q	E	Y	E	F	Y	V	-	A	I	F
		0.013	0.286	0.095	0.243	0.02	0.137	0.018	0.163	0.18	0.211	0.493		0.095	0.241	0.237
	Third	Y	A	A	Q	W	N	Q	N	H	F	L	-	S	F	M
		0.005	0.074	0.076	0.2	0.004	0.002	3E-04	0.081	0.107	0.009	0.003		0.028	0.098	0.133
	Fourth	L	S	S	I	L	Q	N	Q	C	H	M	-	T	V	L
		0.002	0.02	0.053	0.029	0.002	7E-04	3E-04	0.03	0.059	0.002	0.003		0.002	0.04	0.048
AncSTP_codeml	Best	W	T	A	M	Q	D	H	D	Y	Y	V	-	A	F	G
		0.592	0.564	0.323	0.395	0.77	0.773	0.925	0.383	0.761	0.54	0.532		0.359	0.419	0.476
	second	F	A	S	L	M	E	Y	E	F	W	I	-	C	Y	A
		0.359	0.155	0.2	0.204	0.124	0.22	0.029	0.172	0.198	0.404	0.347		0.353	0.382	0.317
	Third	Y	S	C	I	R	N	Q	N	H	F	L	-	S	L	S
		0.038	0.099	0.174	0.176	0.037	0.004	0.022	0.113	0.017	0.034	0.043		0.144	0.066	0.118
	Fourth	L	E	T	V	K	Q	N	H	A	H	A	-	G	I	T
		0.008	0.067	0.125	0.125	0.022	0.001	0.008	0.069	0.004	0.011	0.024		0.12	0.05	0.02
AncSTP_phyml	Best	W	T	A	M	Q	D	H	D	Y	Y	V	-	A	F	A
		0.606	0.647	0.413	0.864	0.752	0.777	0.987	0.525	0.791	0.538	0.669		0.618	0.411	0.512
	second	F	A	T	I	M	E	Y	E	F	W	I	-	C	Y	G
		0.374	0.195	0.325	0.069	0.244	0.223	0.006	0.266	0.177	0.418	0.315		0.176	0.339	0.27
	Third	L	E	S	V	R	N	Q	Y	H	F	L	-	G	L	S
		0.013	0.069	0.14	0.029	0.001	2E-04	0.004	0.067	0.015	0.027	0.004		0.103	0.083	0.166
	Fourth	M	M	C	L	L	G	E	N	A	H	A	-	S	I	F
		0.004	0.039	0.064	0.017	7E-04	1E-04	0.002	0.048	0.003	0.008	0.004		0.1	0.078	0.01
AncSTP_iqtree	Best	W	T	T	M	Q	D	H	D	Y	Y	V	-	A	F	A
		0.607	0.68	0.72	0.747	0.739	0.774	0.998	0.516	0.758	0.565	0.677		0.668	0.483	0.538
	second	F	A	A	I	M	E	Y	E	F	W	I	-	C	Y	G
		0.366	0.192	0.198	0.101	0.258	0.226	0.002	0.205	0.198	0.4	0.31		0.177	0.305	0.265
	Third	L	E	S	V	R	G	E	N	H	F	L	-	G	I	S
		0.014	0.069	0.04	0.053	0.001	2E-05	4E-04	0.076	0.019	0.022	0.004		0.119	0.106	0.145
	Fourth	Y	M	C	L	L	N	Q	Y	A	H	A	-	S	L	V
		0.009	0.045	0.019	0.051	6E-04	1E-05	2E-04	0.065	0.004	0.007	0.003		0.035	0.06	0.01

AncSP_nhphylobayes	Best	W	T	C	I	Q	D	H	Y	Y	Y	V	-	C	Y	G
		0.757	0.816	0.772	0.749	0.330	0.760	0.961	0.295	0.581	0.491	0.759		0.993	0.517	0.994
	second	L	A	E	F	W	E	V	D	V	W	I	-	G	P	A
		0.100	0.182	0.221	0.040	0.210	0.240	0.017	0.231	0.184	0.107	0.216		0.007	0.16	0.004
	Third	F	E	T	M	R	-	E	A	E	S	R	-	-	A	F
		0.072	0.002	0.007	0.038	0.154		0.011	0.182	0.048	0.094	0.008			0.058	0.001
	Fourth	M	-	-	H	F	-	Y	E	F	M	Y	-	-	E	F
		0.054			0.033	0.151		0.010	0.090	0.041	0.062	0.007			0.057	0.001
AncSP_raxml	Best	W	T	E	M	M	E	Y	D	G	W	V	-	C	Y	G
		0.515	0.994	0.991	0.581	0.503	0.831	0.502	0.667	0.29	0.566	0.538		1	0.977	1
	second	F	S	D	I	W	D	H	E	E	Y	I	-	A	H	A
		0.273	0.004	0.004	0.233	0.384	0.167	0.455	0.154	0.157	0.309	0.355		2E-05	0.006	7E-05
	Third	L	A	Q	L	F	Q	F	N	A	F	C	-	A	F	S
		0.097	0.001	0.003	0.091	0.04	0.001	0.019	0.06	0.11	0.064	0.068		2E-05	0.005	5E-05
	Fourth	M	V	A	V	R	N	V	S	D	H	F	-	G	S	N
		0.061	3E-04	6E-04	0.063	0.027	4E-04	0.005	0.02	0.099	0.029	0.013		2E-05	0.004	3E-05
AncSP_codeml	Best	W	T	E	M	Q	D	H	D	A	Y	V	-	C	Y	G
		0.587	0.848	0.701	0.452	0.49	0.669	0.872	0.512	0.279	0.551	0.539		0.964	0.77	0.982
	second	F	S	A	I	M	E	Y	E	Y	W	I	-	A	F	A
		0.369	0.068	0.09	0.238	0.222	0.328	0.11	0.18	0.109	0.408	0.391		0.017	0.172	0.013
	Third	Y	A	S	L	R	N	Q	N	G	F	L	-	G	L	S
		0.026	0.055	0.049	0.197	0.105	0.002	0.007	0.098	0.107	0.026	0.034		0.01	0.011	0.004
	Fourth	L	E	D	V	K	Q	N	H	G	H	M	-	S	V	-
		0.015	0.006	0.047	0.09	0.041	0.001	0.003	0.042	0.107	0.009	0.011		0.007	0.009	
AncSP_phyml	Best	W	T	E	M	Q	D	H	D	A	Y	V	-	C	Y	G
		0.602	0.981	0.948	0.868	0.461	0.624	0.968	0.647	0.287	0.548	0.682		0.995	0.834	0.997
	second	F	A	A	I	M	E	Y	E	G	W	I	-	A	F	A
		0.352	0.014	0.027	0.097	0.451	0.376	0.031	0.254	0.135	0.423	0.312		0.003	0.128	0.002
	Third	L	S	T	V	R	-	Q	N	E	F	L	-	G	L	S
		0.034	0.004	0.009	0.018	0.044		7E-04	0.033	0.122	0.02	0.002		0.002	0.007	6E-04
	Fourth	M	M	S	L	K	-	N	H	S	H	C	-	S	V	-
		0.01	4E-04	0.007	0.017	0.014		2E-04	0.014	0.109	0.006	0.002		5E-04	0.006	
AncSP_iqtree	Best	W	T	E	M	M	D	H	D	A	Y	V	-	C	Y	G
		0.601	0.999	0.998	0.769	0.476	0.6	0.992	0.661	0.262	0.574	0.692		1	0.92	0.999
	second	F	A	A	I	Q	E	Y	E	G	W	I	-	A	F	A
		0.351	0.001	1E-03	0.152	0.439	0.4	0.008	0.194	0.143	0.404	0.304		4E-05	0.074	9E-04
	Third	L	S	T	L	R	-	Q	N	E	F	L	-	G	H	S
		0.036	6E-05	7E-04	0.044	0.045		3E-05	0.053	0.124	0.015	0.001		2E-05	0.001	2E-04
	Fourth	M	M	S	V	W	-	E	H	S	H	C	-	-	I	N
		0.008	1E-05	1E-04	0.031	0.014		2E-05	0.022	0.109	0.005	0.001			0.001	1E-05

		408	541	543	546	597	599	601	935	978	979	1176	1305	1307	1308	1309
ComS_nhphylobayes		M	T	C	F	R	E	V	A	V	Y	Y	-	C	A	G
		0.860	0.809	0.811	0.631	0.743	0.990	0.992	0.294	0.683	0.676	0.281		0.993	0.639	1
ComS_raxml		M	T	C	F	R	E	V	E	V	P	G	-	C	S	G
		0.715	0.458	0.527	0.434	0.501	0.942	0.845	0.075	0.231	0.442	0.138		0.7	0.467	0.992
ComS_codeml		M	T	E	L	R	E	V	E	A	P	C	-	C	A	G
		0.276	0.511	0.465	0.352	0.417	0.675	0.251	0.312	0.377	0.16	0.194		0.883	0.222	0.989
ComS_phyml		M	T	E	M	K	E	V	E	A	P	C	-	C	S	G
		0.92	0.891	0.867	0.303	0.452	0.903	0.811	0.501	0.45	0.17	0.419		0.983	0.268	1
ComS_iqtree		M	T	E	L	K	E	V	E	A	P	C	-	C	S	G
		0.785	0.985	0.984	0.261	0.454	0.973	0.984	0.426	0.397	0.175	0.427		0.999	0.414	1
ComP_nhphylobayes		W	T	E	I	W	D	H	D	Y	Y	V	-	C	Y	G
		0.774	1	0.986	0.945	0.354	0.759	0.972	0.38	0.265	0.643	0.767		1	0.86	1
ComP_raxml		W	T	E	M	W	D	H	D	G	W	V	-	C	Y	G
		0.729	0.988	0.985	0.62	0.447	0.506	0.633	0.802	0.189	0.62	0.496		1	0.993	0.998
ComP_codeml		W	T	E	M	Q	D	H	D	A	Y	V	-	C	Y	G
		0.589	0.88	0.792	0.462	0.399	0.663	0.872	0.528	0.289	0.555	0.542		0.988	0.842	0.99
ComP_phyml		W	T	E	M	M	D	H	D	A	Y	V	-	C	Y	G
		0.603	0.989	0.979	0.868	0.512	0.623	0.968	0.655	0.289	0.55	0.682		0.999	0.87	0.999
ComP_iqtree		W	T	E	M	M	D	H	D	A	Y	V	-	C	Y	G
		0.601	0.999	0.998	0.769	0.476	0.6	0.992	0.661	0.262	0.574	0.692		1	0.92	0.999
ComT_nhphylobayes		W	A	C	I	Q	D	H	-	Y	Y	V	H	A	P	I
		0.82	0.433	0.996	0.23	0.984	1	0.999		0.773	0.256	0.79	0.997	0.985	0.764	0.274
ComT_raxml		W	M	C	H	M	D	H	-	F	W	I	H	A	I	F
		0.782	0.773	1	0.734	0.487	0.992	0.998		0.546	0.646	0.59	0.998	0.782	0.517	0.418
ComT_codeml		W	T	C	H	Q	D	H	-	Y	Y	I	H	A	I	F
		0.735	0.349	0.942	0.347	0.782	0.921	0.983		0.695	0.548	0.475	0.933	0.562	0.285	0.2
ComT_phyml		W	T	C	Q	Q	D	H	-	Y	Y	V	H	A	I	F
		0.774	0.403	0.989	0.553	0.751	0.978	1		0.728	0.545	0.625	0.997	0.817	0.397	0.281
ComT_iqtree		W	T	C	Q	Q	D	H	-	Y	Y	V	H	A	I	F
		0.78	0.407	0.998	0.504	0.738	0.995	1		0.689	0.571	0.633	1	0.936	0.628	0.272
ComG_nhphylobayes		F	E	T	I	Q	E	E	-	Y	S	R	-	E	P	S
		1	0.999	0.977	0.638	1	0.998	0.996		0.947	0.987	1		1	0.997	0.999
ComG_raxml		F	E	A	M	Q	E	E	-	Y	S	R	-	E	P	S
		1	0.999	0.843	0.797	1	0.999	0.832		1	0.999	1		0.999	1	0.999
ComG_codeml		F	E	A	M	Q	E	E	-	Y	S	R	-	E	P	S
		0.997	0.996	0.912	0.558	0.999	0.994	0.948		0.999	0.99	0.996		0.994	0.999	0.991
ComG_phyml		F	E	A	M	Q	E	E	-	Y	S	R	-	E	P	S
		1	1	0.661	0.887	1	0.998	0.959		0.999	0.992	1		0.999	0.999	0.998
ComG_iqtree		F	E	T	M	Q	E	E	-	Y	S	R	-	E	P	S
		0.999	1	0.738	0.806	1	0.999	0.994		0.999	0.994	1		1	1	0.998
ComH_nhphylobayes		F	E	T	V	Q	G	E	Y	Y	T	G	-	G	F	A
		1	1	1	1	1	1	1	1	1	0.999	1		1	0.997	1
ComH_raxml		F	E	T	V	Q	G	E	Y	Y	T	G	-	G	F	A
		0.889	0.999	1	0.998	1	1	0.999	1	1	1	1		1	0.997	0.999
ComH_codeml		F	E	T	V	Q	G	E	Y	Y	T	G	-	G	F	A
		0.967	0.995	0.992	0.976	0.998	0.998	0.995	0.997	0.998	0.992	0.998		0.999	0.996	0.993
ComH_phyml		F	E	T	V	Q	G	E	Y	Y	T	G	-	G	F	A
		0.992	1	0.999	0.998	1	1	1	1	0.999	0.996	1		1	0.998	0.999
ComH_iqtree		F	E	T	V	Q	G	E	Y	Y	T	G	-	G	F	A
		0.984	1	1	0.995	1	1	1	0.999	0.999	0.997	1		1	0.999	1
ComK_nhphylobayes		G	S	E	L	M	E	M	A	R	L	E	-	G	I	G
		0.999	0.983	1	0.999	0.984	1	0.509	1	0.994	0.993	0.999		1	0.48	1
ComK_raxml		G	S	E	L	M	E	M	A	R	L	E	-	G	I	G
		1	0.61	1	0.999	0.994	1	0.622	0.999	0.384	0.679	1		1	0.556	1
ComK_codeml		G	S	E	L	M	E	M	A	P	L	E	-	G	I	G
		0.999	0.712	0.995	0.993	0.982	0.993	0.524	0.995	1	0.961	0.995		1	0.417	1
ComK_phyml		G	S	E	L	M	E	M	A	R	L	E	-	G	I	G
		1	0.919	0.999	1	0.999	0.999	0.645	1	0.807	0.973	1		1	0.498	1
ComK_iqtree		G	S	E	L	M	E	M	A	R	L	E	-	G	I	G
		1	0.976	1	0.999	0.999	1	0.617	0.999	0.8	0.981	1		1	0.476	1
